# Synthesis of substituted 3,4-dihydroquinazolinones *via* a metal free Leuckart–Wallach type reaction†

**DOI:** 10.1039/d0ra10142g

**Published:** 2020-12-23

**Authors:** Suvarna Bokale-Shivale, Mohammad A. Amin, Rajiv T. Sawant, Marc Y. Stevens, Lewend Turanli, Adam Hallberg, Suresh B. Waghmode, Luke R. Odell

**Affiliations:** Department of Medicinal Chemistry, Uppsala Biomedical Center, Uppsala University P. O. Box 574 SE-751 23 Uppsala Sweden luke.odell@ilk.uu.se; Department of Chemistry, Savitribai Phule Pune University (formerly Pune University) Ganeshkhind Pune 411 007 India

## Abstract

The 3,4-dihydroquinazolinone (DHQ) moiety is a highly valued scaffold in medicinal chemistry due to the vast number of biologically-active compounds based on this core structure. Current synthetic methods to access these compounds are limited in terms of diversity and flexibility and often require the use of toxic reagents or expensive transition-metal catalysts. Herein, we describe the discovery and development of a novel cascade cyclization/Leuckart–Wallach type strategy to prepare substituted DHQs in a modular and efficient process using readily-available starting materials. Notably, the reaction requires only the addition of formic acid or acetic acid/formic acid and produces H_2_O, CO_2_ and methanol as the sole reaction byproducts. Overall, the reaction provides an attractive entry point into this important class of compounds and could even be extended to isotopic labelling *via* the site-selective incorporation of a deuterium atom.

## Abstract 2

A novel cascade cyclization/Leuckart–Wallach type strategy to prepare biologically important 3,4-dihydroquinazolinones is presented.

## Graphical abstract

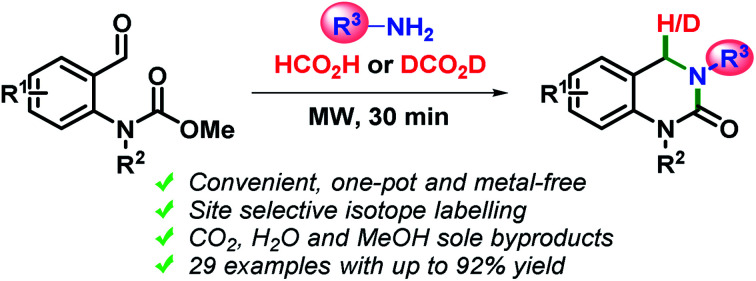

## Introduction

**Fig. 1:**
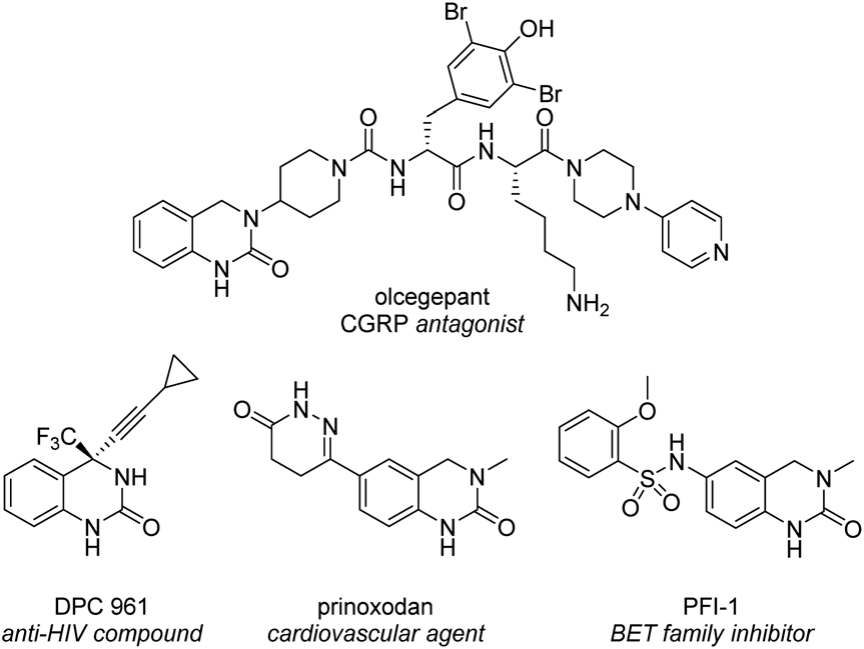
Representative biologically-active 3,4-dihydroquinazolinones.

**Scheme 1:**
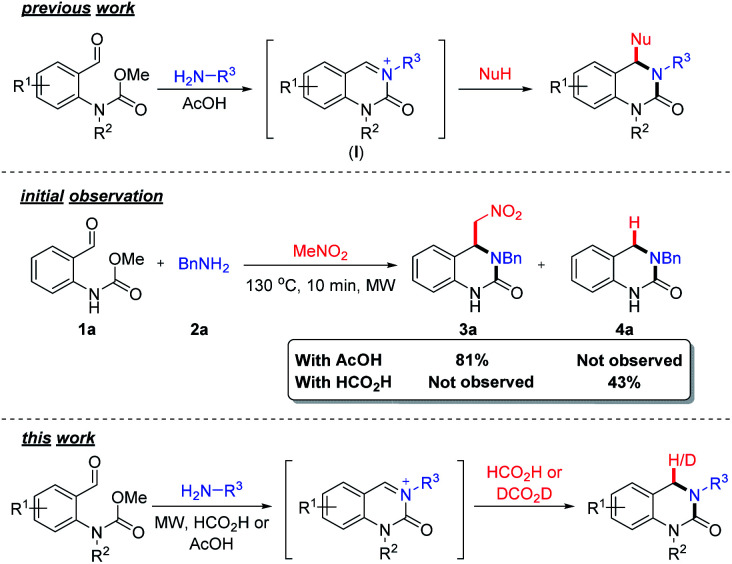
Overview of previous work on the formation of substituted DHQs *via* carbamate induced cyclization cascade. Initial observation using formic acid and an overview of this work.

The 3,4-dihydroquinazolinone (DHQ) moiety is a privileged scaffold in medicinal chemistry, as such compounds containing this unit have been reported to exhibit biological activity against a wide range of therapeutic targets. The most significant compound in this class, in terms of clinical utility, is the calcitonin gene-related peptide receptor antagonist olcegepant (Fig. 1), which demonstrated efficacy and safety in phase II trials as an anti-migraine agent.^1,2^ In addition, DHQs with potent anti-HIV,^3^ anti-psychotic,^4,5^ anti-cancer^6,7^ and anti-microbial^8^ activities as well as potential for the treatment of cardiovascular^9^ and anti-inflammatory disorders^10^ have been disclosed. Recently PFI-1, a potent and selective inhibitor of the bromo- and extra C-terminal domain (BET) family of bromodomains was developed using a fragment based approach starting from small DHQ-containing hit.^11,12^ As consequence of their pervading biological importance, numerous synthetic approaches to access this ring system are available. These include the cyclization of *o*-acyl/*o*-aminoanilines^13–17^ or *o*-nitrobenzylamines^18^ with a carbonyl donor or the nucleophilic annulation of *o*-functionalized aniline derivatives^19^ in addition to more recent methodologies relying on the use of expensive transition metal catalysts^20–24^ or toxic selenium and carbon monoxide.^25^ In 2015, we disclosed a novel multicomponent strategy to assemble diversely substituted DHQs *via* an *N*-acyliminium ion cyclization cascade.^26^ This is a simple, highly attractive approach for accessing novel and densely substituted DHQ analogues, based on an array of different chemistries (Scheme 1).^27–31^

During the course of our investigations on the reactivity of *N*-acyliminium ions (I), we observed the formation of an unknown side-product (4a) when formic acid was used as a solvent, in an aza-Henry based cyclization cascade, *in lieu* of acetic acid (Scheme 1). Subsequent characterization studies revealed that this compound retained the DHQ core but lacked the expected C4-substituent. The most plausible explanation for the formation of 4a is the *in situ* reduction of the iminium ion intermediate and indeed, the catalyst-free formic acid (or derivative) mediated reduction of iminium ions was first reported over a century ago^32,33^ and is known as the Leuckart–Wallach reaction.

Although the use of formic acid as a green and renewable reductant in transfer hydrogen chemistry^34^ has received significant attention, there are surprisingly few examples utilizing the Leuckart–Wallach reaction manifold in the literature.^35–43^ Realizing that this would provide an efficient, sustainable and straightforward entry point into DHQ scaffolds structurally similar to olcegepant and PFI-1, we set about further exploring the formation of 4a. Herein, we describe the discovery and development of novel metal-free Leuckart–Wallach type reductive cyclization cascade of *o*-formyl methylcarbamates for the preparation of biologically relevant DHQs.

## Results and discussion

Our study commenced with a survey of reaction conditions with the aim of increasing the yield of DHQ 4a (Table 1). Simple removal of nitromethane led to a marked increase in yield (64%, entry 1) most likely by suppression of competing Henry-type side reactions. An increase in the reaction temperature and time (150 °C and 30 min) afforded full consumption of 1a and the desired product was isolated in 77% yield (entry 2). Although the amount of amine 2a could be reduced to 1.5 equiv. without affecting the yield (entry 4) further decreases were found to be detrimental to the reaction outcome (entry 5). In cases where a larger excess (>1.5 equiv.) of 2a was used, purification was troublesome due to the formation of benzylformamide as a side-product and in entry 5 unreacted starting material 1a was observed (LCMS analysis). Finally, the reaction time was probed with shorter times leading to the presence of unreacted 1a and *N*-acyliminium ion I and extending the reaction time afforded a lower yield (entry 6). Accordingly, the conditions from entry 4 were chosen for further evaluation.

**Table 1:** Optimization of the reaction conditions^a^

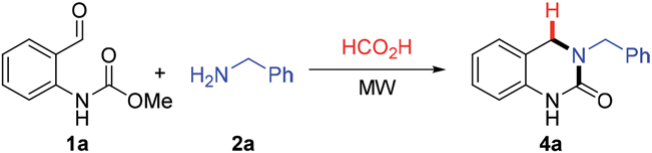				
Entry	2a (equiv.)	Temp. (°C)	Time (min)	Yield^b^ (%)
1	2.0	130	10	64
2	2.0	150	30	77
3	1.7	150	30	75
**4**	**1.5**	**150**	**30**	**83**
5	1.3	150	30	76
6	1.5	150	50	67

a Reaction conditions: 1 equiv. aldehyde 1a (0.28 mmol scale), 1 mL HCO_2_H.

b Isolated yields.

With the optimized reaction conditions in hand, the substrate scope was evaluated using various *o*-formyl methylcarbamate and benzyl amine derivatives (Table 2). In general, the reaction was compatible with a wide range of substrates, affording moderate to excellent yields of the desired DHQ products. Carbamates bearing electron donating (4b, c) or electron withdrawing (4d–h) substituents were well tolerated with the latter returning slightly lower yields. The presence of an *o*-substituent (4c) or an *N*-benzyl group (4i) was also compatible with the reaction indicating a tolerance towards steric bulk around the carbamate center. Similarly, the amine nucleophile scope was found to be broad with sterically and electronically diverse substrates all delivering the target DHQ products in good to excellent yields. Notably, the reaction was also successfully extended to heterocyclic amines to afford the thiophene and pyridine derivatives 4n and 4o, respectively. Finally, to investigate the potential for scalability, the reaction was performed on a 2 mmol scale resulting in an 86% yield of 4a.

**Table 2:** Substrate scope using various *o*-formyl methylcarbamates and benzyl amines^a^

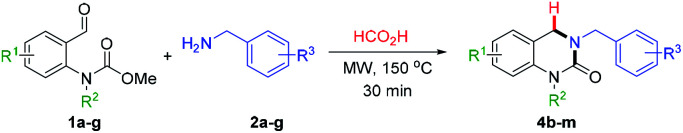
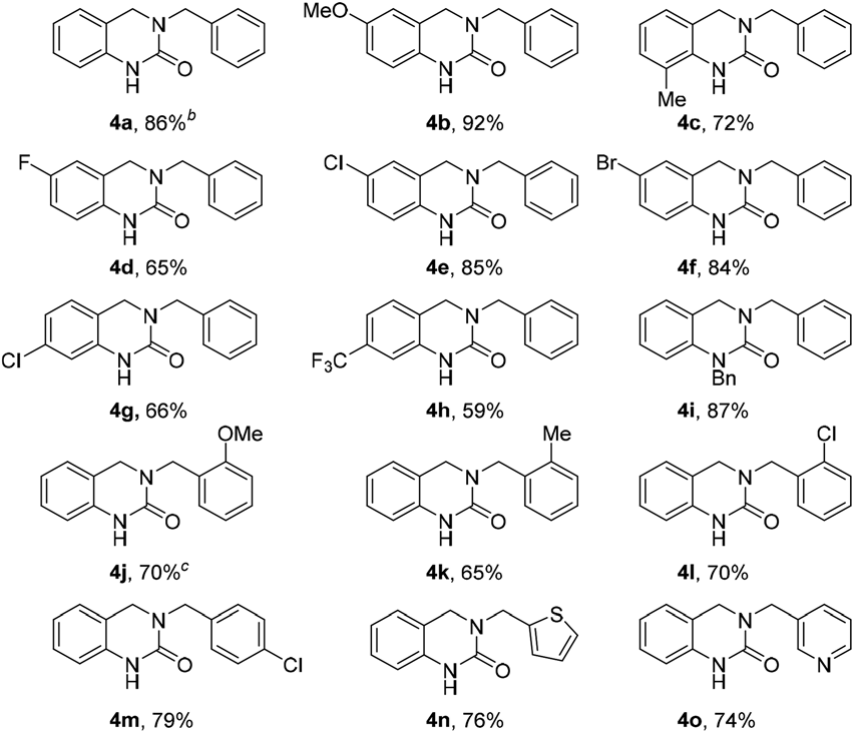

a Isolated yield (unless otherwise stated). Reactions were performed with 1 equiv. aldehyde (0.28 mmol scale), 1.5 equiv. benzylamine in HCO_2_H (1 mL).

b Reaction conducted on 2 mmol scale.

c Heated at 120 °C.

To further expand the reaction scope, we sought to explore the use of aliphatic amine nucleophiles *in lieu* of substituted benzyl amines. However, during our initial scouting reactions we noted a marked difference in reactivity, with only low levels of conversion observed (LCMS analysis) even after prolonged heating times. We reasoned that this could be the result of the higher basicity of these substrates, leading to an increase in the proportion of unreactive ammonium cations, under the acidic reaction conditions. To overcome this issue, we investigated a telescoped one-pot protocol where the key *N*-acyliminium ion intermediate was first generated using the less acidic acetic acid^26^ and subsequently reduced through the addition of formic acid. As shown in Table 3, the reaction performed well with wide range of amine nucleophiles to afford the corresponding DHQ products 5a–5m in up to 92% yield. Pleasingly, linear, branched and cyclic primary amines were all found to be suitable substrates and the use of NH_4_OAc was also successful. When ethanolamine was employed as the nucleophile, the corresponding formate ester 5j was isolated in 81% yield and this was readily hydrolyzed to afford alcohol 5k in an overall yield of 74%. The reaction scope could even be extended to acid-sensitive and less-reactive nucleophiles to afford moderate yields of the corresponding products 5l and 5m, respectively. The synthesis of 5l is particularly noteworthy given the labile nature of the Boc group and the elevated temperature and acidic reaction conditions.

**Table 3:** Substrate scope with aliphatic and aromatic amines^a^

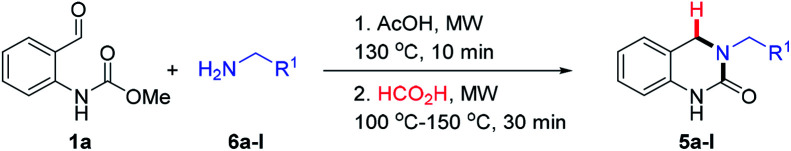
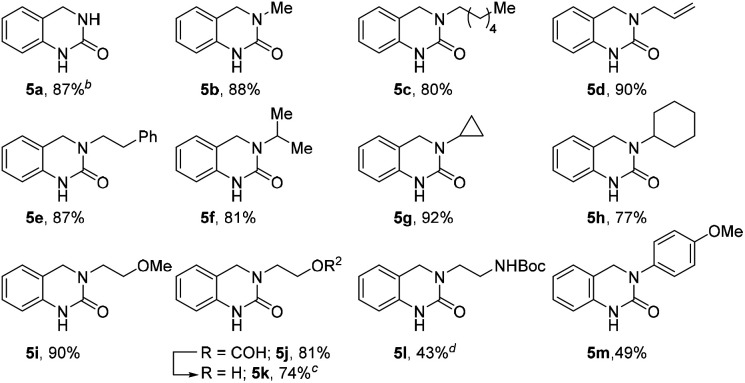

a Isolated yield. Unless otherwise stated, reactions were performed with 1 equiv. aldehyde (0.28 mmol scale), 1.5 equiv. amine in AcOH (1 mL) and HCO_2_H (1 mL).

b Heated at 130 °C in step 2.

c Treated with NaOAc (10 equiv.) in EtOH at reflux for 5 h.

d Heated at 100 °C with 10 equiv. HCO_2_H for 30 min in step 2.

**Scheme 2:**
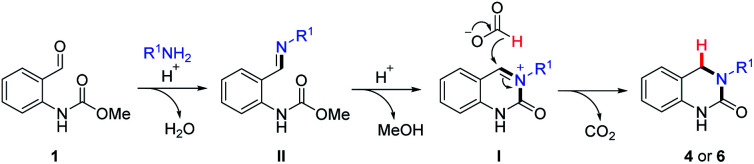
Proposed reaction pathway.

**Scheme 3:**
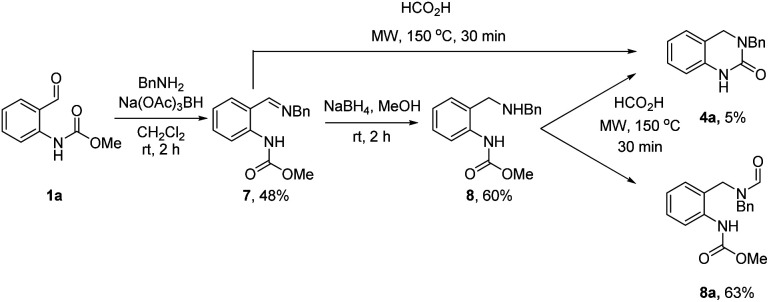
Control experiments probing possible reaction intermediates.

Based on the above results and our previous studies,^26^ the reaction is believed to occur *via* a cascade imine/iminium ion/Leuckart–Wallach type reaction process (Scheme 2). The reaction begins with the acid-mediated formation of imine II followed by annulation onto the pendant carbamate resulting in the formation of cyclic *N*-acyl iminium ion I. Finally, reduction of this highly electrophilic intermediate by formic acid leads to the target DHQ products 4 and 6, in an effective and efficient process, generating H_2_O, CO_2_ and MeOH as the only reaction byproducts. This is the most likely set of events in the two-step reaction (Table 3), as complete conversion to I was routinely observed (LCMS analysis) prior to the addition of formic acid. However, when the reaction is conducted solely in formic acid (Tables 1 and 2) an alternative scenario where reduction of II occurs prior to cyclization is also plausible. To further investigate this possibility, we set about synthesizing an imine of type-II and its corresponding reduced form (Scheme 3) to assess their relevance in our reaction system.

To this end, *o*-formyl carbamate (1a) and benzylamine (2a) were first reacted under standard reductive amination conditions using Na(OAc)_3_BH (Scheme 3). Surprisingly, formation of amine 8 was not observed (LCMS analysis) and instead imine 7 was isolated in 48% yield. Subsequent treatment of 7 with the more reactive NaBH_4_ led to formation of the desired amine product 8. We next subjected 7 and 8 to the optimized reaction conditions from Table 2. Interestingly, full conversion of 7 to 4a was observed (LCMS analysis) whereas the reaction with 8 gave the *N*-formyl derivative 8a as the major product (63%) and 4a was isolated in only 5% yield. These results strongly support the intermediacy of a cyclic *N*-acyl iminium ion species under both sets of reaction conditions.

**Scheme 4:**
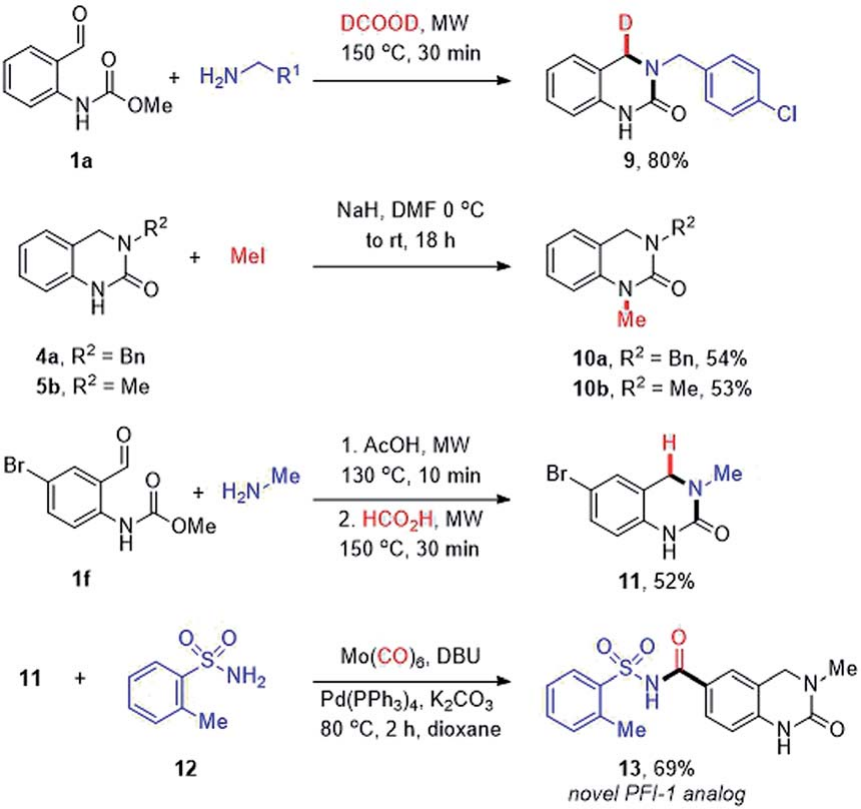
Synthetic utility of the cascade DHQ methodology.

Finally, the utility of our methodology was demonstrated through the synthesis of additional, more elaborate DHQ derivatives (Scheme 4). Firstly, we were intrigued by the possibility of using deuterated formic acid to potentially enable the site-selective introduction of a deuterium atom. The H/D isotopic replacement is an important tool to modulate the PK/PD properties of drug candidates^44^ and provides a potential handle for mechanistic studies. Thus, 1a was reacted with 4-chlorobenzylamine (2m) using formic acid-*d*_2_ to afford the mono deuterated DHQ compound 9 in 80% yield. The site-selective incorporation of the deuterium atom at the benzylic position was confirmed by ^1^H and ^13^C NMR analysis. Next, selective *N*1-methylation was conducted using NaH and methyl iodide to afford moderate yields of 10a and 10b. Lastly, a novel analog of the BET bromodomain inhibitor PFI-1 (13) was synthesized *via* an efficient two-step route starting with the cascade cyclization of 1f and methylamine (4l), followed by a palladium-catalyzed aminocarbonylation with *o*-toluenesulfonamide (12).

## Conclusions

In summary, we have developed a straightforward and high-yielding protocol for the synthesis of 3,4-dihydroquinazolinone by a novel imine/cyclization/Leuckart–Wallach type cascade process. Notably, the reaction is metal-free and requires only the addition of formic acid as a dual Brønsted acid/reductant or a combination of acetic acid and formic acid for aliphatic amine substrates. Moreover, the only byproducts formed during the reaction are H_2_O, MeOH and CO_2_ making the overall process highly efficient and sustainable. Mechanistic studies supported the formation of a cyclic *N*-acyl iminium ion intermediate prior to reduction by formic acid. Finally, the methodology was exemplified on a range of different substrates and was even extended to deuterium incorporation and synthesis of a novel analog of the BET bromodomain inhibitor PFI-1. We hope that this work will encourage others to explore the underutilized Leuckart–Wallach reaction as a green synthetic manifold to prepare biologically important compounds.

## Conflicts of interest

There are no conflicts to declare.

## Supplementary Material

RA-011-D0RA10142G-s001

## References

[cit1] Recober A., Russo A. F. (2007). IDrugs.

[cit2] Bell I. M. (2014). J. Med. Chem..

[cit3] Corbett J. W., Ko S. S., Rodgers J. D., Gearhart L. A., Magnus N. A., Bacheler L. T., Diamond S., Jeffrey S., Klabe R. M., Cordova B. C., Garber S., Logue K., Trainor G. L., Anderson P. S., Erickson-Viitanen S. K. (2000). J. Med. Chem..

[cit4] Uruno Y., Konishi Y., Suwa A., Takai K., Tojo K., Nakako T., Sakai M., Enomoto T., Matsuda H., Kitamura A., Sumiyoshi T. (2015). Bioorg. Med. Chem. Lett..

[cit5] Sugiyama Y. , Hazama M. and Iwakami N., U.S. Pat., US 9,199,969 B, 2005

[cit6] Freyne E. J. E. , Mevellec L. A., Vialard J. E., Meyer C., Pasquier E. T. J., Bourdrez X. M. and Angibaud P. R., WO2009118384, 2009

[cit7] Dohle W., Jourdan F. L., Menchon G., Prota A. E., Foster P. A., Mannion P., Hamel E., Thomas M. P., Kasprzyk P. G., Ferrandis E., Steinmetz M. O., Leese M. P., Potter B. V. L. (2018). J. Med. Chem..

[cit8] Tiwari A. K., Mishra A. K., Bajpai A., Mishra P., Sharma R. K., Pandey V. K., Singh V. K. (2006). Bioorg. Med. Chem. Lett..

[cit9] Barrett J. A., Woltmann R. F., Swillo R. S., Kasiewski C., Faith W. C., Campbell H. F., Perrone M. H. (1990). J. Cardiovasc. Pharmacol..

[cit10] Stelmach J. E., Liu L., Patel S. B., Pivnichny J. V., Scapin G., Singh S., Hop C. E. C. A., Wang Z., Strauss J. R., Cameron P. M., Nichols E. A., O'Keefe S. J., O'Neill E. A., Schmatz D. M., Schwartz C. D., Thompson C. M., Zaller D. M., Doherty J. B. (2003). Bioorg. Med. Chem. Lett..

[cit11] Picaud S., Da Costa D., Thanasopoulou A., Filippakopoulos P., Fish P. V., Philpott M., Fedorov O., Brennan P., Bunnage M. E., Owen D. R., Bradner J. E., Taniere P., O'Sullivan B., Müller S., Schwaller J., Stankovic T., Knapp S. (2013). Cancer Res..

[cit12] Fish P. V., Filippakopoulos P., Bish G., Brennan P. E., Bunnage M. E., Cook A. S., Federov O., Gerstenberger B. S., Jones H., Knapp S., Marsden B., Nocka K., Owen D. R., Philpott M., Picaud S., Primiano M. J., Ralph M. J., Sciammetta N., Trzupek J. D. (2012). J. Med. Chem..

[cit13] Barrow J. C., Rittle K. E., Reger T. S., Yang Z. Q., Bondiskey P., McGaughey G. B., Bock M. G., Hartman G. D., Tang C., Ballard J., Kuo Y., Prueksaritanont T., Nuss C. E., Doran S. M., Fox S. V., Garson S. L., Kraus R. L., Li Y., Marino M. J., Kuzmick Graufelds V., Uebele V. N., Renger J. J. (2010). ACS Med. Chem. Lett..

[cit14] Takai H., Obase H., Teranishi M., Karasawa A., Kubo K., Shuto K., Kasuya Y., Shigenobu K. (1986). Chem. Pharm. Bull..

[cit15] Seitz W., Geneste H., Backfisch G., Delzer J., Graef C., Hornberger W., Kling A., Subkowski T., Zimmermann N. (2008). Bioorg. Med. Chem. Lett..

[cit16] Camacho M. E., Chayah M., García M. E., Fernández-Sáez N., Arias F., Gallo M. A., Carrión M. D. (2016). Arch. Pharm..

[cit17] Wang M., Han J., Si X., Hu Y., Zhu J., Sun X. (2018). Tetrahedron Lett..

[cit18] Shi D., Dou G., Li Z.-Y. (2007). J. Chem. Res..

[cit19] Bergman J., Brynolf A., Elman B., Vuorinen E. (1986). Tetrahedron.

[cit20] Ferraccioli R., Carenzi D. (2003). Synthesis.

[cit21] Willwacher J., Rakshit S., Glorius F. (2011). Org. Biomol. Chem..

[cit22] Saunthwal R. K., Patel M., Danodia A. K., Verma A. K. (2015). Org. Biomol. Chem..

[cit23] Frost G. B., Mittelstaedt M. N., Douglas C. J. (2019). Chem.–Eur. J..

[cit24] Wang Q., An J., Alper H., Xiao W. J., Beauchemin A. M. (2017). Chem. Commun..

[cit25] Zhou R., Qi X., Wu X.-F. (2019). ACS Comb. Sci..

[cit26] Stevens M. Y., Wieckowski K., Wu P., Sawant R. T., Odell L. R. (2015). Org. Biomol. Chem..

[cit27] Sawant R. T., Stevens M. Y., Odell L. R. (2015). Molbank.

[cit28] Sawant R. T., Stevens M. Y., Sköld C., Odell L. R. (2016). Org. Lett..

[cit29] Sawant R. T., Stevens M. Y., Odell L. R. (2017). Chem. Commun..

[cit30] Ramana D. V., Vinayak B., Dileepkumar V., Murty U. S. N., Chowhan L. R., Chandrasekharam M. (2016). RSC Adv..

[cit31] Sawant R. T., Stevens M. Y., Odell L. R. (2018). ACS Omega.

[cit32] Leuckart R. (1885). Ber. Dtsch. Chem. Ges..

[cit33] Wallach O. (1891). Ber. Dtsch. Chem. Ges..

[cit34] Supronowicz W., Ignatyev I. A., Lolli G., Wolf A., Zhao L., Mleczko L. (2015). Green Chem..

[cit35] Loupy A., Monteux D., Petit A., Aizpurua J. M., Domínguez E., Palomo C. (1996). Tetrahedron Lett..

[cit36] Lee S. C., Park S. B. (2007). Chem. Commun..

[cit37] O'Connor D., Lauria A., Bondi S. P., Saba S. (2011). Tetrahedron Lett..

[cit38] Barba F., Recio J., Batanero B. (2013). Tetrahedron Lett..

[cit39] Wei Y., Wang C., Jiang X., Xue D., Liu Z. T., Xiao J. (2014). Green Chem..

[cit40] Frederick M. O., Kjell D. P. (2015). Tetrahedron Lett..

[cit41] Frederick M. O., Pietz M. A., Kjell D. P., Richey R. N., Tharp G. A., Touge T., Yokoyama N., Kida M., Matsuo T. (2017). Org. Process Res. Dev..

[cit42] Skachilova S. Y., Zheltukhin N. K., Sergeev V. N., Davydova N. K. (2018). Pharm. Chem. J..

[cit43] De A., Ghosal N. C., Mahato S., Santra S., Zyryanov G. V., Majee A. (2018). ChemistrySelect.

[cit44] Gant T. G. (2014). J. Med. Chem..

