# Supplementary material for: Synthesis of substituted 3,4-dihydroquinazolinones via a metal free Leuckart–Wallach type reaction
Source: RSC Adv. 2020 Dec 23;11(1):349–53. doi: 10.1039/d0ra10142g (PMC8691039; doi:10.1039/d0ra10142g)
Supplement: RA-011-D0RA10142G-s001 [file RA-011-D0RA10142G-s001.pdf]

Electronic Supplementary Material (ESI) for RSC Advances.
This journal is © The Royal Society of Chemistry 2020

Supplementary information for

Synthesis of Substituted 3, 4-Dihydroquinazolinones via a Metal Free Leuckart-Wallach Type Reaction

Suvarna Bokale-Shivale,[a] Mohammad A. Amin,[a] Rajiv T. Sawant,[a] Marc Y. Stevens,[a] Lewend Turanli,[a] Adam Hallberg,[a] Suresh B. Waghmode[b] and Luke R .Odell[a*]

[a]*Department of Medicinal Chemistry, Uppsala Biomedical Center, Uppsala University, P. O. Box 574, SE-751 23 Uppsala, Sweden. E-mail: luke.odell@ilk.uu.se*

[b]*Department of Chemistry, Savitribai Phule Pune University (formerly Pune University), Ganeshkhind, Pune 411 007.*

Experimental section

General Details	:	S2
General Procedures	:	S3
Characterisation of compounds	:	S4 - S15
NMR Spectra	:	S16 – S52
References	:	S53

General Details

All reagents were purchased at the highest commercial quality and used without further purification unless otherwise stated. Solvents used for extraction and silica gel chromatography (EtOAc, hexane, *n*-pentane, dichloromethane and methanol) were used without purification or removal of water. Yields are for isolated, homogenous and spectroscopically pure material, unless otherwise stated. Reaction progress was monitored using thin layer chromatography (0.25 mm E. Merck silica plates, 60F-254, visualized with 254 nm UV light). Silica gel chromatography was carried out using E. Merck silica gel (60 Å pore size, particle size 40-63 nm). ^{1}H NMR spectra were recorded at 400 MHz and ^{13}C NMR spectra at 100 MHz. The chemical shifts for ^{1}H NMR and ^{13}C NMR were referenced to TMS via residual solvent signals (^{1}H, $CDCl_3$ at 7.26 ppm; ^{13}C, CDCl3 at 77.16 ppm; ^{1}H, DMSO-d_6 at 2.45 ppm; ^{13}C, DMSO-d_6 at 39.43 ppm). Microwave reactions were performed in an Initiator single mode reactor producing controlled irradiation at 2450 MHz and the temperature was monitored using the built-in online IR sensor. LC/MS was performed on an instrument equipped with a CP-Sil 8 CB capillary column (50 x 3.0 mm, particle size 2.6 µm, pore size 100 Å) operating at an ionization potential of 70 eV using a CH_3CN/H_2O gradient (0.05% HCO_2H). Accurate mass values were determined using an electrospray ionization source with a 7-T hybrid ion trap and a TOF detector or by chemical ionization using ammonia as carrier gas. Unless otherwise stated, all reactions were performed on a 0.28 mmol scale in sealed Pyrex microwave-transparent process vials designed for 0.5–2 mL reaction volumes.

General procedure for the synthesis of 3,4-dihydroquinazolinones.

Preparation of starting materials.

Compounds **1a**–**1g** are known and were prepared from the corresponding amino alcohols following literature procedures.[1,2]

Procedure A (one pot-one step): A 0.5–2 mL Pyrex process vial was charged with aldehyde **1** (50.0 mg, 0.28 mmol), amine (1.5 equiv.) and HCO_2H (1 mL). The vial was sealed and subjected to microwave irradiation at 150 °C for 30 min. After cooling to 30–40 °C, the reaction mixture was concentrated *in vacuo* and purified by silica gel chromatography.

Procedure B (one pot-two step): A 0.5–2 mL Pyrex process vial was charged with aldehyde **1** (50.0 mg, 0.28 mmol), amine (1.5 equiv.) and AcOH (1 mL). The vial was sealed and subjected to microwave irradiation at 130 °C (unless otherwise stated, temp. change) for 10 min (step 1). After cooling to 30–40 °C, HCO_2H (1 mL) was added through the septum with a syringe and the reaction mixture was heated at 150 °C (unless otherwise stated, temp. change) for further 30 min (step 2). The reaction mixture was concentrated *in vacuo* and purified by silica gel chromatography.

***3-Benzyl-3,4-dihydroquinazolin-2(1H)-one*[3] (4a):** Following procedure A. White solid (43 mg, 83%). Following procedure A on 2 mmol scale, white solid (410 mg, 86%). Eluted with 10% MeOH in $CHCl_3$ (R_f = 0.45). ^1H NMR (400 MHz, DMSO-d_6) δ 9.31 (s, 1H), 7.39–7.24 (m, 5H), 7.16–7.08 (m, 1H), 7.06–6.98 (m, 1H), 6.87–6.76 (m, 2H), 4.54 (s, 2H), 4.30 (s, 2H); ^{13}C NMR (100 MHz, DMSO-d_6) δ 153.7, 137.6, 137.5, 128.6, 127.9, 127.7, 127.3, 125.6, 121.1, 117.6, 113.4, 49.3, 47.5, 39.7. MS (ESI): Calc´d for $C_{15}H_{15}N_2O$ $[M + H]^+$ *m/z* 239.1184, found 239.1186.

***3-Benzyl-6-methoxy-3,4-dihydroquinazolin-2(1H)-one* (4b):** Following procedure A. Pale yellow solid (70 mg, 92%). Eluted with 50% EtOAc in petroleum ether (R_f = 0.25). ^1H NMR (400 MHz, $CDCl_3$) δ 7.88 (s, 1H), 7.37–7.28 (m, 5H), 6.74–6.65 (m, 2H), 6.50 (dd, *J* = 2.5, 0.8 Hz, 1H), 4.67 (s, 2H), 4.32 (s, 2H), 3.72 (s, 3H); ^{13}C NMR (100 MHz, $CDCl_3$) δ 161.1, 155.1, 154.8, 136.8, 130.4, 128.8, 128.2, 128.0, 127.7, 118.5, 114.8, 113.9, 111.2, 55.8, 50.6, 48.4. MS (ESI): Calc´d for $C_{16}H_{17}N_2O_2$ $[M + H]^+$ *m/z* 269.1290, found 269.1295.

***3-Benzyl-8-methyl-3,4-dihydroquinazolin-2(1H)-one* (4c):** Following procedure A. White solid (44 mg, 72 %). Eluted with 15% EtOAc in *n*-pentane (R_f = 0.28). ^1H NMR (400 MHz, $CDCl_3$) δ 7.40–7.28 (m, 6H), 7.02–6.99 (m, 1H), 6.86–6.79 (m, 2H), 4.68 (s, 2H), 4.34 (s, 2H), 2.25 (s, 3H). ^{13}C NMR (100 MHz, $CDCl_3$) δ 154.3, 136.7, 135.1, 129.5, 128.8, 128.0, 127.5, 123.3, 121.5, 121.4, 117.2, 50.4, 48.1, 16.6. MS (ESI): Calc´d for $C_{16}H_{17}N_2O$ $[M + H]^+$ *m/z* 253.1349, found *m/z* 253.1341.

***3-Benzyl-6-fluoro-3,4-dihydroquinazolin-2(1H)-one* (4d):** Following procedure A. White solid (41 mg, 65 %). Eluted with 10% EtOAc in *n*-pentane (R_f = 0.35). ^1H NMR (400 MHz, $CDCl_3$) δ 8.15 (s, 1H), 7.38–7.27 (m, 5H), 6.85 (ddd, *J* = 8.6, 2.8, 0.8 Hz, 1H), 6.68 (ddd, *J* = 8.4, 8.0, 3.7 Hz, 2H), 4.67 (s, 2H), 4.31 (s, 2H). ^{13}C NMR (100 MHz, $CDCl_3$) δ 159.7 (d, *J* = 240.2 Hz), 154.5, 136.4, 133.0 (d, *J* = 2.4 Hz), 128.7, 128.0, 127.6, 118.7 (d, *J* = 7.5 Hz), 114.9

(d, *J* = 19.1 Hz), 114.7 (d, *J* = 3.5 Hz), 112.3 (d, *J* = 24.5 Hz), 50.3, 47.8. MS (ESI): Calc´d for $C_{15}H_{14}N_2OF$ [M + H]$^+$ *m/z* 257.1095, found *m/z* 257.1090.

3-Benzyl-6-chloro-3,4-dihydroquinazolin-2(1H)-one **(4e):** Following procedure A. White solid (65 mg, 85%). Eluted with 10% MeOH in $CHCl_3$ (R_f = 0.50). ^{1}H NMR (400 MHz, DMSO-d_6) δ 9.46 (s, 1H), 7.41–7.22 (m, 5H), 7.21–7.09 (m, 2H), 6.80 (d, *J* = 8.4 Hz, 1H), 4.53 (s, 2H), 4.30 (s, 2H); ^{13}C NMR (101 MHz, DMSO-d_6) δ 153.4, 137.2, 136.7, 128.6, 127.7, 127.6, 127.3, 125.5, 124.6, 119.7, 114.9, 49.3, 47.1. MS (ESI): Calc´d for $C_{15}H_{14}N_2OCl$ [M + H]$^+$ *m/z* 273.0795, found 273.0801.

3-Benzyl-6-bromo-3,4-dihydroquinazolin-2(1H)-one **(4f):** Following procedure A. White solid (75 mg, 84%). Eluted with 10% MeOH in $CHCl_3$ (R_f = 0.40). ^{1}H NMR (400 MHz, DMSO-d_6) δ 9.46 (s, 1H), 7.40–7.22 (m, 7H), 6.75 (d, *J* = 8.4 Hz, 1H), 4.53 (s, 2H), 4.31 (s, 2H); ^{13}C NMR (100 MHz, DMSO-d_6) δ 153.3, 137.2, 137.1, 130.6, 128.6, 128.3, 127.6, 127.3, 120.2, 115.4, 112.3, 49.3, 47.0. MS (ESI): Calc´d for $C_{15}H_{14}BrN_2O$ [M + H]$^+$ *m/z* 317.0289, found 317.0301.

3-Benzyl-7-chloro-3,4-dihydroquinazolin-2(1H)-one **(4g):** Following procedure A. White solid (37 mg, 66%). Eluted with 10% EtOAc in *n*-pentane (R_f = 0.29). ^{1}H NMR (400 MHz, DMSO-d_6) δ 9.45 (s, 1H), 7.36–7.23 (m, 5H), 7.05 (d, *J* = 8.1 Hz, 1H), 6.86 (dd, *J* = 8.1, 2.1 Hz, 1H), 6.81 (d, *J* = 2.1 Hz, 1H), 4.51 (s, 2H), 4.28 (s, 2H). ^{13}C NMR (100 MHz, DMSO-d_6) δ 153.5, 139.5, 137.6, 132.4, 129.0, 128.0, 127.8, 127.7, 121.1, 117.0, 113.1, 49.7, 47.4. MS (ESI): Calc´d for $C_{15}H_{14}N_2OCl$ [M + H]$^+$ *m/z* 273.0796, found *m/z* 273.0795.

3-Benzyl-7-(trifluoromethyl)-3,4-dihydroquinazolin-2(1H)-one **(4h):** Following procedure A. White solid (39 mg, 59 %). Eluted with 10% EtOAc in *n*-pentane (R_f = 0.33). ^{1}H NMR (400 MHz,) δ 8.02 (s, 1H), 7.40–7.27 (m, 5H), 7.15 (d, *J* = 8.0 Hz, 1H), 7.06 (d, *J* = 7.9 Hz, 1H), 6.97 (s, 1H), 4.69 (s, 2H), 4.38 (s, 2H). ^{13}C NMR (100 MHz, $CDCl_3$) δ 153.9, 137.3,

136.2, 130.8 (q, *J* = 31.5 Hz), 128.8, 128.1, 127.8, 126.2, 123.3 (q, *J* = 270.3 Hz), 121.1 (q, *J* = 1.2 Hz), 118.6 (q, *J* = 3.9 Hz), 110.4 (q, *J* = 3.8 Hz), 50.5, 47.7. MS (ESI): Calc´d for $C_{16}H_{13}N_2OF_3$ ([M +H $^+$]) *m/z* 307.1062, found *m/z* 307.1058.

1,3-Dibenzyl-3,4-dihydroquinazolin-2(1H)-one **(4i):** Following procedure A. Colorless oil (52 mg, 87%). Eluted with 10% EtOAc in *n*-pentane (R_f = 0.19). ^{1}H NMR (400 MHz, $CDCl_3$) δ 7.40–7.10 (m, 10H), 7.08 (ddd, *J* = 8.2, 7.3, 1.7 Hz, 1H), 6.96 (dd, *J* = 7.5, 1.5 Hz, 1H), 6.89 (ddd, *J* = 7.4, 7.4, 1.0 Hz, 1H), 6.72 (dd, *J* = 8.2, 1.0 Hz, 1H), 5.19 (s, 2H), 4.74 (s, 2H), 4.36 (s, 2H). ^{13}C NMR (100 MHz, $CDCl_3$) δ 155.2, 138.6, 137.9, 137.1, 128.8, 128.8, 128.3, 128.2, 127.7, 127.0, 126.5, 125.7, 122.0, 119.8, 114.1, 51.7, 47.8, 47.0. MS (ESI): Calc´d for $C_{22}H_{21}N_2O$ $[M + H]^+$ *m/z* 329.1654, found *m/z* 329.1647.

3-(2-Methoxybenzyl)-3,4-dihydroquinazolin-2(1H)-one **(4j):** Following procedure A. White solid (52 mg, 70%). Eluted with 20% EtOAc in *n*-pentane (R_f = 0.13). ^{1}H NMR (400 MHz, $CDCl_3$) δ 7.95 (s, 1H), 7.26 (dd, *J* = 7.6, 0.9 Hz, 1H), 7.20–7.16 (m, 1H), 7.09–7.02 (m, 2H), 6.91–6.77 (m, 4H), 6.65 (d, *J* = 8.5 Hz, 1H), 4.64 (s, 2H), 4.34 (s, 2H), 3.78 (s, 2H). ^{13}C NMR (100 MHz, $CDCl_3$) δ 157.6, 154.8, 137.1, 128.9, 128.5, 128.0, 125.4, 124.7, 121.7, 120.7, 117.7, 113.7, 110.3, 55.4, 48.5, 44.9. MS (ESI): Calc´d for $C_{16}H_{17}N_2O_2$ $[M + H]^+$ *m/z* 269.1290, found *m/z* 269.1281.

3-(2-Methylbenzyl)-3,4-dihydroquinazolin-2(1H)-one **(4k):** Following procedure A. White solid (46 mg, 65%). Eluted with 15% EtOAc in *n*-pentane (R_f = 0.18). ^{1}H NMR (400 MHz, $CDCl_3$) δ 7.88 (s, 1H), 7.21–7.11 (m, 5H), 6.95–6.86 (m, 2H), 6.73 (d, *J* = 7.9 Hz, 1H), 4.71 (s, 2H), 4.32 (s, 2H), 2.35 (s, 3H). ^{13}C NMR (101 MHz, $CDCl_3$) δ 154.4, 136.8, 136.9, 134.1, 130.6, 128.1, 128.1, 127.5, 126.0, 125.5, 121.8, 117.3, 113.6, 48.2, 47.8, 19.1. MS (ESI): Calc´d for $C_{16}H_{17}N_2O$ $[M + H]^+$ *m/z* 253.1341, found *m/z* 253.1337.

***3-(2-Chlorobenzyl)-3,4-dihydroquinazolin-2(1H)-one* (4l):** Following procedure A. White solid (53 mg, 70%). Eluted with 15% EtOAc in *n*-pentane (R_f = 0.18). ^{1}H NMR (400 MHz, $CDCl_3$) δ 7.96 (s, 1H), 7.43–7.37 (m, 2H), 7.25–7.19 (m, 2H), 7.16 (dd, *J* = 7.6, 1.8 Hz, 1H), 6.97 (d, *J* = 7.2 Hz, 1H), 6.91 (dd, *J* = 7.4, 0.8 Hz, 1H), 6.75 (d, *J* = 7.8 Hz, 1H), 4.83 (s, 2H), 4.43 (s, 2H). ^{13}C NMR (100 MHz, $CDCl_3$) δ 154.6, 136.8, 134.1, 133.7, 129.5, 128.9, 128.6, 128.2, 127.1, 125.5, 121.9, 117.3, 113.8, 48.5, 47.7. MS (ESI): Calc´d for $C_{15}H_{14}ClN_2O$ $[M + H]^+$ *m/z* 273.0795, found *m/z* 273.0801.

***3-(4-Chlorobenzyl)-3,4-dihydroquinazolin-2(1H)-one* (4m):** Following procedure A. White solid (60 mg, 79%). Eluted with 14% EtOAc in *n*-pentane (R_f = 0.27). ^{1}H NMR (400 MHz, $CDCl_3$) δ 7.95 (s, 1H), 7.35–7.28 (m, 4H), 7.15 (ddd, *J* = 7.8, 7.8, 1,6 Hz, 1H), 6.99–6.88 (m, 2H), 6.73 (d, *J* = 7.9 Hz, 1H), 4.64 (s, 2H), 4.33 (s, 2H). ^{13}C NMR (101 MHz, $CDCl_3$) δ 154.5, 136.7, 135.2, 133.3, 129.4, 128.8, 128.2, 125.5, 121.9, 117.2, 113.7, 49.8, 48.1. MS (ESI): Calc´d for $C_{15}H_{14}ClN_2O$ $[M + H]^+$ *m/z* 273.0795, found *m/z* 273.0802.

***3-(Thiophen-2-ylmethyl)-3,4-dihydroquinazolin-2(1H)-one* (4n):** Following procedure A. White solid (52 mg, 76%). Eluted with 2% MeOH in DCM (R_f = 0.4). ^{1}H NMR (400 MHz, $CDCl_3$) δ 8.21 (s, 1H), 7.27–7.23 (m, 1H), 7.18–7.12 (m, 1H), 7.08–7.05 (m, 1H), 7.00–6.95 (m, 2H), 6.90 (ddd, *J* = 7.5, 7.5, 1.1 Hz, 1H), 6.77 (dd, *J* = 7.9, 1.1 Hz, 1H), 4.82 (d, *J* = 0.8 Hz, 2H), 4.42 (s, 2H). ^{13}C NMR (101 MHz, $CDCl_3$) δ 154.3, 139.1, 136.7, 128.1, 126.9, 126.6, 125.6, 125.5, 121.8, 117.4, 113.8, 47.9, 45.3. MS (ESI): Calc´d for $C_{13}H_{13}N_2OS$ $[M + H]^+$ *m/z* 245.0749, found *m/z* 245.0753.

***3-(Pyridin-3-ylmethyl)-3,4-dihydroquinazolin-2(1H)-one* (4o):** Following procedure A. White solid (49 mg, 74%). Eluted with 2% MeOH in DCM (R_f = 0.16). ^{1}H NMR (400 MHz, $CDCl_3$) δ 8.50 (br s, 1H), 7.71 (d, *J* = 7.8 Hz, 1H), 7.25 (dd, *J* = 7.6, 5.0 Hz, 1H), 7.11 (td, *J* = 8.0, 1.6 Hz, 1H), 6.94–6.82 (m, 3H), 6.62 (d, *J* = 7.9 Hz, 1H), 4.62 (s, 1H), 4.30 (s, 2H). ^{13}C

NMR (101 MHz, $CDCl_3$) δ 154.6, 149.4, 149.0, 136.6, 135.9, 132.4, 128.3, 125.4, 123.7, 122.0, 116.9, 113.9, 48.2, 48.0. MS (ESI): Calc´d for $C_{14}H_{14}N_3O$ $[M + H]^+$ *m/z* 240.1137, found *m/z* 240.1138.

3,4-Dihydroquinazolin-2(1H)-one[4] **(5a):** Following procedure B. White solid (36 mg, 87%). Eluted with 25% EtOAc in *n*-pentane (R_f = 0.58). ^{1}H NMR (400 MHz, DMSO-d_6) δ 9.01 (s, 1H), 7.11 (ddd, *J* = 7.9, 7.9, 1.3 Hz, 1H), 7.09–7.04 (m, 1H), 6.85 (ddd, *J* = 7.4, 7.4 1.2 Hz, 1H), 6.78–6.74 (m, 1H), 4.30 (s, 2H). ^{13}C NMR (101 MHz, DMSO-d_6) δ 155.0, 138.5, 128.0, 126.1, 121.3, 118.5, 113.9, 42.9. MS (ESI): Calc´d for $C_8H_9N_2O$ $[M +H]^+$ *m/z* 149.0715, found *m/z* 149.0713.

3-Methyl-3,4-dihydroquinazolin-2(1H)-one[3] **(5b):** Following procedure B. $MeNH_2$ 33 wt % in absolute ethanol (0.1 mL) used as amine source. White crystalline solid (40 mg, 88%). Eluted with 2% MeOH in DCM (R_f = 0.16). ^{1}H NMR (400 MHz, $CDCl_3$) δ 8.14 (s, 1H), 7.08 (ddd, *J* = 7.6, 7.6, 1.5 Hz, 1H), 6.97–6.91 (m, 1H), 6.84 (ddd, *J* = 7.5, 7.5, 1.1 Hz, 1H), 6.68 (dd, *J* = 7.9, 1.1 Hz, 1H), 4.38 (s, 2H), 2.97 (s, 3H). ^{13}C NMR (100 MHz, $CDCl_3$) δ 154.7, 137.1, 128.1, 125.3, 121.7, 117.3, 113.8, 50.8, 34.5. MS (ESI): Calc´d for $C_9H_{11}N_2O$ $[M + H]^+$ *m/z* 163.0871, found *m/z* 163.0865.

3-Hexyl-3,4-dihydroquinazolin-2(1H)-one **(5c):** Following procedure B. White crystalline solid (37 mg, 80%). Eluted with 20% EtOAc in *n*-pentane (R_f = 0.39). ^{1}H NMR (400 MHz, $CDCl_3$) δ 7.71 (s, 1H), 7.15 (ddd, *J* = 7.7, 7.7, 1.4 Hz, 1H), 7.02 (d, *J* = 7.5 Hz, 1H), 6.91 (ddd, *J* = 7.5, 7.5, 1.0 Hz, 1H), 6.70 (d, *J* = 7.9 Hz, 1H), 4.44 (s, 2H), 3.49–3.37 (m, 2H), 1.62 (quin, *J* = 7.3 Hz, 2H), 1.40–1.25 (m, 6H), 0.88 (t, *J* = 6.8 Hz, 3H). ^{13}C NMR (101 MHz, $CDCl_3$) δ 154.4, 137.2, 128.1, 125.3, 121.6, 117.6, 113.5, 48.5, 47.1, 31.6, 26.9, 26.4, 22.5, 14.0. MS (ESI): Calc´d for $C_{14}H_{21}N_2O$ $[M + H]^+$ *m/z* 233.1654, found *m/z* 233.1654.

***3-Allyl-3,4-dihydroquinazolin-2(1H)-one* (5d):** Following procedure B. White solid (47 mg, 90%). Eluted with 20% EtOAc in *n*-pentane (R_f = 0.21). ^{1}H NMR (400 MHz, $CDCl_3$) δ 7.53 (s, 1H), 7.12–7.06 (m, 1H), 6.96 (d, *J* = 7.4 Hz, 1H), 6.86 (ddd, *J* = 7.5, 7.5 0.9 Hz, 1H), 6.64 (d, *J* = 7.9 Hz, 1H), 5.77 (ddt, *J* = 17.0, 10.1, 6.0 Hz, 1H), 5.25–5.15 (m, 2H), 4.33 (s, 2H), 4.01 (dt, *J* = 6.0, 1.3 Hz, 2H). ^{13}C NMR (101 MHz, $CDCl_3$) δ 154.3, 136.9, 132.7, 128.1, 125.5, 121.8, 117.9, 117.6, 113.6, 49.4, 47.9. MS (ESI): Calc´d for $C_{11}H_{13}N_2O$ $[M + H]^+$ *m/z* 189.1028, found *m/z* 189.1022.

***3-Phenethyl-3,4-dihydroquinazolin-2(1H)-one* (5e):** Following procedure B. White solid (37 mg, 87%). Eluted with 15% EtOAc in *n*-pentane (R_f = 0.4). ^{1}H NMR (400 MHz, $CDCl_3$) δ 8.11 (s, 1H), 7.28–7.11 (m, 5H), 7.07 (ddd, *J* = 7.9, 7.1, 1.8 Hz, 1H), 6.90–6.77 (m, 2H), 6.67 (dd, *J* = 7.9, 1.0 Hz, 1H), 4.27 (s, 2H), 3.65–3.55 (m, 2H), 2.93–2.85 (m, 2H). ^{13}C NMR (101 MHz, $CDCl_3$) δ 154.4, 139.2, 137.1, 129.0, 128.7, 128.3, 126.5, 125.6, 122.0, 117.7, 113.6, 49.5, 49.4, 33.9. MS (ESI): Calc´d for $C_{16}H_{17}N_2O$ $[M + H]^+$ *m/z* 253.1341, found *m/z* 253.1332.

***3-isoPropyl-3,4-dihydroquinazolin-2(1H)-one*[5] (5f):** Following procedure B. White solid (36 mg, 81%). Eluted with 20% EtOAc in *n*-pentane (R_f = 0.41). ^{1}H NMR (400 MHz, $CDCl_3$) δ 7.24 (s, 1H), 7.15 (ddd, *J* = 7.6, 7.6. 1.4 Hz, 1H), 7.06 (d, *J* = 7.4 Hz, 1H), 6.92 (ddd, *J* = 7.4. 7.4, 1.1 Hz, 1H), 6.74 (d, *J* = 7.6 Hz, 1H), 4.81 (hept, *J* = 6.8 Hz, 1H), 4.31 (s, 2H), 1.22 (d, *J* = 6.8 Hz, 6H). ^{13}C NMR (100 MHz, $CDCl_3$) δ 154.6, 137.2, 128.1, 125.4, 121.6, 117.9, 113.4, 44.6, 41.5, 19.1. MS (ESI): Calc´d for $C_{11}H_{15}N_2O$ $[M + H]^+$ *m/z* 191.1178, found *m/z* 191.1184.

***3-Cyclopropyl-3,4-dihydroquinazolin-2(1H)-one*[3] (5g):** Following procedure B. White solid (43 mg, 92%). Eluted with 50% EtOAc in *n*-pentane (R_f = 0.34). ^{1}H NMR (400 MHz, $CDCl_3$) δ 7.70 (s, 1H), 7.20–7.12 (m, 2H), 7.04 (d, *J* = 7.4 Hz, 1H), 6.92–6.90 (m, 1H), 6.71 (d, *J* = 7.8 Hz, 1H), 4.41 (s, 2H), 2.64 (tt, *J* = 7.0, 3.8 Hz, 1H), 0.98–0.79 (m, 2H), 0.79–0.65 (m, 2H). ^{13}C

NMR (101 MHz, $CDCl_3$) δ 156.1, 136.8, 128.1, 125.3, 121.8, 118.6, 113.5, 49.5, 29.3, 7.5. MS (ESI): Calc´d for $C_{11}H_{13}N_2O$ $[M + H]^+$ *m/z* 189.1029, found *m/z* 189.1028.

3-Cyclohexyl-3,4-dihydroquinazolin-2(1H)-one[5] **(5h):** Following procedure B. White solid (30 mg, 77%). Eluted with 12% EtOAc in *n*-pentane (R_f = 0.34). ^{1}H NMR (400 MHz, $CDCl_3$) δ 7.61 (s, 1H), 7.08 (dd, *J* = 7.7, 1.4 Hz, 1H), 6.97 (dd, *J* = 7.6, 1.4 Hz, 1H), 6.85 (ddd, *J* = 7.5, 7.5, 1.1 Hz, 1H), 6.64 (dd, *J* = 7.9, 1.1 Hz, 1H), 4.35–4.23 (m, 3H), 1.81–1.65 (m, 5H), 1.52–1.33 (m, 4H), 1.12–0.98 (m, 1H). ^{13}C NMR (101 MHz, $CDCl_3$) δ 154.5, 137.1, 128.0, 125.4, 121.6, 118.1, 113.3, 52.9, 42.8, 29.6, 25.7, 25.6. MS (ESI): Calc´d for $C_{14}H_{19}N_2O$ $[M + H]^+$ *m/z* 231.1497, found *m/z* 231.1504.

3-(2-Methoxyethyl)-3,4-dihydroquinazolin-2(1H)-one[6] **(5i):** Following procedure B. White solid (52 mg, 90%). Eluted with 30% EtOAc in pentane (R_f = 0.20). ^{1}H NMR (400 MHz, $CDCl_3$) δ 7.48 (br s, 1H), 7.18–7.10 (m, 1H), 7.05–6.98 (m, 1H), 6.92 (ddd, *J* = 7.5, 7.5, 1.0 Hz, 1H), 6.68 (ddd, *J* = 7.9, 1.4 Hz, 1H), 4.58 (s, 2H), 3.67–3.59 (m, 4H), 3.37 (s, 3H); ^{13}C NMR (100 MHz, $CDCl_3$) δ 154.6, 137.1, 128.2, 125.6, 122.0, 118.1, 113.6, 71.7, 59.1, 50.5, 47.4. MS (ESI): Calc´d for $C_{11}H_{15}N_2O_2$ $[M + H]^+$ *m/z* 207.1134, found 207.1134.

2-(2-Oxo-1,4-dihydroquinazolin-3(2H)-yl)ethyl formate **(5j):** Following procedure B. Colorless oil (50.1 mg, 81%). Eluted with 50% EtOAc in *n*-pentane (R_f = 0.50). ^{1}H NMR (400 MHz, $CDCl_3$) δ 8.30 (s, 1H), 8.01 (s, 1H) 7.13–7.06 (m, 1H), 6.95 (dd, *J* = 7.6, 1.4 Hz, 1H), 6.86 (ddd, *J* = 7.5, 7.5, 1.1 Hz, 1H), 6.68 (dd, *J* = 8.0, 1.0 Hz, 1H), 4.50 (s, 2H), 4.36 (t, *J* = 5.4 Hz, 2H), 3.68 (t, *J* = 5.4 Hz, 2H). ^{13}C NMR (101 MHz, $CDCl_3$) δ 160.8, 154.8, 136.8, 128.3, 125.3, 122.0, 117.3, 113.9, 61.8, 50.0, 46.1. Calc´d for $C_{11}H_{13}N_2O_3$ $[M + H]^+$ *m/z* 221.0926, found 221.0933.

3-(2-Hydroxyethyl)-3,4-dihydroquinazolin-2(1H)-one **(5k):** Following procedure B with the following modifications. To the crude mixture after step 2 was added EtOH (1.5 mL), NaOAc

(229 mg, 2.8 mmol) and 1 mL of water. The resulting reaction mixture was refluxed for 5 hours, cooled to room temperature and extracted with EtAOc (3 x 50 mL). The combined organic layers were dried with $MgSO_4$, concentrated *in vacuo* and purified by silica gel chromatography to afford a white solid (40 mg, 74%). Eluted with 50% EtOAc in petroleum ether (R_f = 0.10). ^{1}H NMR (400 MHz, $CDCl_3$) δ 8.00 (s, 1H), 7.15 (dd, *J* = 7.6, 7.6, 1.5 Hz, 1H), 7.01 (d, *J* = 7.5 Hz, 1H), 6.93 (ddd, *J* = 7.4, 7.4, 1.1 Hz, 1H), 6.72 (dd, *J* = 8.0, 1.1 Hz, 1H), 4.55 (s, 2H), 3.87 (q, *J* = 4.5 Hz, 2H), 3.61 (t, *J* = 4.5 Hz, 2H), 3.40 (s, 1H); ^{13}C NMR (101 MHz, $CDCl_3$) δ 156.2, 136.7, 128.5, 125.6, 122.3, 117.6, 114.0, 61.5, 50.9, 50.4. MS (ESI): Calc´d for $C_{10}H_{13}N_2O_2$ $[M + H]^+$ *m/z* 193.0977, found 193.0977.

tert-Butyl (2-(2-oxo-1,4-dihydroquinazolin-3(2H)-yl)ethyl)carbamate **(5l):** Following procedure B with the following modifications. Step 2: Formic acid (129 mg, 2.8 mmol, 10 equiv.) was added and the reaction was heated at 100 °C for 30 min. White solid (35 mg, 43%). Eluted with 40% EtOAc in iso-hexane with 0.1% formic acid (R_f = 0.40). ^{1}H NMR (400 MHz, CD_3OD) δ 7.17–7.09 (m, 1H), 7.09–7.03 (m, 1H), 6.92 (ddd, *J* = 7.5, 7.5, 1.2 Hz, 1H), 6.76 (dd, *J* = 7.8, 1.1 Hz, 1H), 4.55 (s, 2H), 3.48 (t, *J* = 6.2 Hz, 2H), 3.31–3.27 (m, 2H), 1.38 (s, 9H); ^{13}C NMR (100 MHz, CD_3OD) δ 158.5, 156.7, 138.4, 129.1, 126.5, 123.1, 119.2, 114.6, 80.1, 50.2, 47.9, 39.0, 28.7. MS (ESI): Calc´d for $C_{15}H_{22}N_3O_3$ $[M + H]^+$ *m/z* 292.1661, found 292.1664.

3-(4-Methoxyphenyl)-3,4-dihydroquinazolin-2(1H)-one[7] **(5m):** Following procedure B. White solid (35 mg, 49%). Eluted with 25% EtOAc in *n*-pentane (R_f = 0.31). ^{1}H NMR (400 MHz, $CDCl_3$) δ 7.29 (s, 1H), 7.25–7.17 (m, 2H), 7.14 (dd, *J* = 7.7, 1.4 Hz, 1H), 7.00 (d, *J* = 7.5 Hz, 1H), 6.94–6.84 (m, 3H), 6.67 (d, *J* = 7.9 Hz, 1H), 4.72 (s, 2H), 3.75 (s, 3H). ^{13}C NMR (101 MHz, $CDCl_3$) δ 157.9, 154.1, 136.8, 135.0, 128.4, 126.9, 125.4, 122.1, 118.2, 114.4, 113.6, 55.5, 52.1. MS (ESI): Calc´d for $C_{15}H_{15}N_2O_2$ $[M + H]^+$ *m/z* 255.1134, found *m/z* 255.1124.

***Methyl (E)-(2-((benzylimino)methyl)phenyl)carbamate* (7):** To a solution of benzylamine (120 mg, 1.11 mmol) and aldehyde **1a** (100 mg, 0.55 mmol) in 1,2-dichloroethane (2 mL) was added sodium triacetoxyborohydride (0.22 g, 1.11 mmol). The resulting mixture was stirred at rt under a N_2 atmosphere for 2 h and quenched by the addition of aqueous saturated $NaHCO_3$. The product was extracted with EtOAc, dried ($MgSO_4$), and the solvent removed to afford the crude product. The residue was purified by silica gel column chromatography (Eluted with 10% EtOAc in *n*-pentane (R_f = 0.68)) to afford a white solid (73 mg, 48%). ^{1}H NMR (400 MHz, $CDCl_3$) δ 12.23 (br s, 1H), 8.38–8.32 (m, 1H), 7.37–7.19 (m, 8H), 6.98 (ddd, *J* = 8.0, 8.0, 1.0 Hz, 1H), 4.77 (s, 2H), 3.69 (s, 3H). ^{13}C NMR (100 MHz, $CDCl_3$) δ 164.8, 154.7, 140.4, 138.8, 133.2, 131.6, 128.6, 127.6, 127.1, 121.4, 120.2, 118.0, 64.6, 52.1. MS (ESI): Calc´d for $C_{16}H_{17}N_2O_2$ $[M + H]^+$ *m/z* 269.1290, found *m/z* 269.1302.

***Methyl (2-((benzylamino)methyl)phenyl)carbamate* (8):** The imine **7** (50 mg, 0.18 mmol) was dissolved in methanol (0.5 mL) and cooled to 0 °C under N_2. Sodium borohydride (8.3 mg, 0.22 mmol) was added, the ice bath was removed and the mixture was stirred for a further 2 hrs. The solvent was evaporated and the crude residue was taken up in sat. $NaHCO_3$ (20 mL). The aqueous phase was extracted diluted with diethyl ether (3 x 20 mL) and the combined organic extracts were dried ($MgSO_4$), filtered and concentrated *in vacuo* to afford the crude product. The residue was purified by silica gel column chromatography (Eluted with 10% EtOAc in *n*-pentane (R_f = 0.1) to afford the title compound as white solid (30 mg, 60%). ^{1}H NMR (400 MHz, $CDCl_3$) δ 9.97 (br s, 1H), 7.93 (d, *J* = 7.4 Hz, 1H), 7.32–7.17 (m, 6H), 7.02 (dd, *J* = 7.5, 1.8 Hz, 1H), 6.89 (ddd, *J* = 7.4, 7.4, 1.1 Hz, 1H), 3.78 (s, 2H), 3.72 (s, 2H), 3.70 (s, 3H). ^{13}C NMR (100 MHz, $CDCl_3$) δ 154.4, 139.2, 138.9, 129.5, 128.5, 128.5, 128.3, 127.3, 126.2, 122.4, 119.6, 52.9, 52.3, 52.0. MS (ESI): Calc´d for $C_{16}H_{19}N_2O_2$ $[M + H]^+$ *m/z* 271.1447, found *m/z* 271.1436.

***Methyl N-[2-[[benzyl(formyl)amino]methyl]phenyl]carbamate* 8a**

Following procedure A starting from compound **8**. White solid (35 mg, 63%). Eluted with 5% MeOH in $CHCl_3$ (R_f = 0.65). ^{1}H NMR (400 MHz, $CDCl_3$) δ 8.97 (s, 1H), 8.30 (s, 1H), 8.13 (d, *J* = 8.3 Hz, 1H), 7.45–7.32 (m, 4H), 7.27 (d, *J* = 1.7 Hz, 1H), 7.26–7.24 (m, 1H), 7.05–6.95 (m, 2H), 4.35 (d, *J* = 6.5 Hz, 4H), 3.81 (s, 3H) ; ^{13}C NMR (101 MHz, $CDCl_3$) δ 163.7, 155.0, 137.8, 135.0, 131.6, 129.8, 129.3, 122.8, 121.0, 77.5, 52.5, 50.6, 42.4. MS (ESI): Calc´d for $C_{17}H_{18}N_2O_3$ $[M + Na]^+$ *m/z* 321.1210, found 321.1224.

3-(4-Chlorobenzyl)-3,4-dihydroquinazolin-2(1H)-one-4-d **(9):** Following procedure **A** using deuterated formic acid (1 mL). White solid (61 mg, 80%). Eluted with 14% EtOAc in *n*-pentane (R_f = 0.26). ^{1}H NMR (400 MHz, $CDCl_3$) δ 7.26–7.21 (m, 4H), 7.09 (dd, *J* = 7.6, 1.7 Hz, 1H), 6.92–6.81 (m, 2H), 6.63 (d, *J* = 7.9 Hz, 1H), 4.56 (s, 2H), 4.25 (s, 1H). ^{13}C NMR (101 MHz, $CDCl_3$) δ 154.2, 136.6, 135.2, 133.4, 129.4, 128.8, 128.3, 125.6, 122.0, 117.2, 113.6, 49.8, 47.7 (t, *J* = 21.0 Hz). MS (ESI): Calc´d for $C_{15}H_{13}DClN_2O$ $[M + H]^+$ *m/z* 274.0842, found *m/z* 274.0857.

3-Benzyl-1-methyl-3,4-dihydroquinazolin-2(1H)-one **(10a):** Sodium hydride (60% oil dispersion, 7 mg, 0.25 mmol) was added to a solution of 3-benzyl-3,4-dihydroquinazolin-2(1*H*)-one (**4a**, 50 mg, 0.21 mmol) in DMF (1 mL) at 0°C under nitrogen. The reaction was stirred at rt for 1 h and methyl iodide (75 mg, 0.53 mmol) was added. The reaction was stirred for 18 h, quenched with water and diluted with EtOAc. The aqueous layer was separated and extracted with ethyl acetate. The organic layer was dried ($MgSO_4$), filtered and evaporated. The residue was purified by silica gel column chromatography eluting with 25% EtOAc in *n*-pentane (R_f = 0.44). The title compound was obtained as colorless oil (28 mg, 54%). ^{1}H NMR (400 MHz, $CDCl_3$) δ 7.29–7.14 (m, 6H), 6.90–6.86 (m, 2H), 6.80 (d, *J* = 8.4 Hz, 1H), 4.60 (s, 2H), 4.19 (s,2H), 3.29 (s, 3H). ^{13}C NMR (100 MHz, $CDCl_3$) δ 155.2, 139.5, 137.0, 128.6, 128.1, 128.0, 127.4, 125.4, 121.7, 119.8, 112.7, 51.4, 47.5, 30.5. MS (ESI): Calc´d for $C_{16}H_{17}N_2O$ $[M + H]^+$ *m/z* 253.1341, found *m/z* 253.1346.

***1,3-Dimethyl-3,4-dihydroquinazolin-2(1H)-one* (10b):** Synthesized as per **10a** from 3-methyl-3,4-dihydroquinazolin-2(1*H*)-one (**5b**, 50 mg, 0.31 mmol). Purified by silica gel column chromatography eluting with 25% EtOAc in *n*-pentane (R_f = 0.35). Yellow oil (30 mg, 53%). ^{1}H NMR (400 MHz, $CDCl_3$) δ 7.18 (ddd, *J* = 8.2, 8.2, 1.6 Hz, 1H), 7.00–6.95 (m, 1H), 6.90 (ddd, *J* = 7.4, 7.4, 1.0 Hz, 1H), 6.76 (d, *J* = 8.1 Hz, 1H), 4.28 (s, 2H), 3.23 (s, 3H), 2.96 (s, 3H). ^{13}C NMR (101 MHz, $CDCl_3$) δ 155.3, 139.6, 128.2, 125.3, 121.6, 119.8, 112.6, 50.3, 35.6, 30.2. MS (ESI): Calc´d for $C_{10}H_{13}N_2O$ $[M + H]^+$ *m/z* 177.1028, found *m/z* 177.1034.

***6-Bromo-3-methyl-3,4-dihydroquinazolin-2(1H)-one*[8] (11):** Following procedure B pale yellow solid (35 mg, 52%). Eluted with 30% EtOAc in *n*-pentane (R_f = 0.20). ^{1}H NMR (400 MHz, $CDCl_3$) δ 7.72 (s, 1H), 7.30–7.23 (m, 1H), 7.16 (dd, *J* = 2.0, Hz, 1H), 6.61 (d, *J* = 8.4 Hz, 1H), 4.42 (s, 2H), 3.03 (s, 3H); ^{13}C NMR (100 MHz, $CDCl_3$) δ 154.3, 136.2, 131.3, 128.5, 119.5, 115.5, 114.1, 50.5, 34.7. MS (ESI): Calc´d for $C_{11}H_{13}N_3OBr$ $[M + CH_3CN + H]^+$ *m/z* 282.0242, found 282.0247.

***3-Methyl-2-oxo-N-(o-tolylsulfonyl)-1,2,3,4-tetrahydroquinazoline-6-carboxamide* (13):** The carbonylation reaction was conducted using a two-chamber ex-situ CO generation setup.[9] To the CO-releasing chamber (chamber A) was added $Mo(CO)_6$ (137 mg, 0.52 mmol) and 1,4-dioxane (2 mL). To the reaction chamber (chamber B) was added compound **11** (50 mg, 0.21 mmol), *ortho*-toluenesulfonamide (89 mg, 0.52 mmol), $Pd(PPh_3)_4$ (11 mg, 0.01 mmol), K_2CO_3 (72 mg, 0.52 mmol) and 1,4-dioxane (2 mL). Both chambers were sealed with gas-tight caps and DBU (126 mg, 0.83 mmol) was added to chamber A. The reaction was heated under 80 °C for 2 h, cooled to room temperature and excess CO was removed by carefully puncturing the cap. The crude reaction mixture from Chamber B was concentrated *in vacuo* and purified by silica gel column chromatography eluting with 30% EtOAc in *n*-pentane with 0.1% formic acid (R_f = 0.33). White solid (51 mg, 69%). ^{1}H NMR (400 MHz, CD_3OD) δ 8.14 (dd, *J* = 8.0, 1.4 Hz, 1H), 7.68 (dd, *J* = 8.4, 2.1 Hz, 1H), 7.58 (d, *J* = 1.9 Hz, 1H), 7.49 (dd, *J* = 7.5, 1.4 Hz, 1H),

7.40–7.34 (m, 1H), 7.30 (d, J = 7.6 Hz, 1H), 6.78 (d, J = 8.4 Hz, 1H), 4.48 (s, 2H), 2.97 (s, 1H), 2.66 (s, 1H); ^{13}C NMR (100 MHz, CD_3OD) δ 166.3, 155.0, 142.5, 138.3, 138.2, 134.2, 133.0, 131.6, 129.6, 126.9, 126.7, 125.7, 118.2, 114.2, 50.9, 34.7, 20.3. MS (ESI): Calc´d for $C_{17}H_{18}N_3O_4S$ $[M + H]^+$ *m/z* 360.1018, found 360.1022.

NMR spectra of all compounds

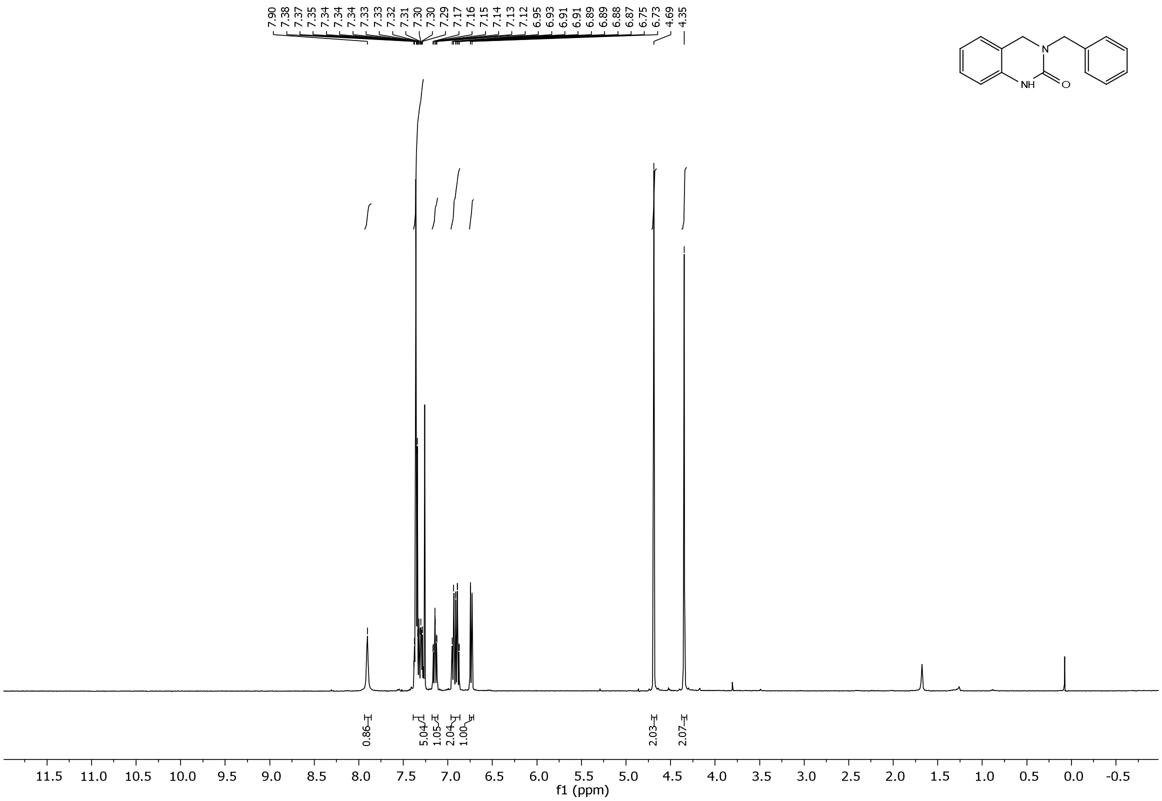

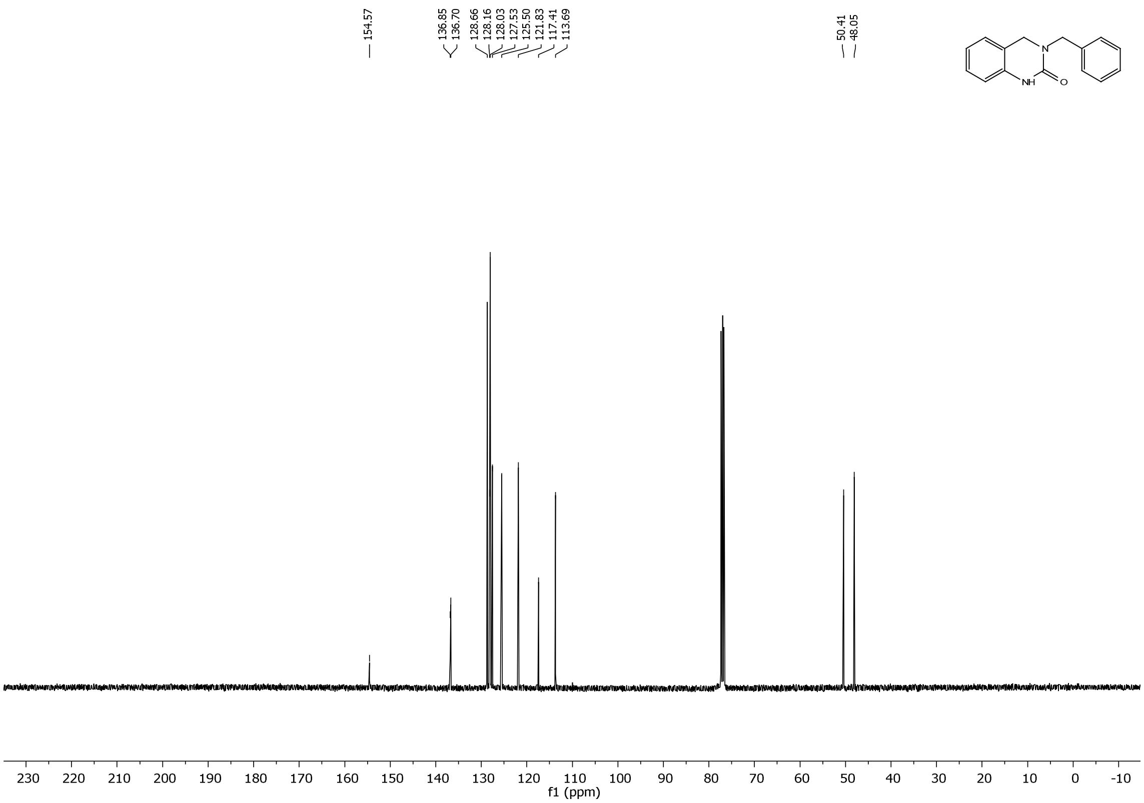

Compound 4a

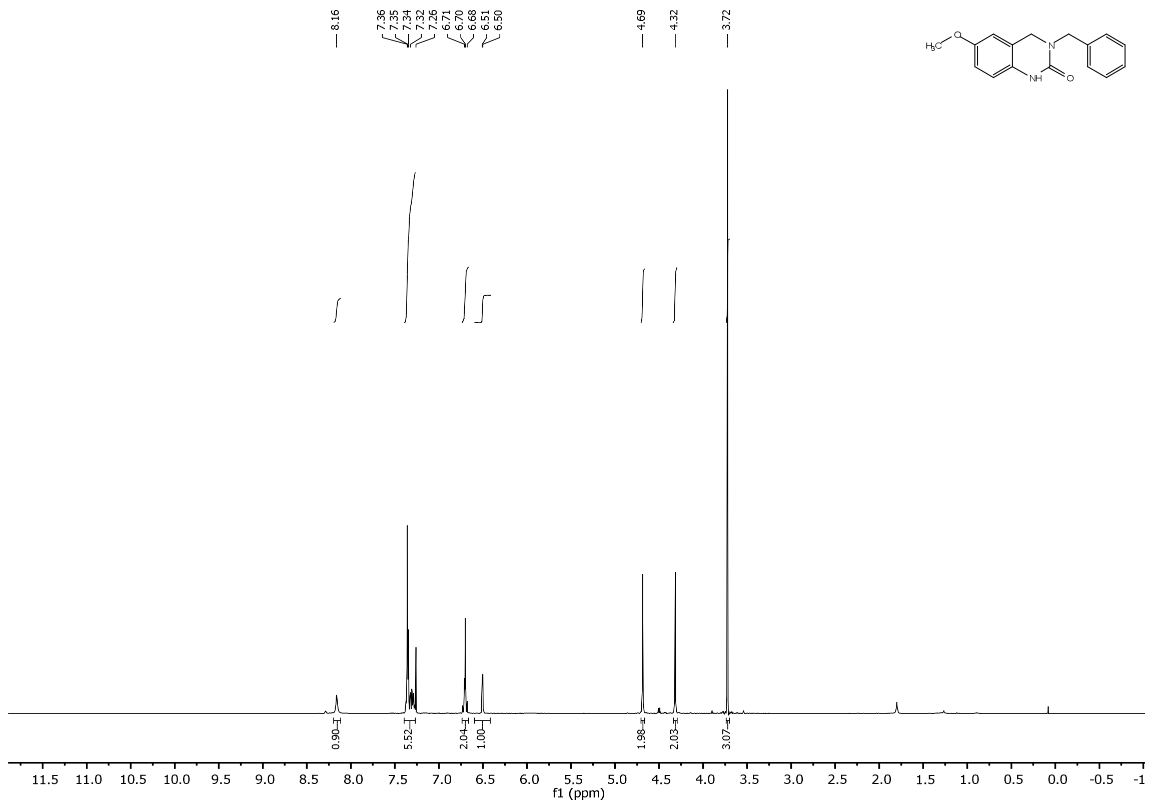

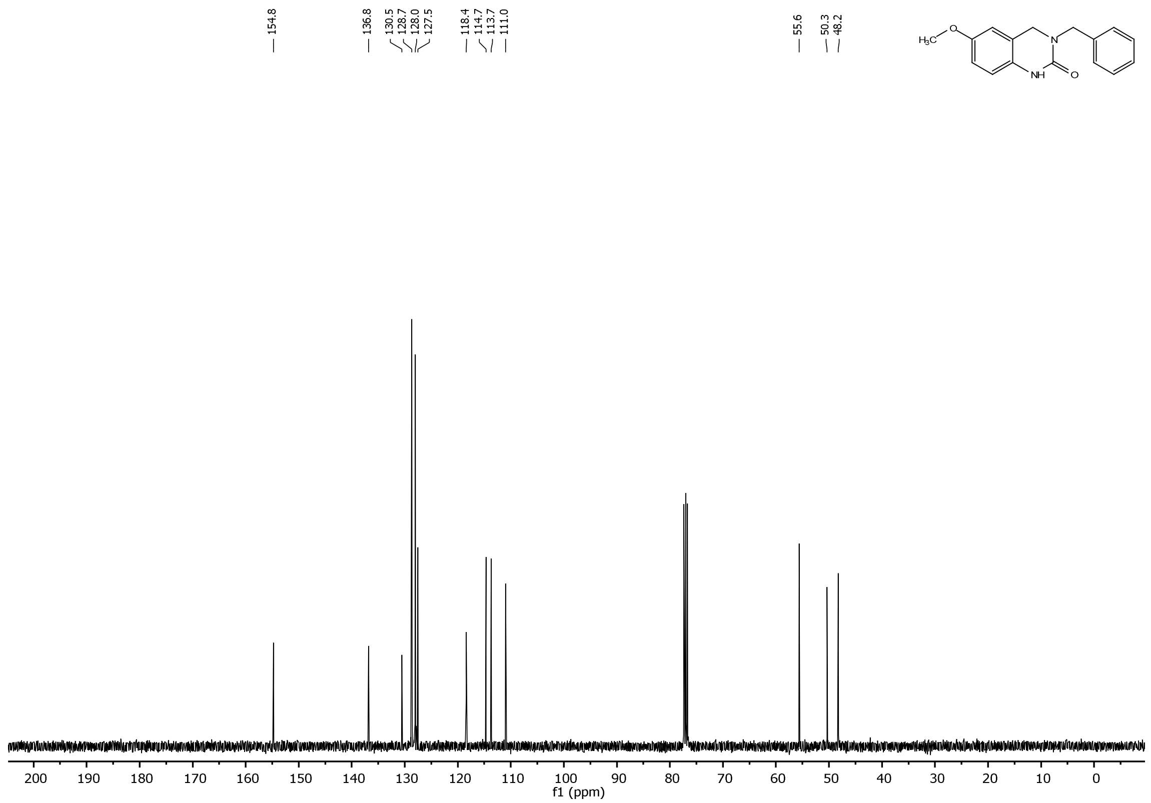

Compound 4b

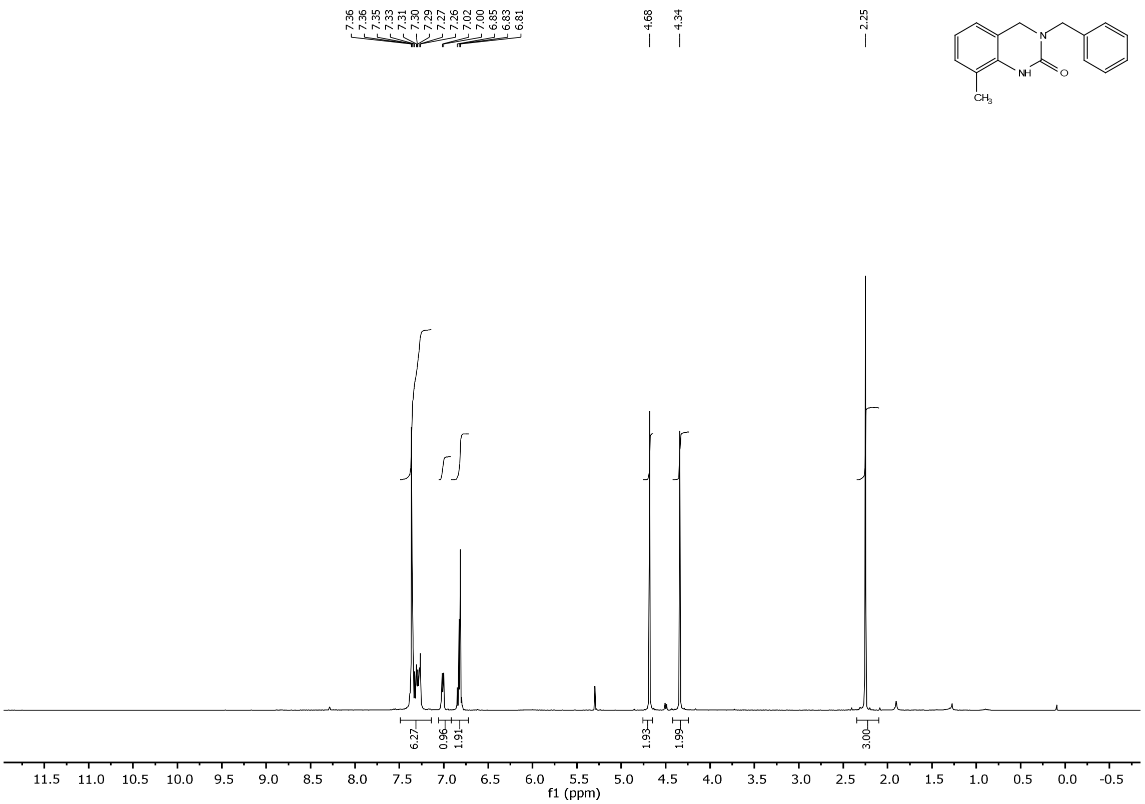

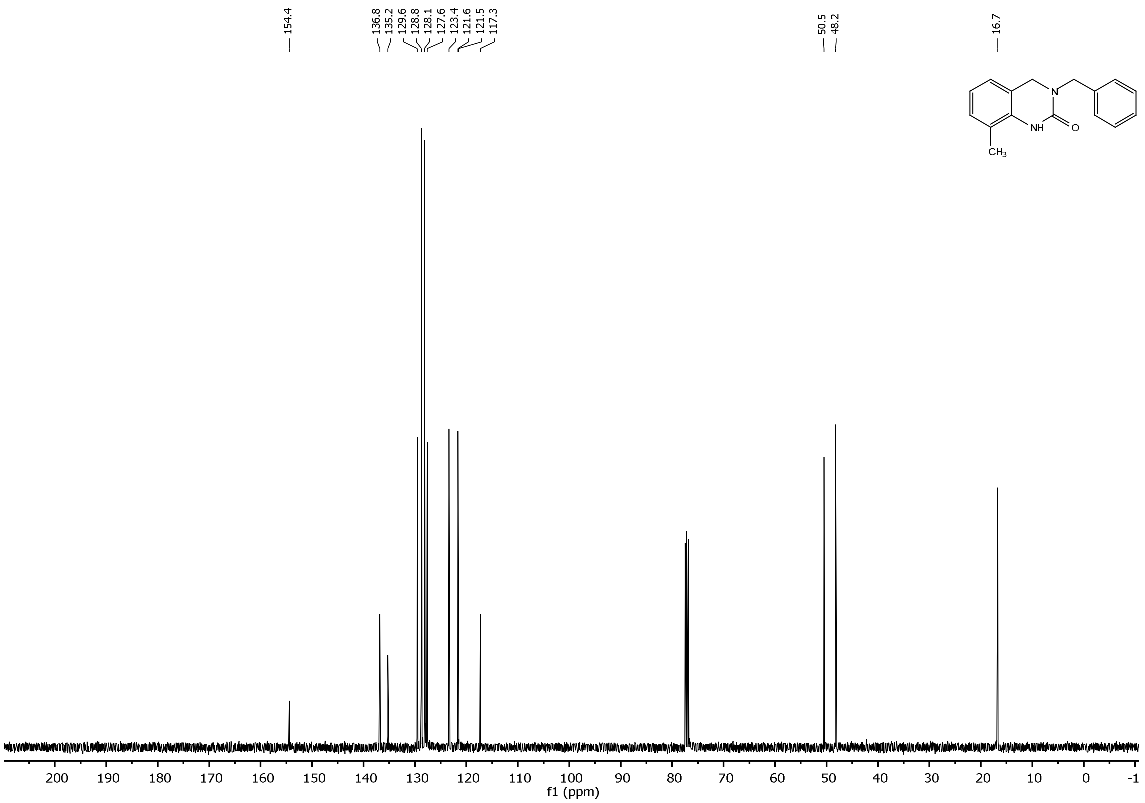

Compound 4c

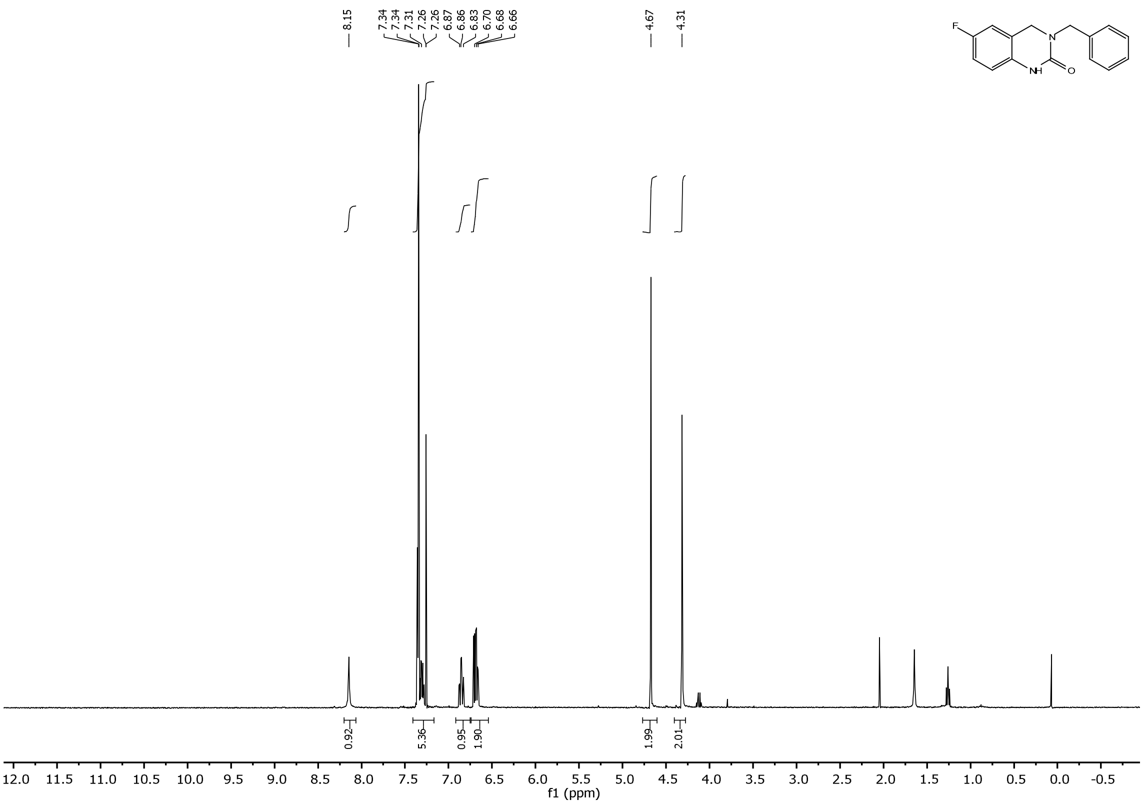

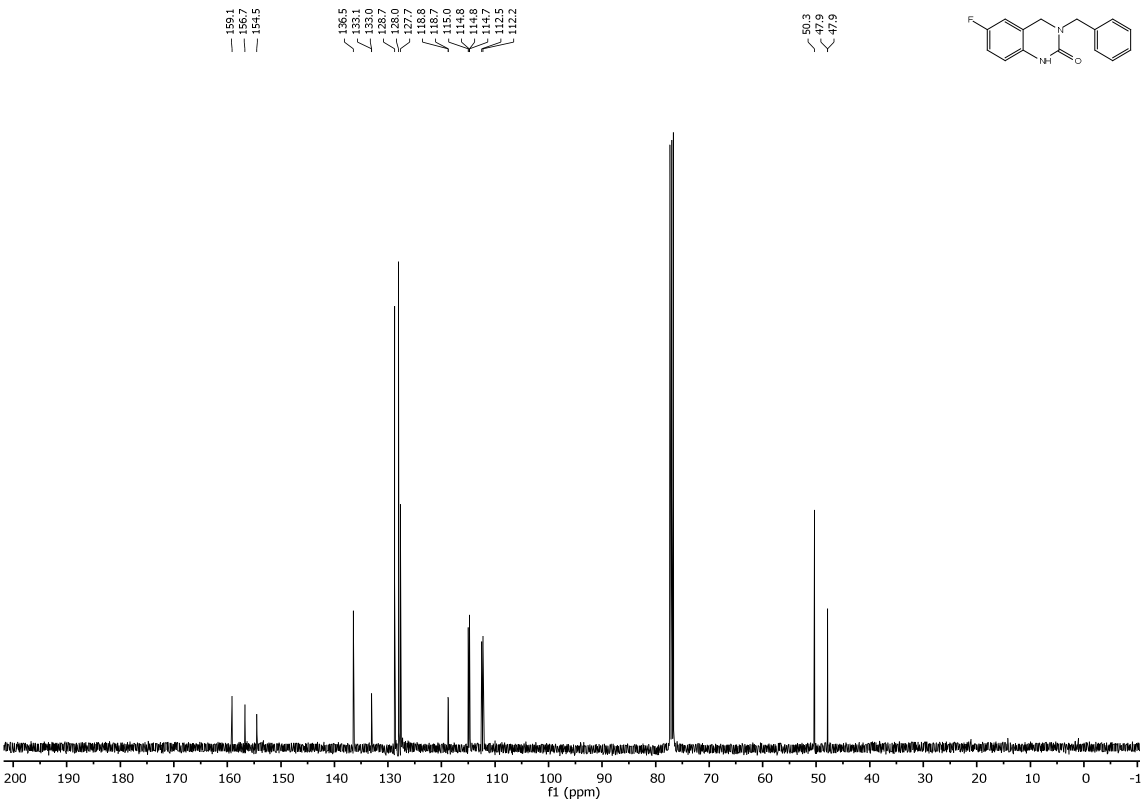

Compound 4d

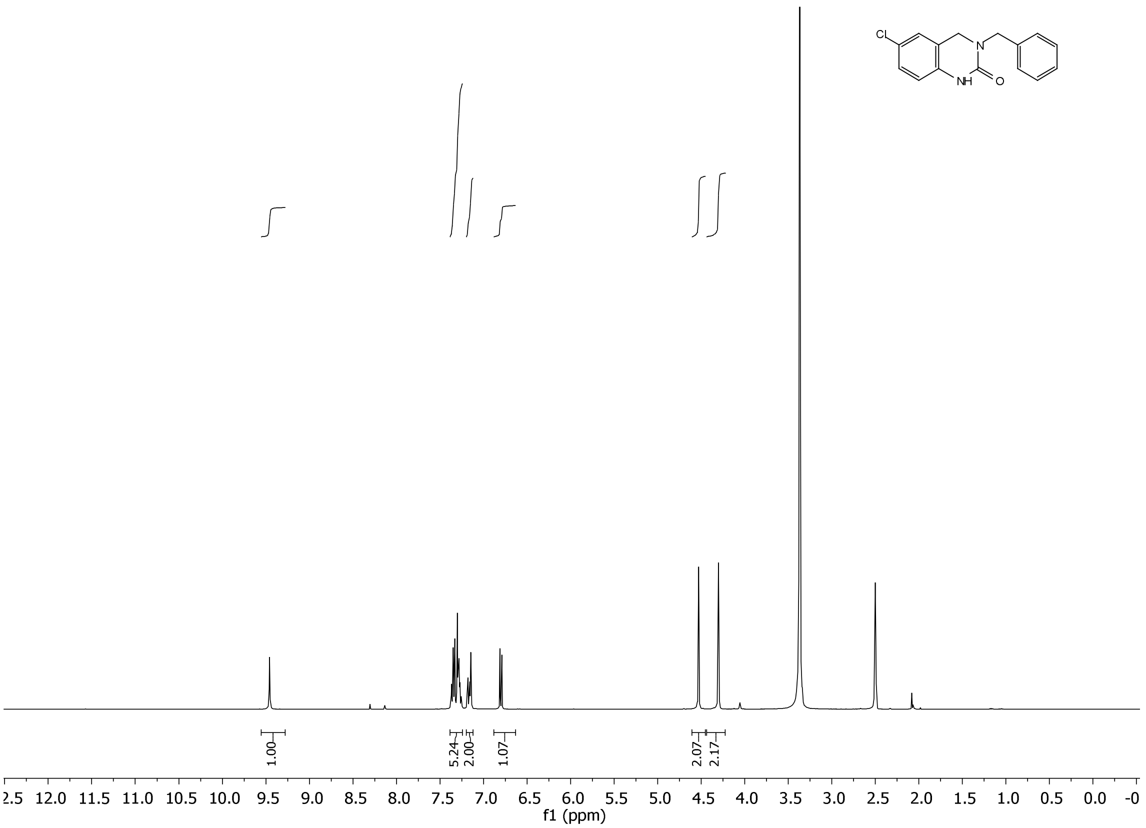
Cl
N
NH
O
1.00
5.24
2.00
1.07
2.07
2.17
2.5 12.0 11.5 11.0 10.5 10.0 9.5 9.0 8.5 8.0 7.5 7.0 6.5 6.0 5.5 5.0 4.5 4.0 3.5 3.0 2.5 2.0 1.5 1.0 0.5 0.0 -0
f1 (ppm)

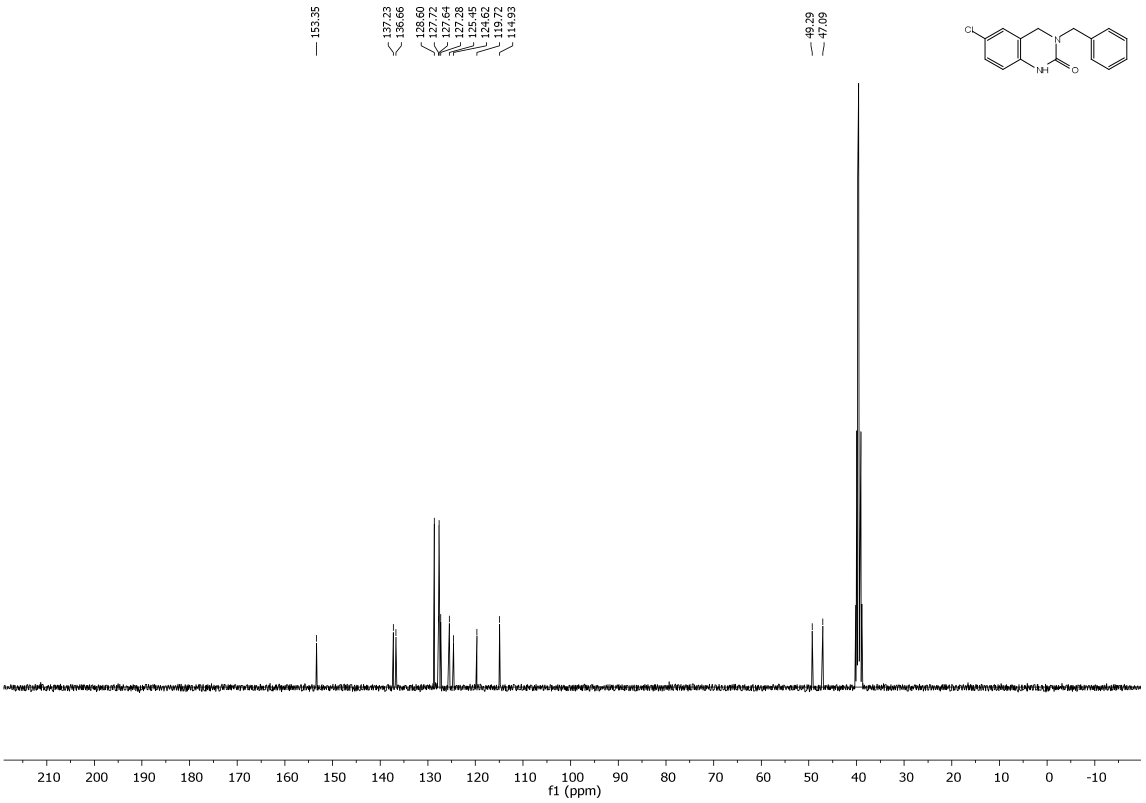
153.35
137.23
136.66
128.60
127.72
127.64
127.28
125.45
124.62
119.72
114.93
49.29
47.09
Cl
N
NH
O
210 200 190 180 170 160 150 140 130 120 110 100 90 80 70 60 50 40 30 20 10 0 -10
f1 (ppm)

Compound 4e

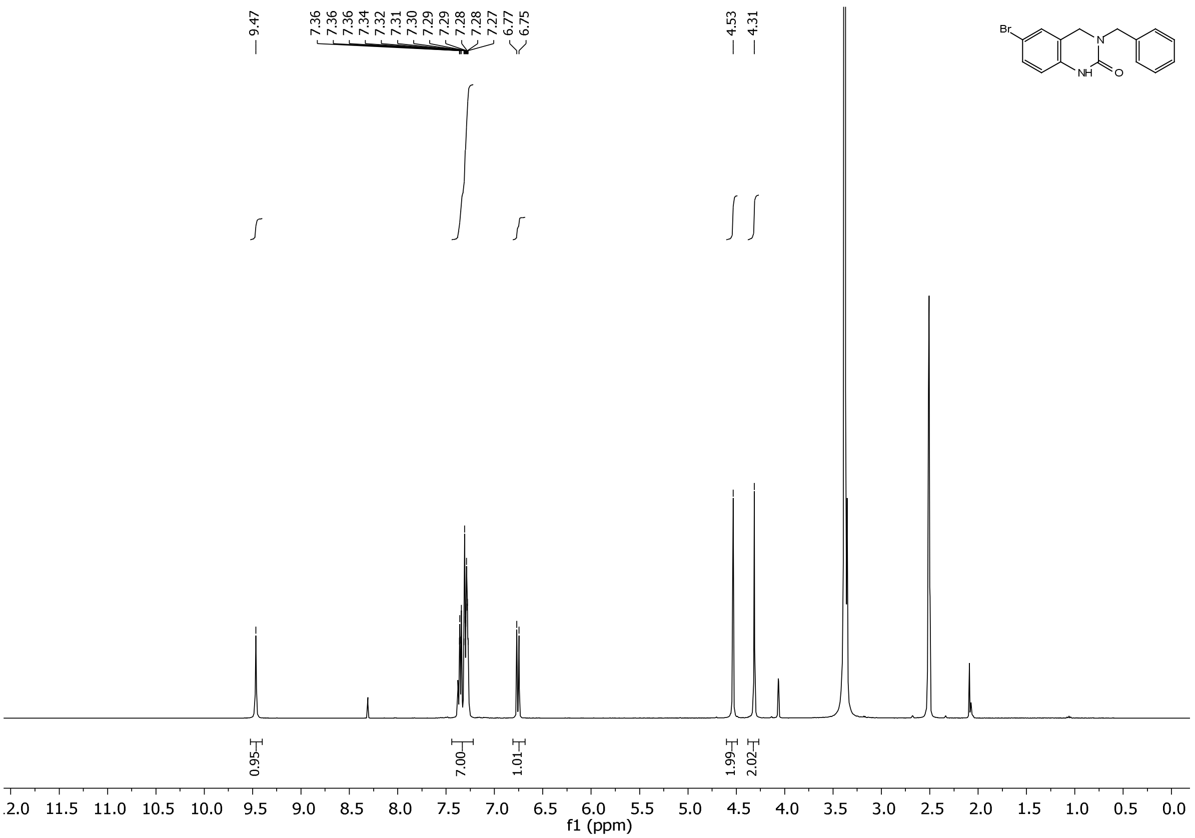
9.47
7.36
7.36
7.36
7.34
7.32
7.31
7.30
7.29
7.29
7.28
7.28
7.27
6.77
6.75
4.53
4.31
0.95
7.00
1.01
1.99
2.02
f1 (ppm)
Br
N
NH
O

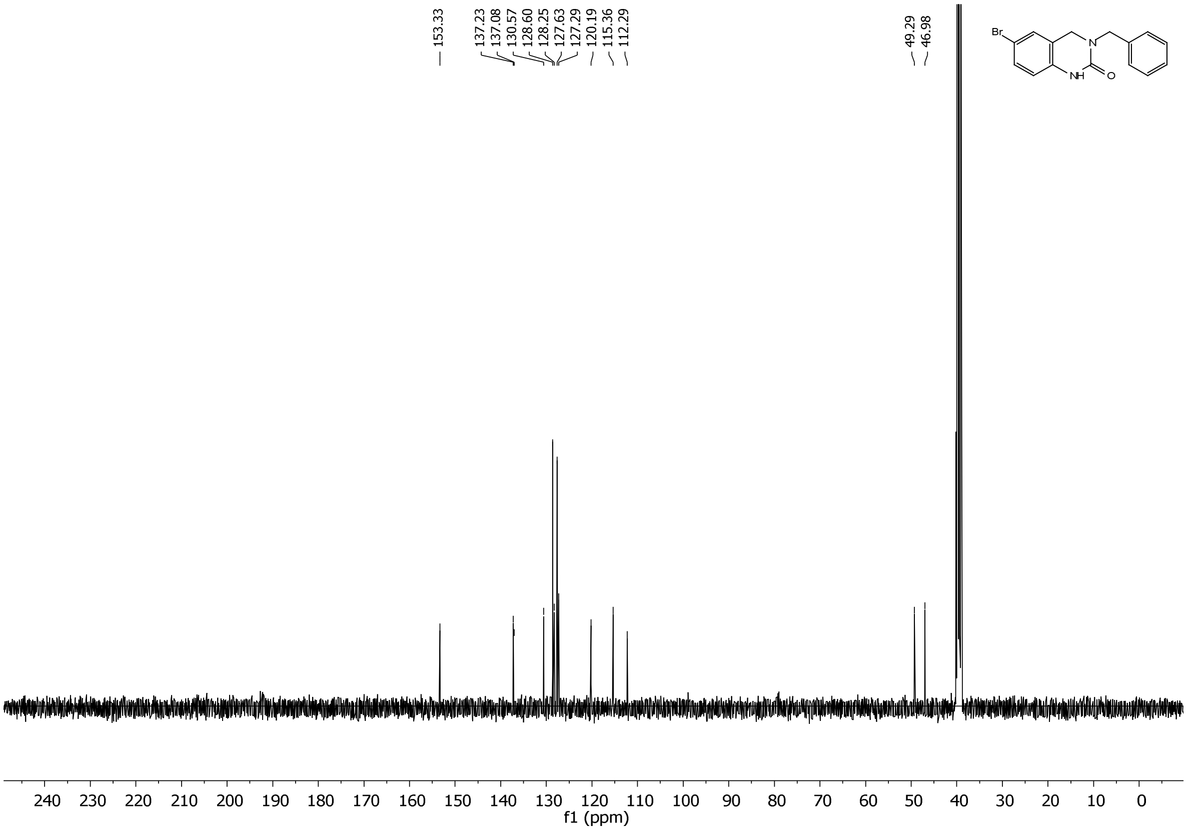
153.33
137.23
137.08
130.57
128.60
128.25
127.63
127.29
120.19
115.36
112.29
49.29
46.98
f1 (ppm)
Br
N
NH
O

Compound 4f

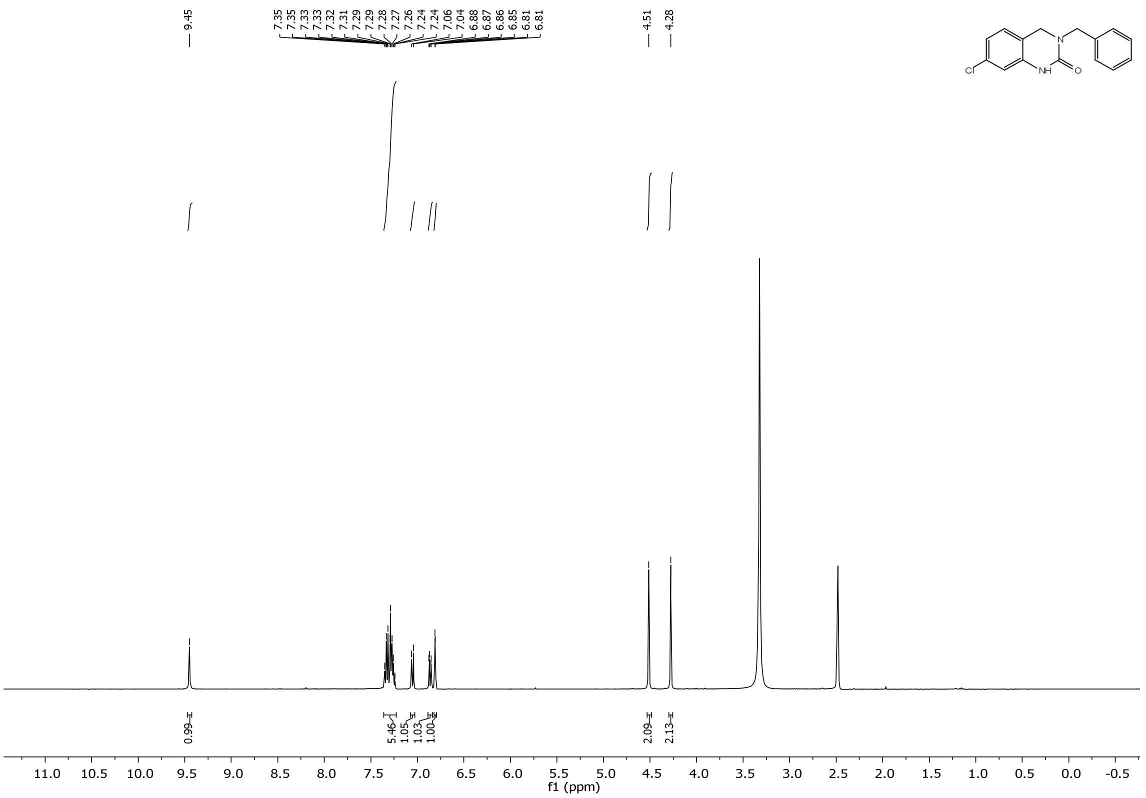

9.45
7.35
7.35
7.33
7.33
7.32
7.31
7.29
7.29
7.28
7.27
7.26
7.24
7.24
7.06
7.04
6.88
6.87
6.86
6.85
6.81
6.81
4.51
4.28
Cl
NH
N
O
0.99
5.46
1.05
1.03
1.00
2.09
2.13
f1 (ppm)

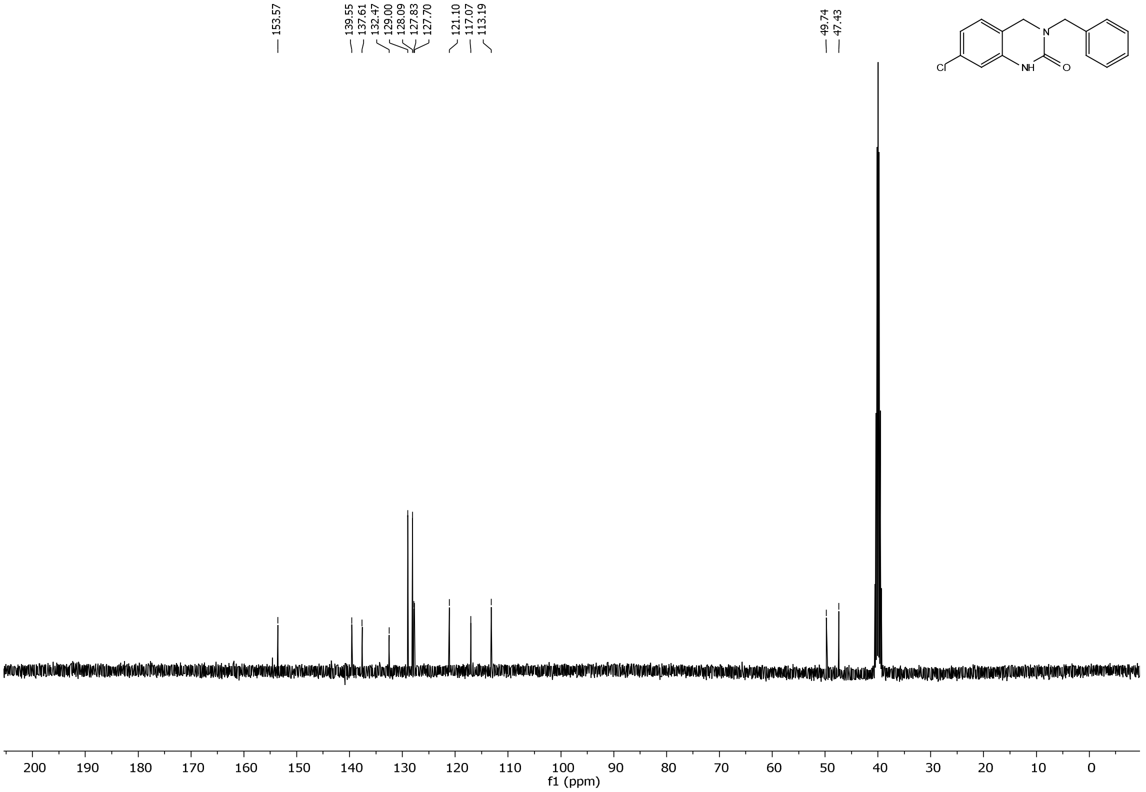

153.57
139.55
137.61
132.47
129.00
128.09
127.83
127.70
121.10
117.07
113.19
49.74
47.43
Cl
NH
N
O
f1 (ppm)

Compound 4g

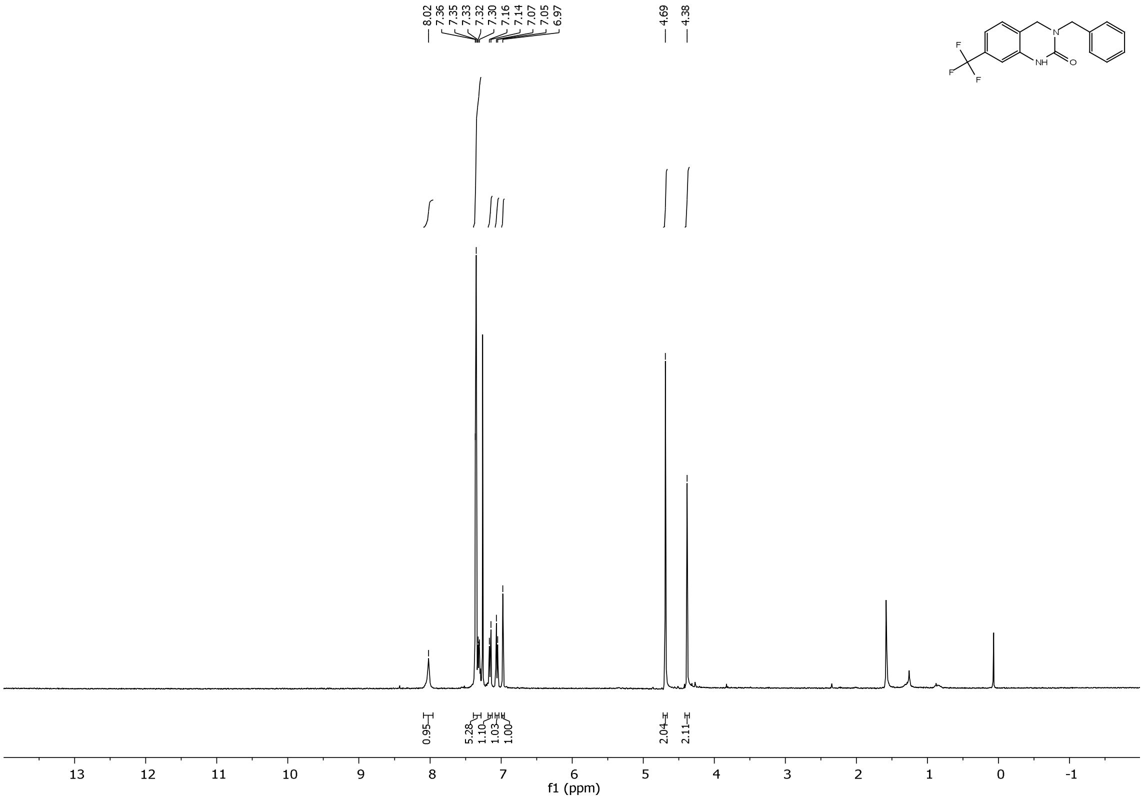
8.02
7.36
7.35
7.33
7.32
7.30
7.16
7.14
7.07
7.05
6.97
4.69
4.38
0.95
5.28
1.10
1.03
1.00
2.04
2.11
f1 (ppm)
F
F
F
N
NH
O

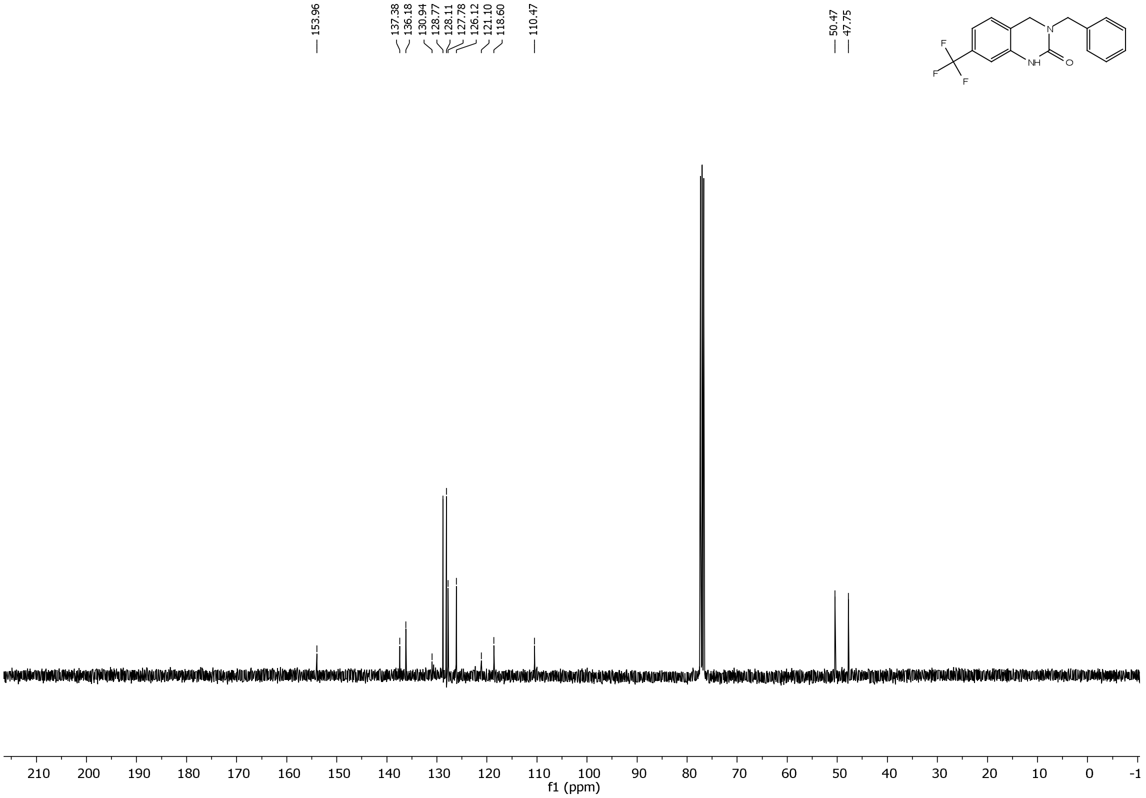
153.96
137.38
136.18
130.94
128.77
128.11
127.78
126.12
121.10
118.60
110.47
50.47
47.75
f1 (ppm)
F
F
F
N
NH
O

Compound 4h

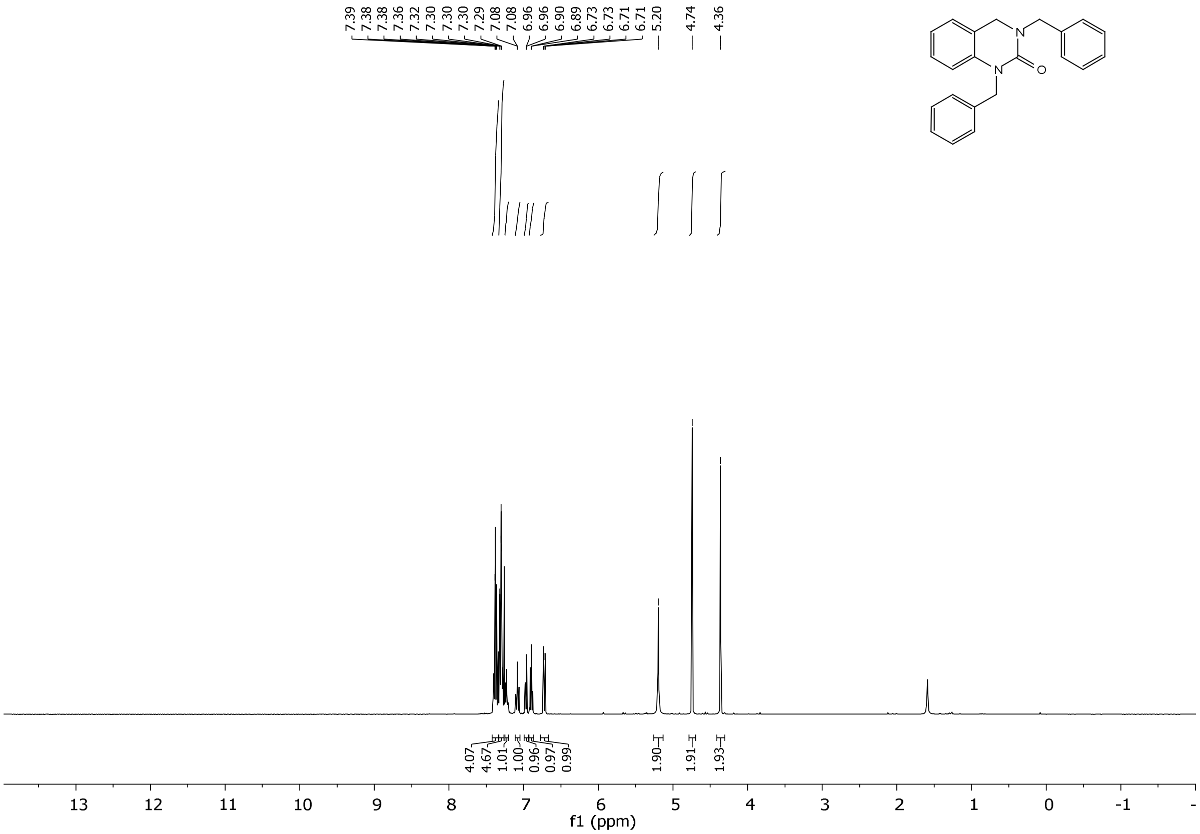
7.39
7.38
7.38
7.36
7.32
7.30
7.30
7.30
7.29
7.08
7.08
6.96
6.96
6.90
6.89
6.73
6.73
6.71
6.71
5.20
4.74
4.36
N
N
O
4.07
4.67
1.01
1.00
0.96
0.97
0.99
1.90
1.91
1.93
13
12
11
10
9
8
7
6
5
4
3
2
1
0
-1
f1 (ppm)

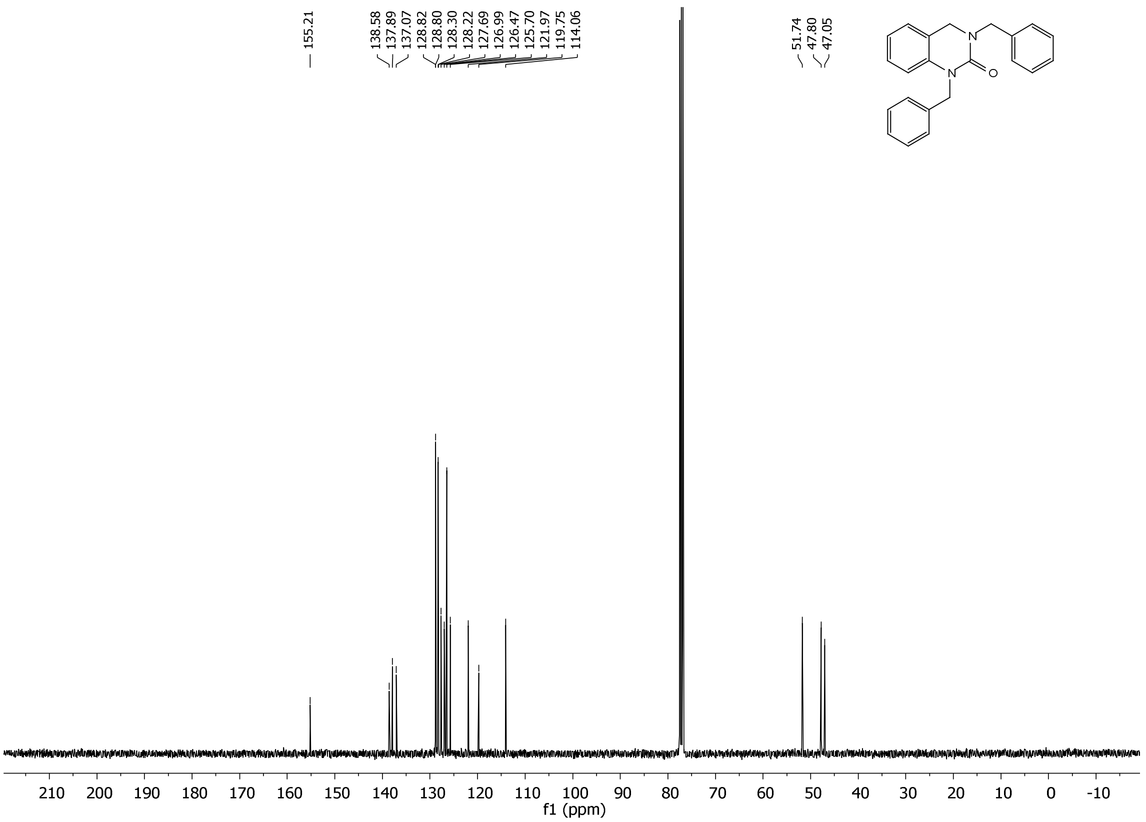
155.21
138.58
137.89
137.07
128.82
128.80
128.30
128.22
127.69
126.99
126.47
125.70
121.97
119.75
114.06
51.74
47.80
47.05
N
N
O
210
200
190
180
170
160
150
140
130
120
110
100
90
80
70
60
50
40
30
20
10
0
-10
f1 (ppm)

Compound 4i

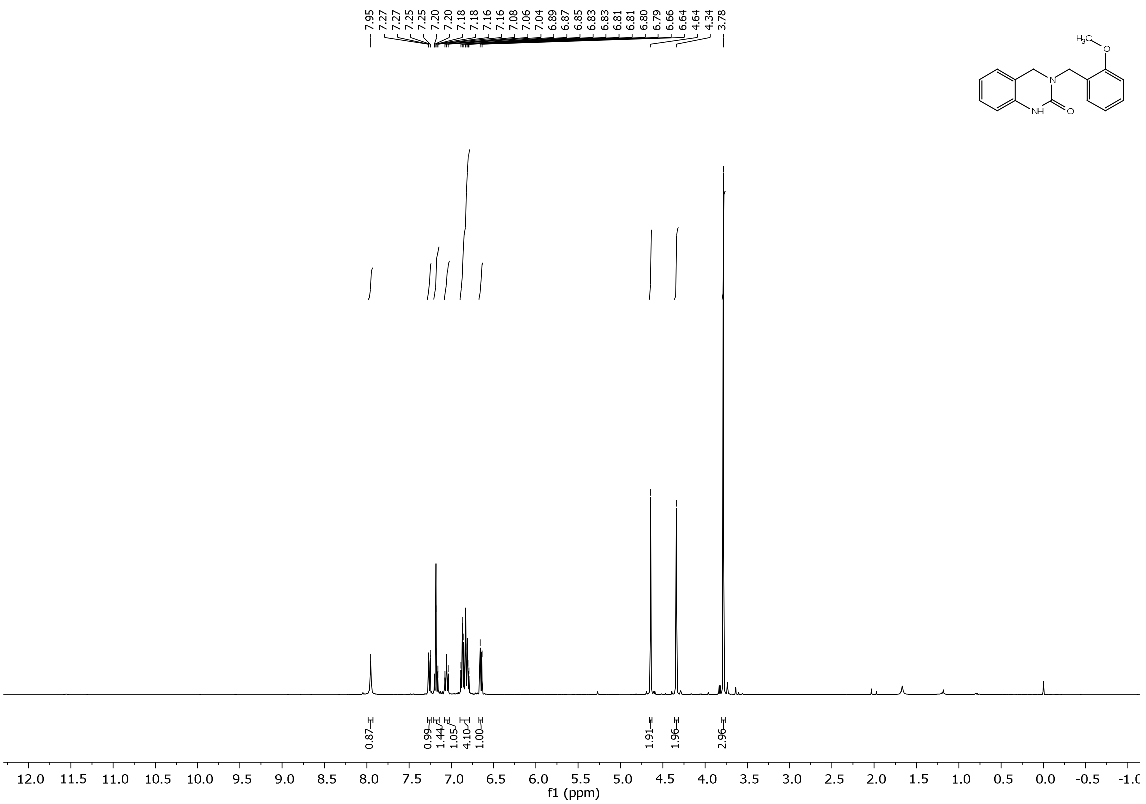
7.95
7.27
7.27
7.25
7.25
7.20
7.20
7.18
7.18
7.16
7.16
7.08
7.06
7.04
6.89
6.87
6.85
6.83
6.83
6.81
6.81
6.80
6.79
6.66
6.64
4.64
4.34
3.78
0.87
0.99
1.44
1.05
4.10
1.00
1.91
1.96
2.96
f1 (ppm)

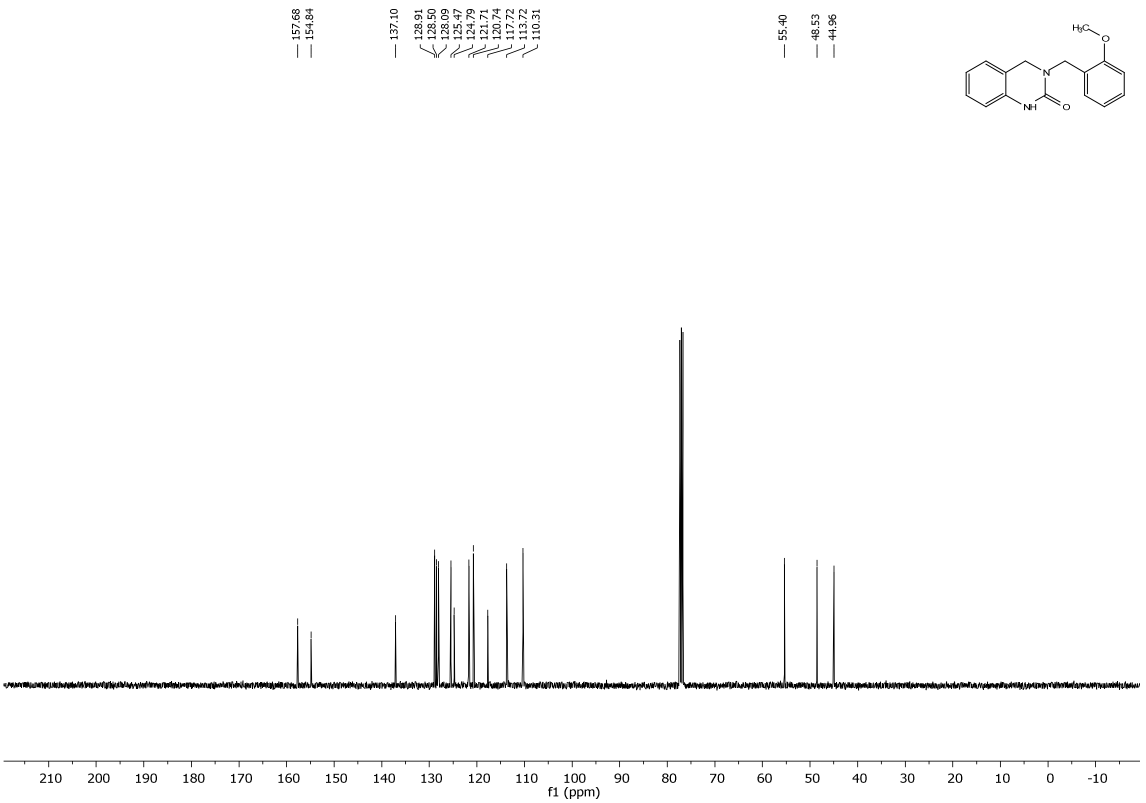
157.68
154.84
137.10
128.91
128.50
128.09
125.47
124.79
121.71
120.74
117.72
113.72
110.31
55.40
48.53
44.96
f1 (ppm)

Compound 4j

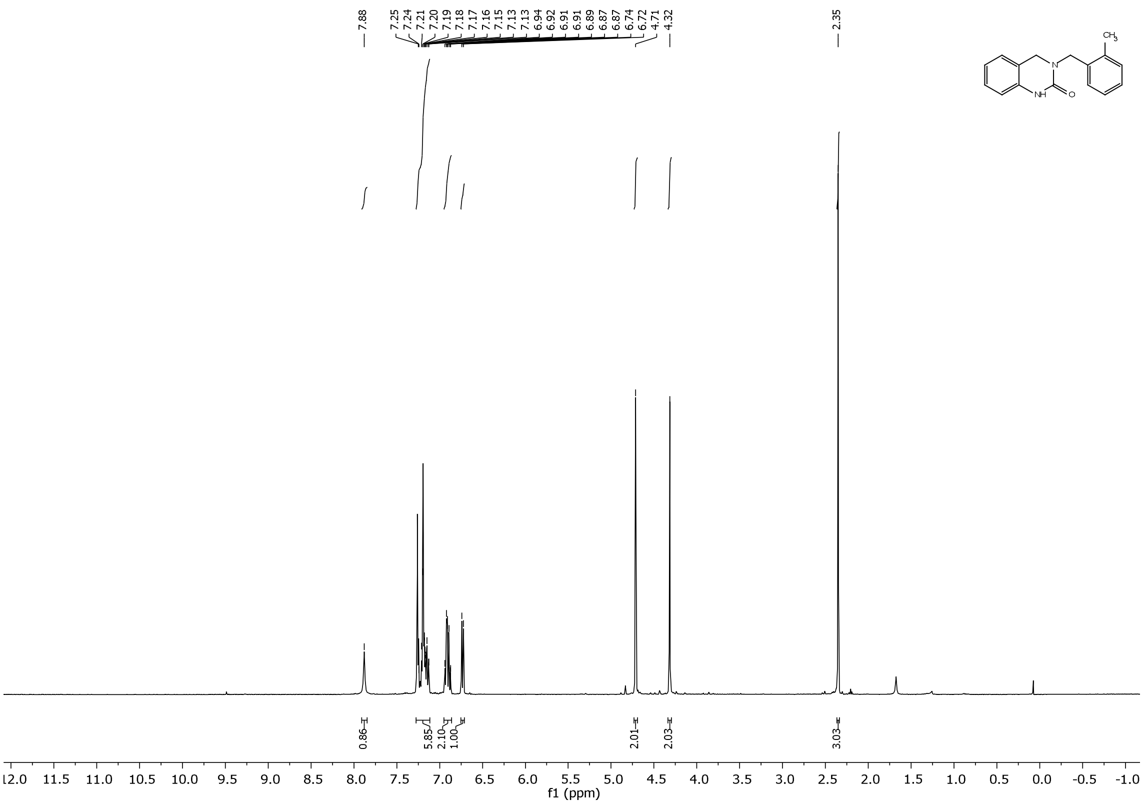

7.88
7.25
7.24
7.21
7.20
7.19
7.18
7.17
7.16
7.15
7.13
7.13
6.94
6.92
6.91
6.91
6.89
6.87
6.87
6.74
6.72
4.71
4.32
2.35
0.86
5.85
2.10
1.00
2.01
2.03
3.03
f1 (ppm)
CH3
N
NH
O

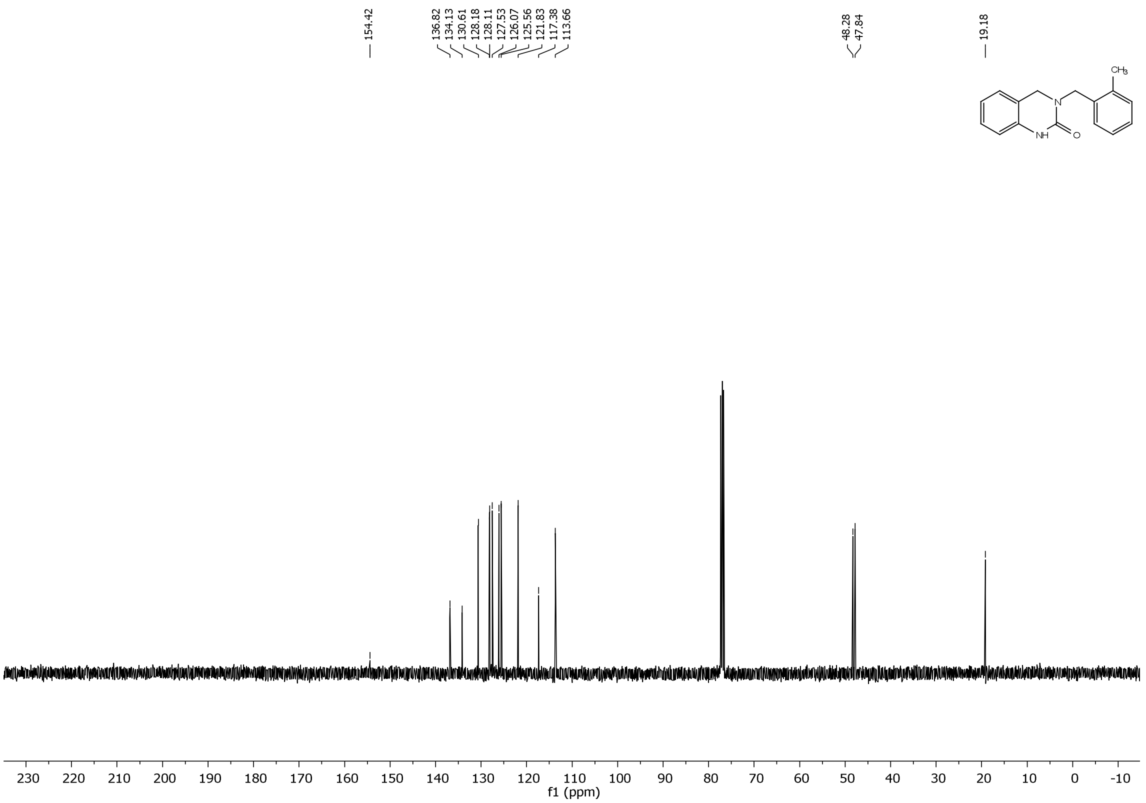

154.42
136.82
134.13
130.61
128.18
128.11
127.53
126.07
125.56
121.83
117.38
113.66
48.28
47.84
19.18
f1 (ppm)
CH3
N
NH
O

Compound 4k

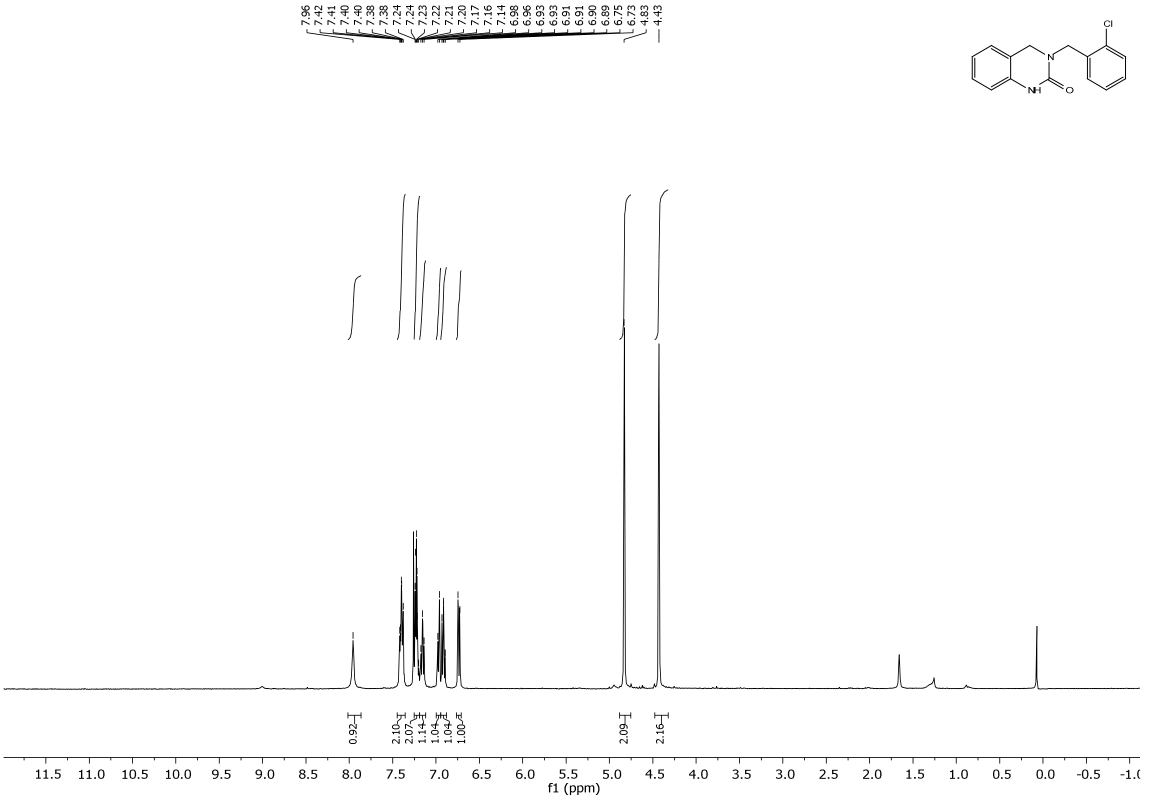

7.96
7.42
7.41
7.40
7.38
7.38
7.24
7.24
7.23
7.22
7.21
7.20
7.17
7.16
7.14
6.98
6.96
6.93
6.93
6.91
6.91
6.90
6.89
6.75
6.73
4.83
4.43
Cl
N
NH
O
0.92
2.10
2.07
1.14
1.04
1.04
1.00
2.09
2.16
11.5 11.0 10.5 10.0 9.5 9.0 8.5 8.0 7.5 7.0 6.5 6.0 5.5 5.0 4.5 4.0 3.5 3.0 2.5 2.0 1.5 1.0 0.5 0.0 -0.5
f1 (ppm)

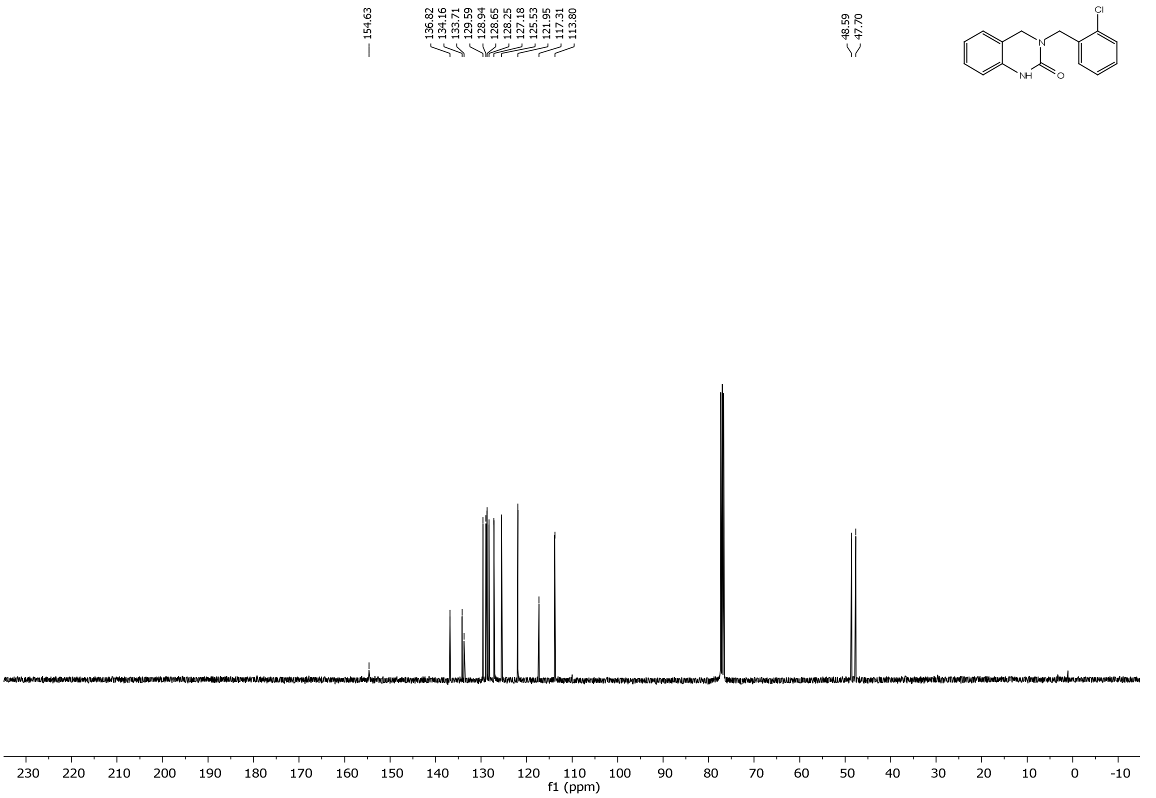

154.63
136.82
134.16
133.71
129.59
128.94
128.65
128.25
127.18
125.53
121.95
117.31
113.80
48.59
47.70
Cl
N
NH
O
230 220 210 200 190 180 170 160 150 140 130 120 110 100 90 80 70 60 50 40 30 20 10 0 -10
f1 (ppm)

Compound 4l

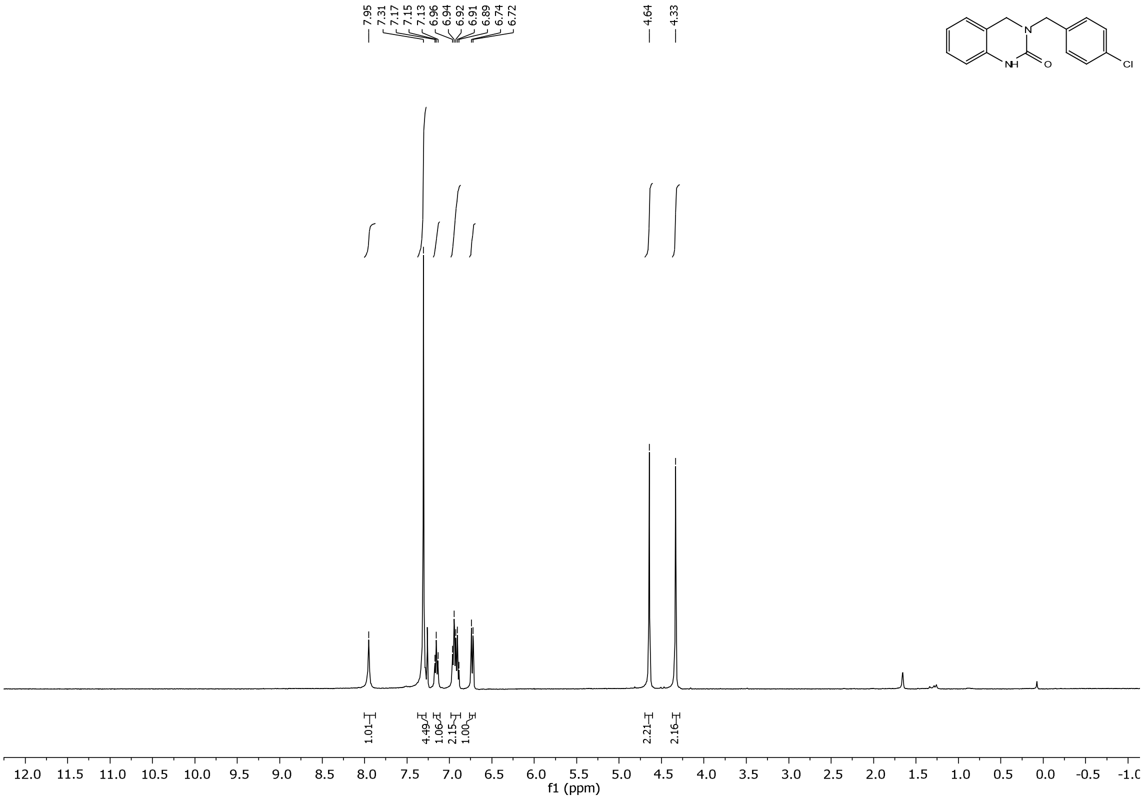

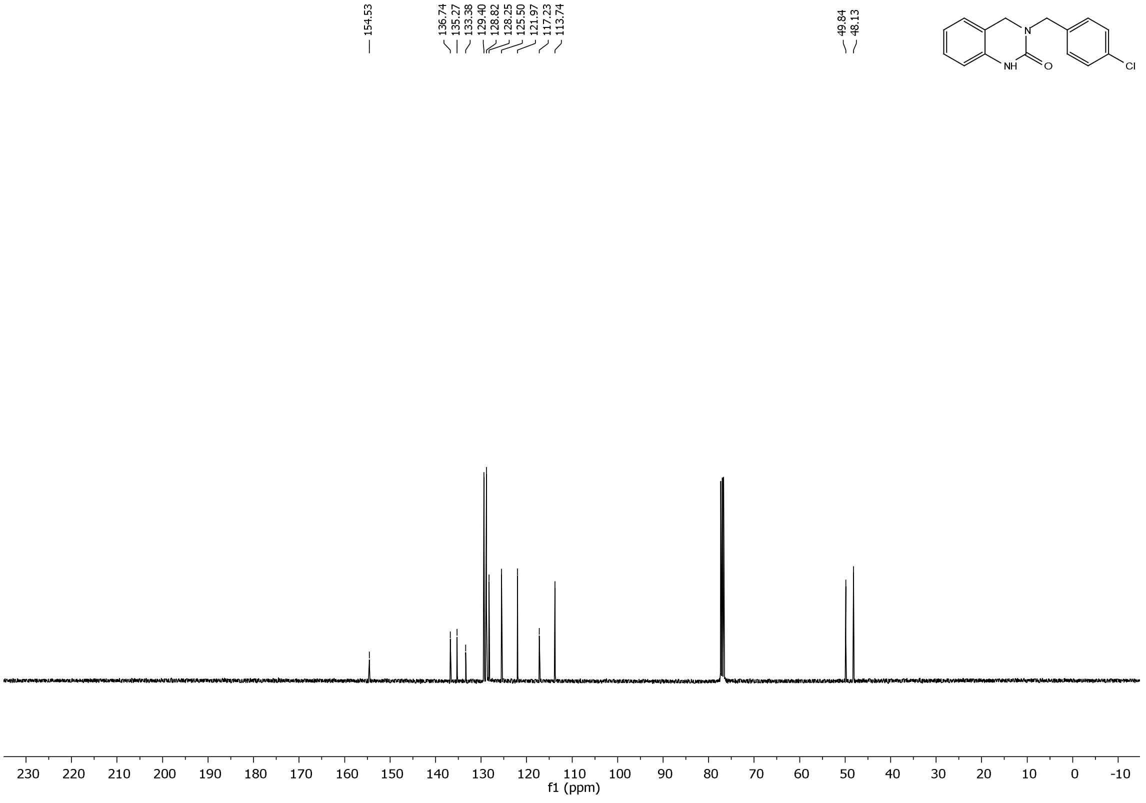

Compound 4m

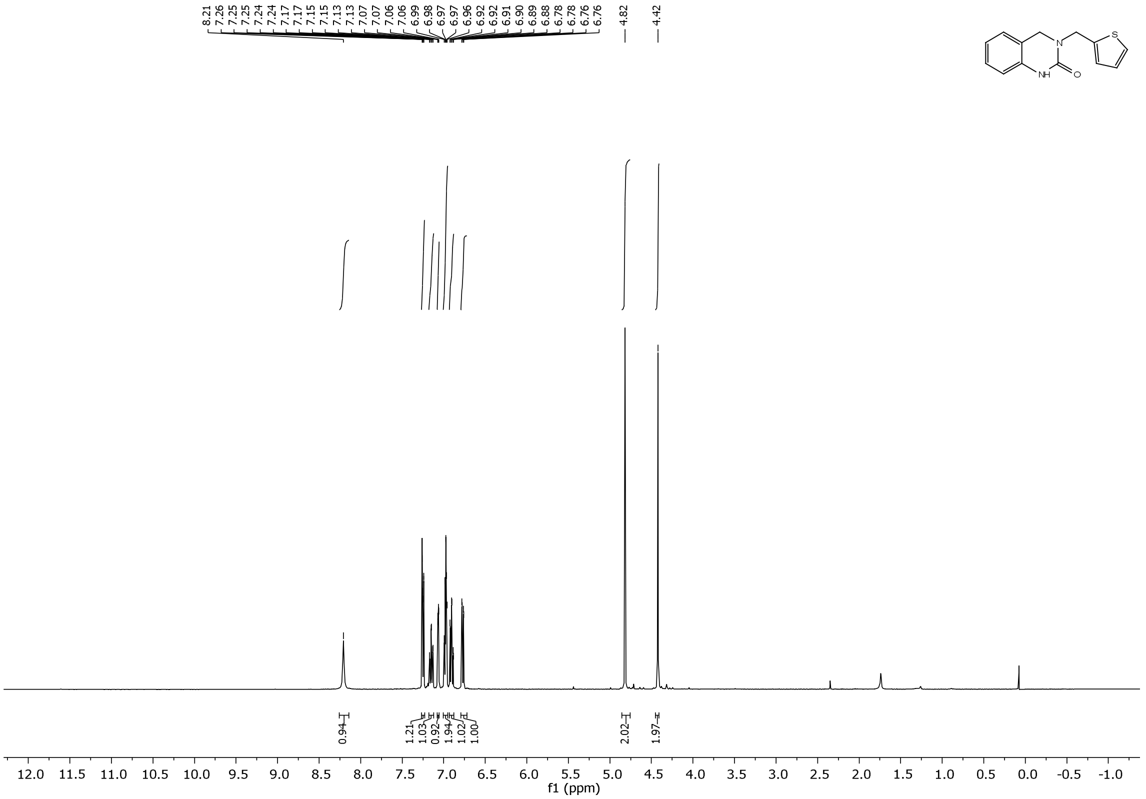

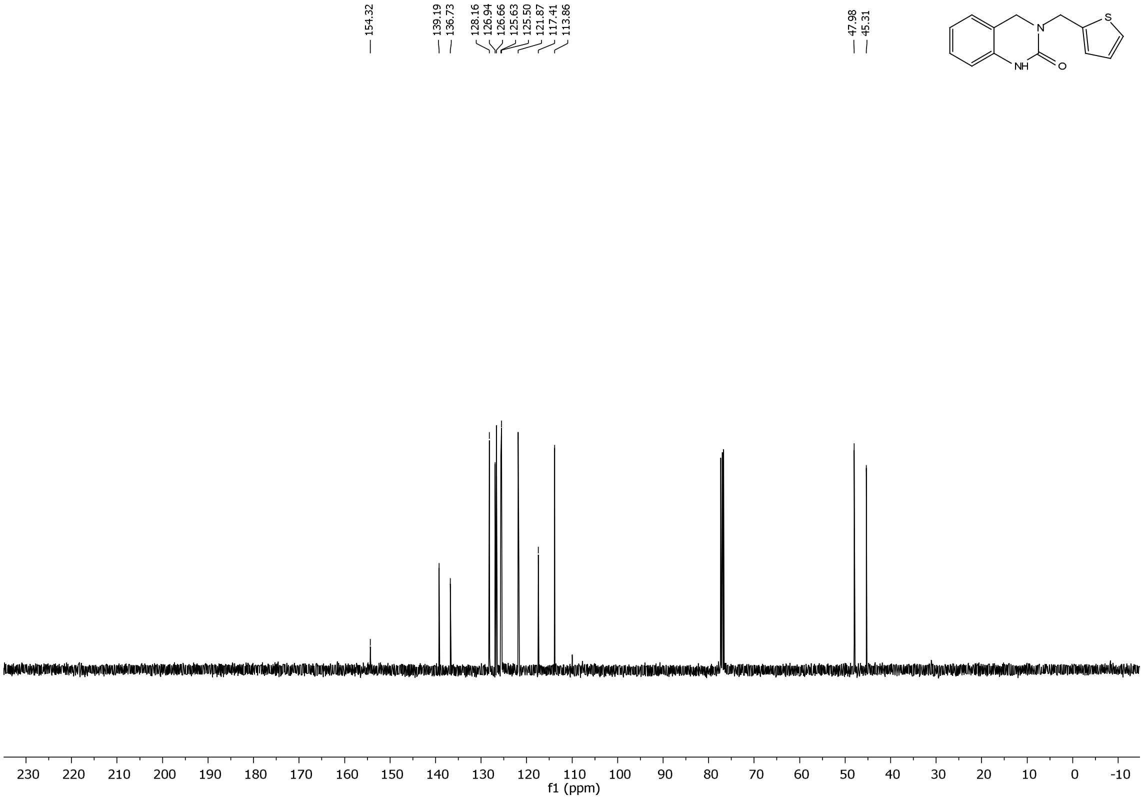

Compound 4n

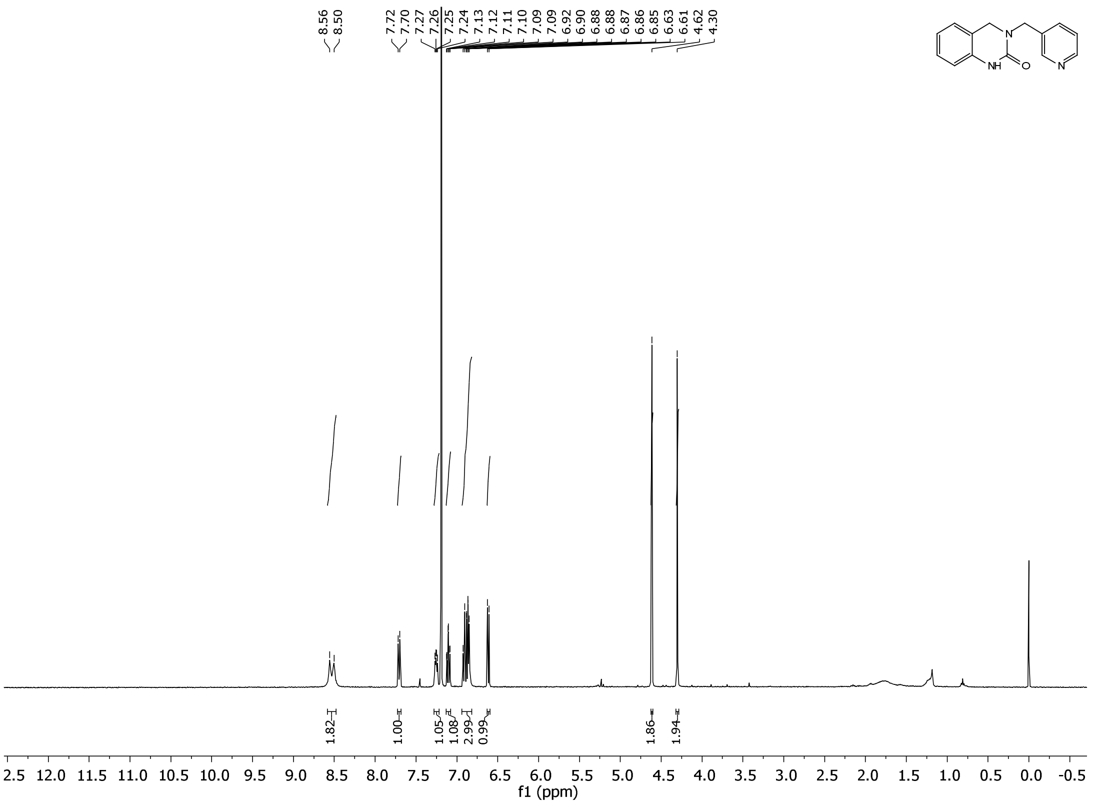
8.56
8.50
7.72
7.70
7.27
7.26
7.25
7.24
7.13
7.12
7.11
7.10
7.09
7.09
6.92
6.90
6.88
6.88
6.87
6.86
6.85
6.63
6.61
4.62
4.30
NH
O
N
N
1.82
1.00
1.05
1.08
2.99
0.99
1.86
1.94
f1 (ppm)

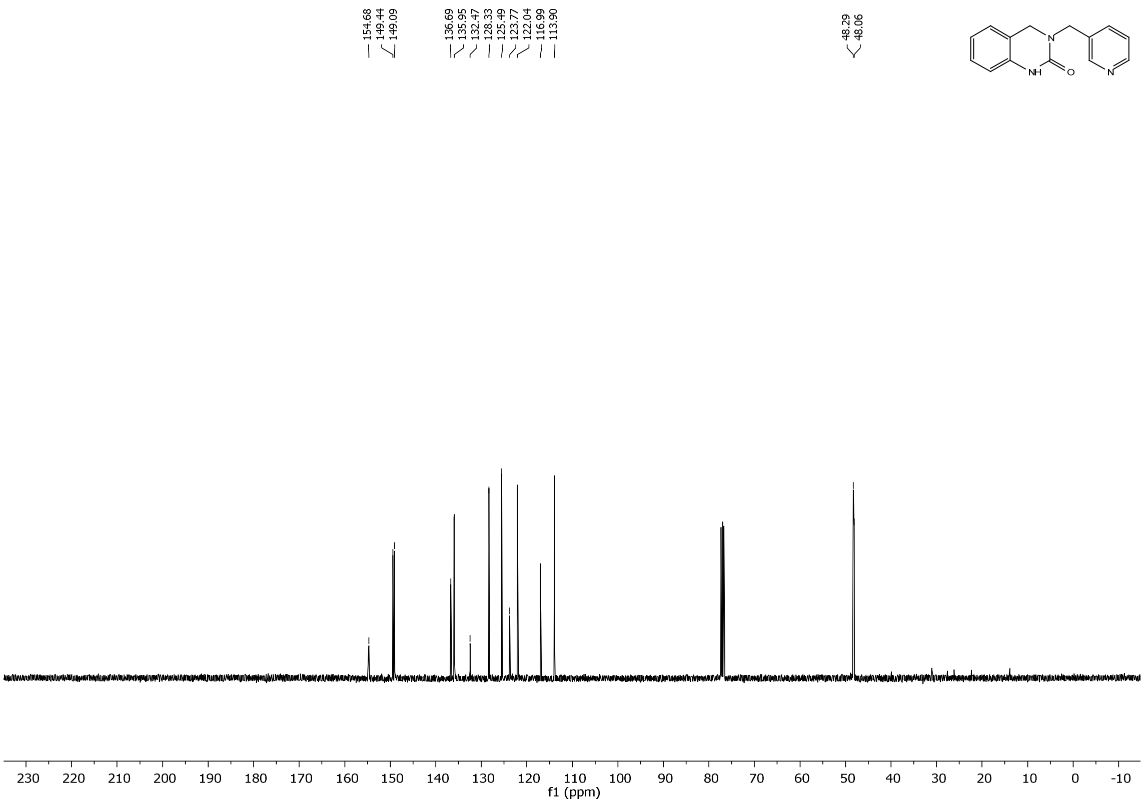
154.68
149.44
149.09
136.69
135.95
132.47
128.33
125.49
123.77
122.04
116.99
113.90
48.29
48.06
NH
O
N
N
f1 (ppm)

Compound 4o

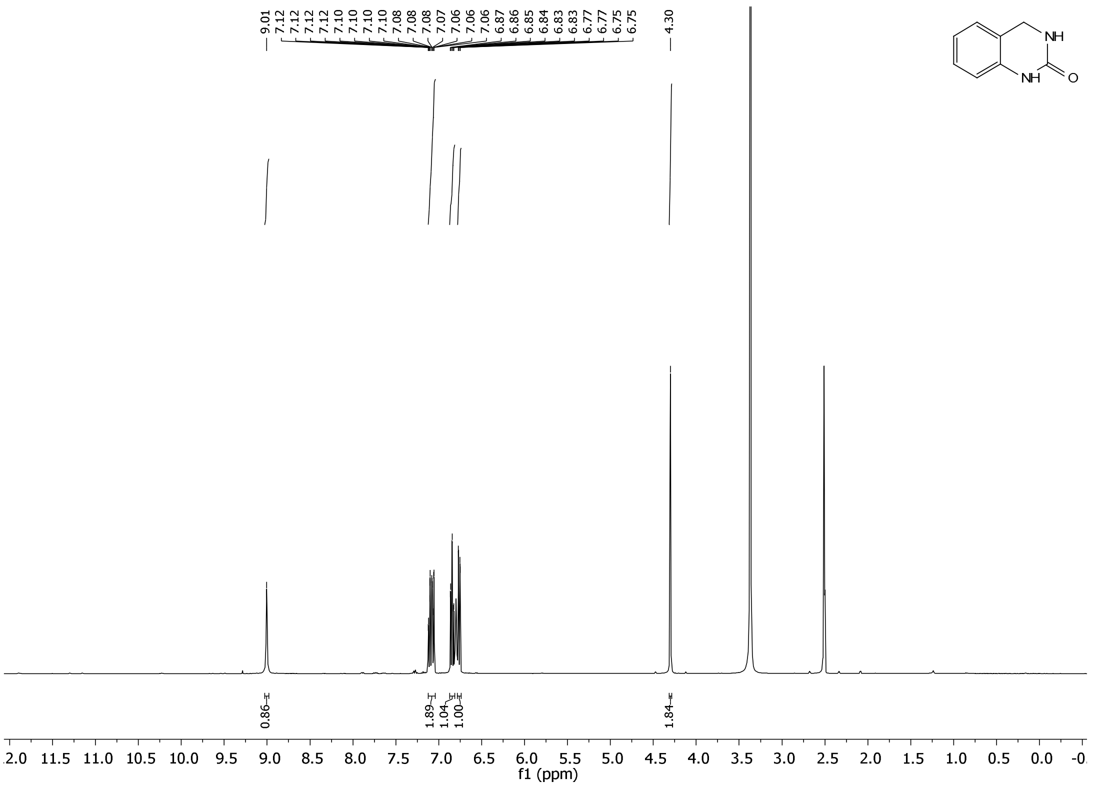

9.01
7.12
7.12
7.12
7.12
7.10
7.10
7.10
7.10
7.08
7.08
7.08
7.07
7.06
7.06
7.06
6.87
6.86
6.85
6.84
6.83
6.83
6.77
6.77
6.75
6.75
4.30
NH
NH
O
0.86
1.89
1.04
1.00
1.84
f1 (ppm)

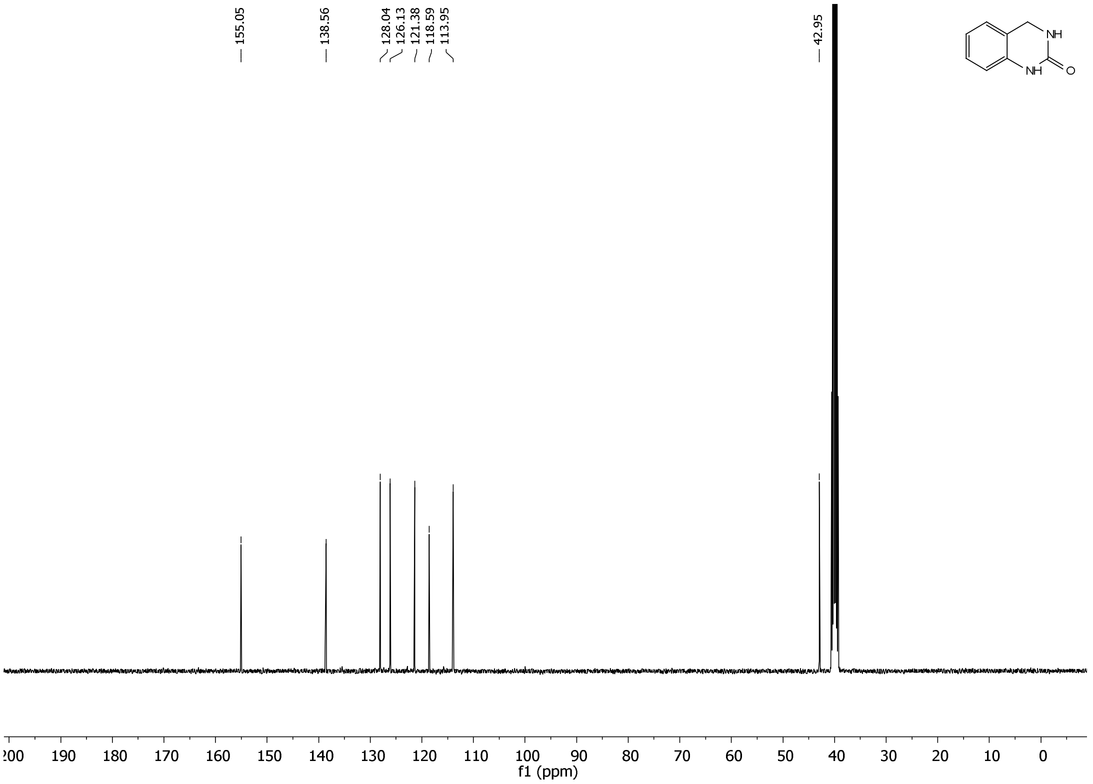

155.05
138.56
128.04
126.13
121.38
118.59
113.95
42.95
NH
NH
O
f1 (ppm)

Compound 5a

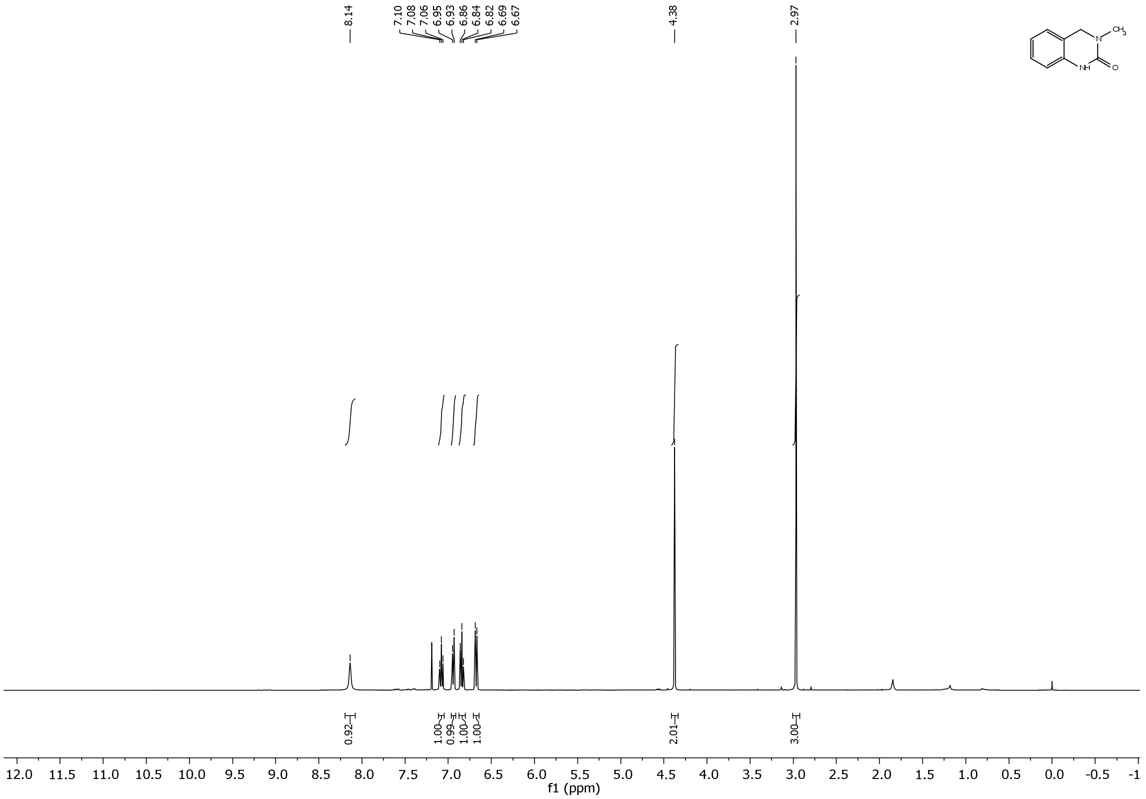
8.14
7.10
7.08
7.06
6.95
6.93
6.86
6.84
6.82
6.69
6.67
4.38
2.97
CH3
NH
O
0.92
1.00
0.99
1.00
1.00
2.01
3.00
12.0 11.5 11.0 10.5 10.0 9.5 9.0 8.5 8.0 7.5 7.0 6.5 6.0 5.5 5.0 4.5 4.0 3.5 3.0 2.5 2.0 1.5 1.0 0.5 0.0 -0.5
f1 (ppm)

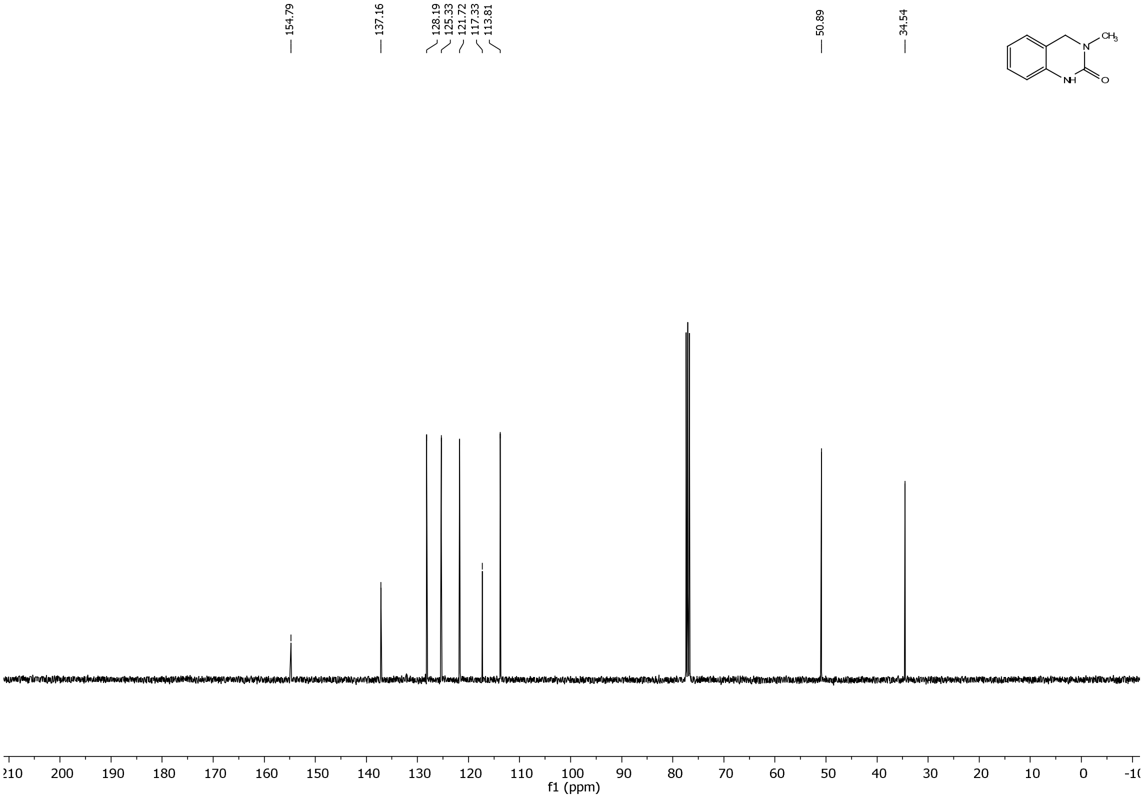
154.79
137.16
128.19
125.33
121.72
117.33
113.81
50.89
34.54
CH3
NH
O
200 190 180 170 160 150 140 130 120 110 100 90 80 70 60 50 40 30 20 10 0
f1 (ppm)

Compound 5b

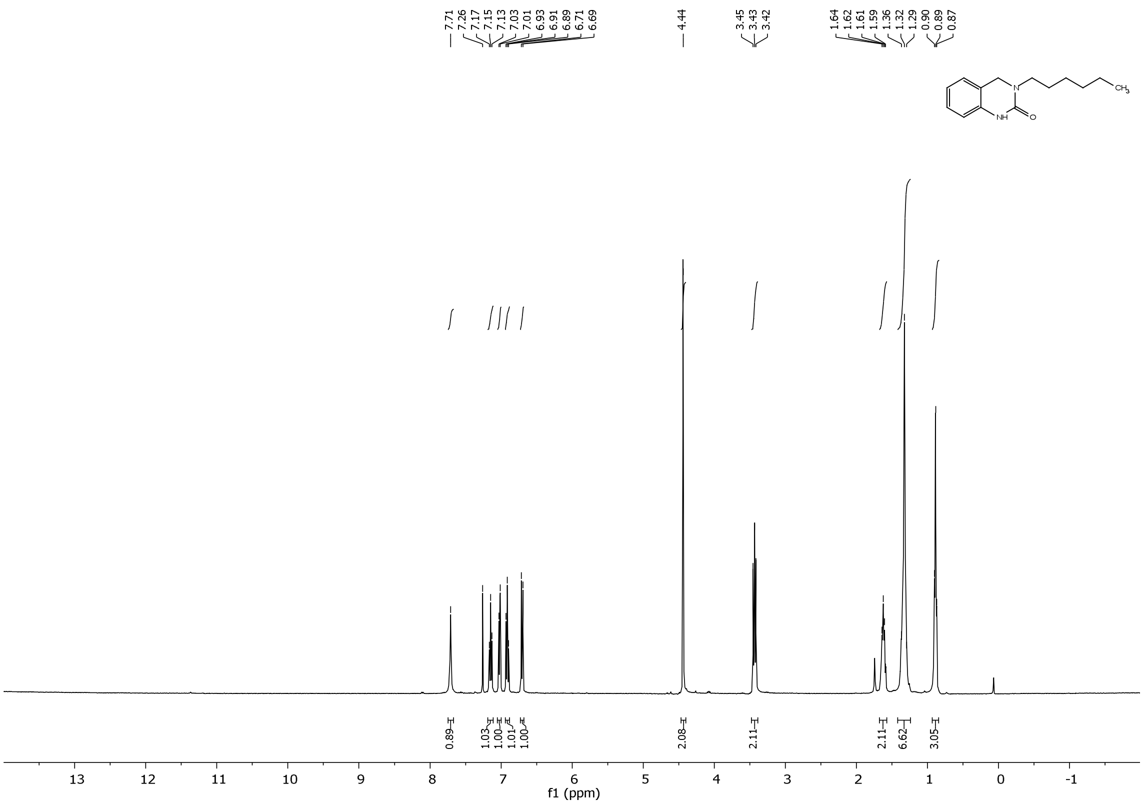
7.71
7.26
7.17
7.15
7.13
7.03
7.01
6.93
6.91
6.89
6.71
6.69
4.44
3.45
3.43
3.42
1.64
1.62
1.61
1.59
1.36
1.32
1.29
0.90
0.89
0.87
NH
O
N
CH3
0.89
1.03
1.00
1.01
1.00
2.08
2.11
2.11
6.62
3.05
f1 (ppm)

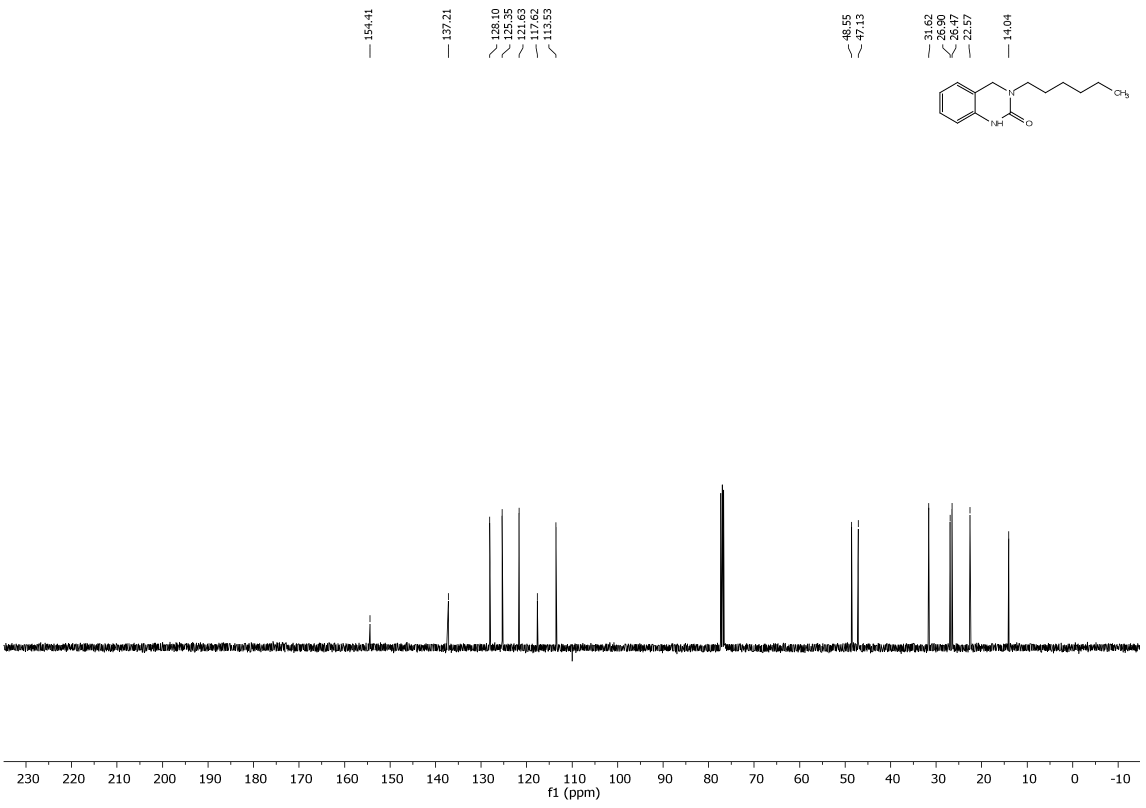
154.41
137.21
128.10
125.35
121.63
117.62
113.53
48.55
47.13
31.62
26.90
26.47
22.57
14.04
NH
O
N
CH3
f1 (ppm)

Compound 5c

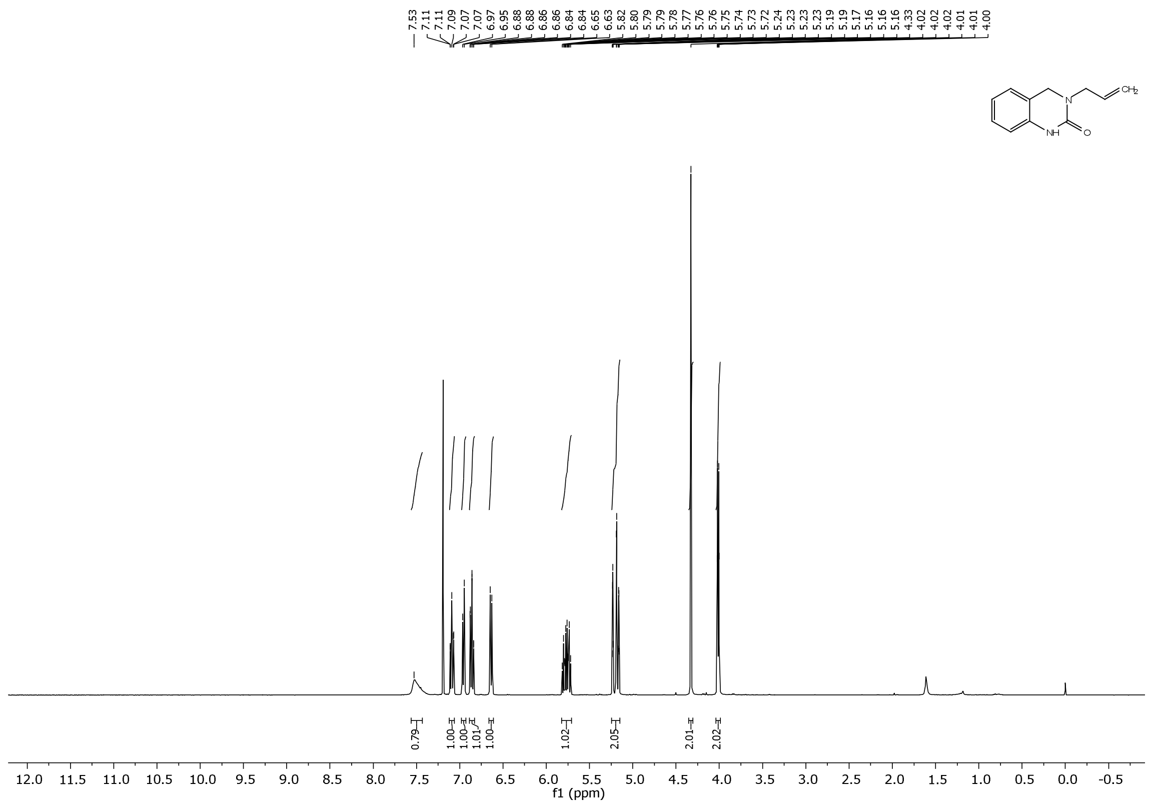

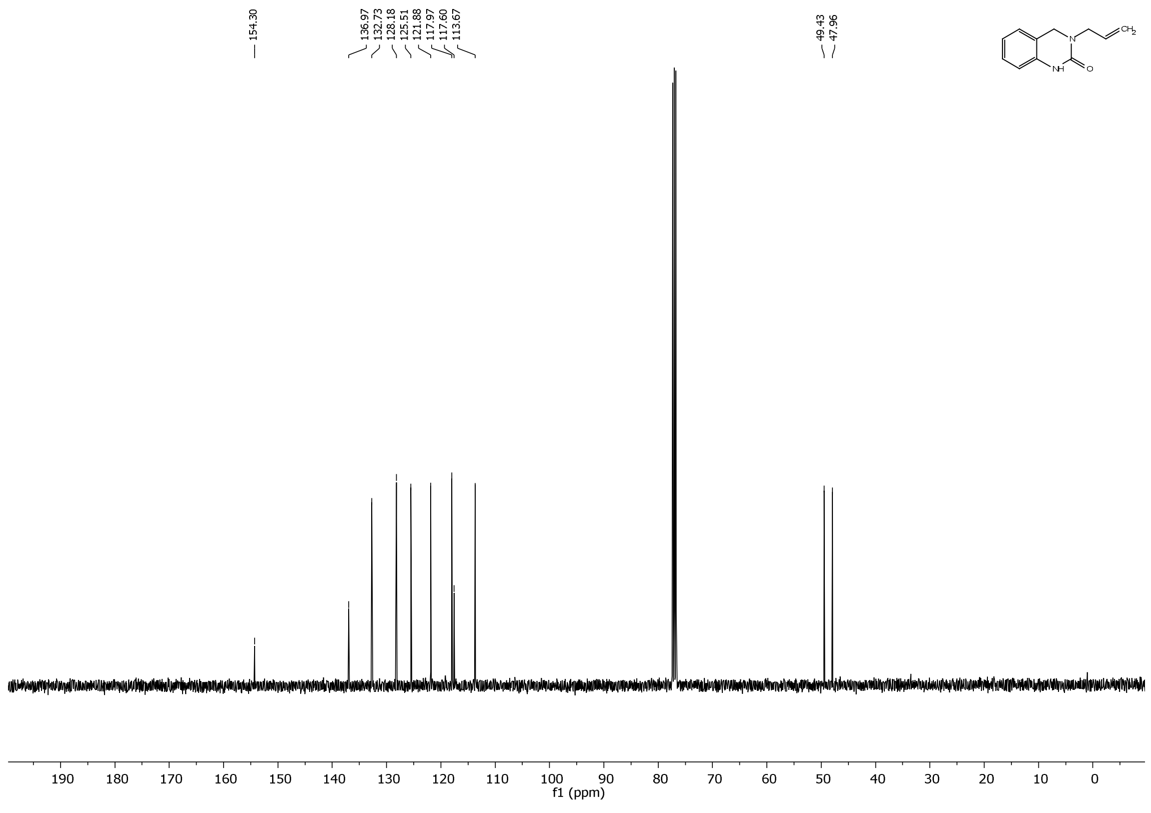

Compound 5d

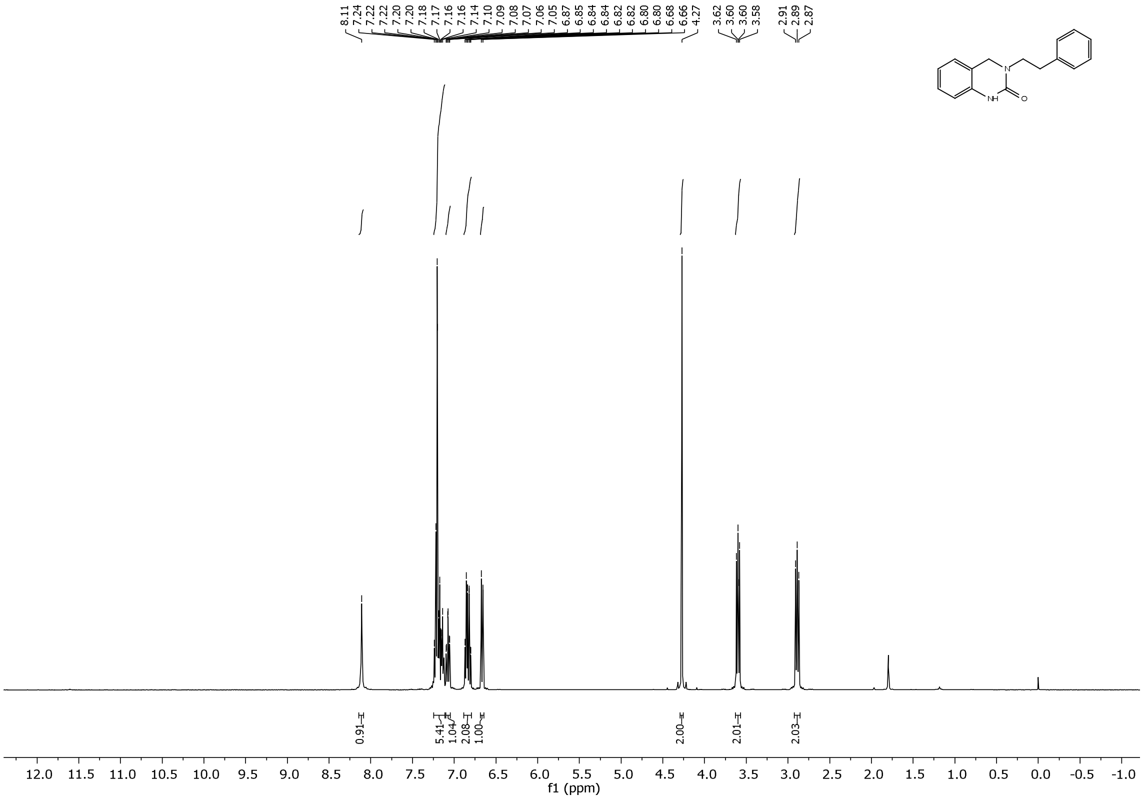

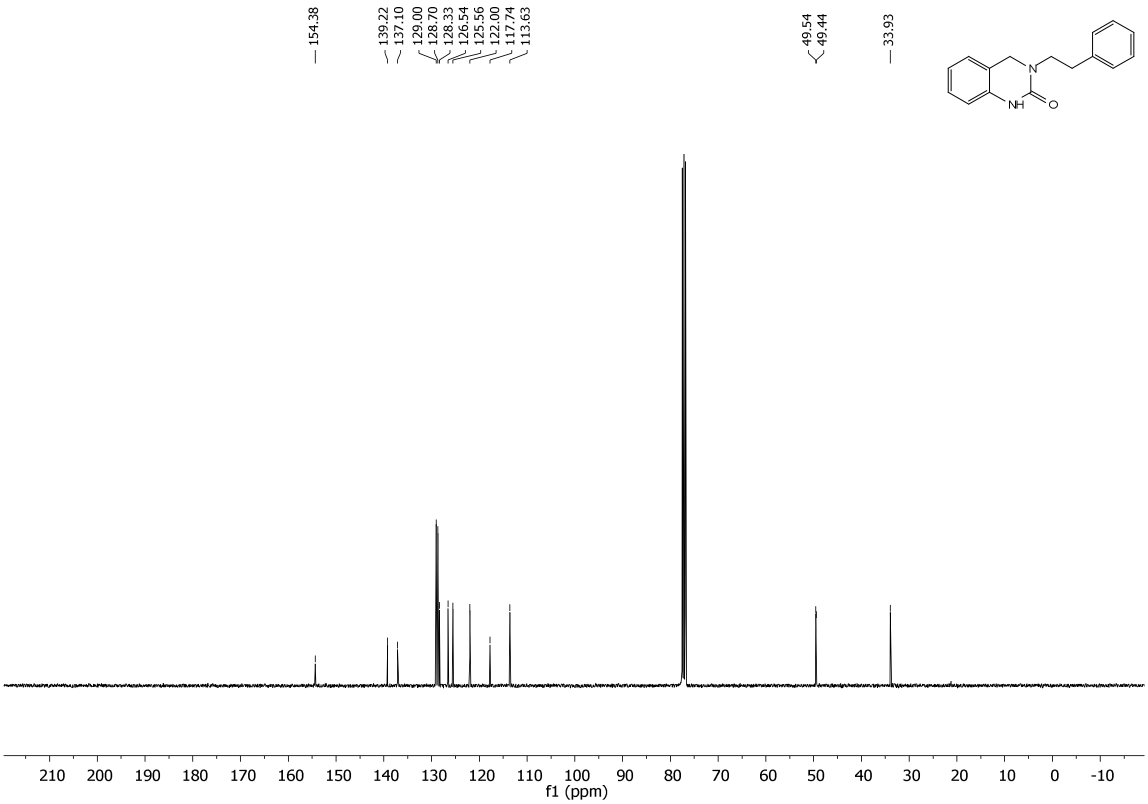

Compound 5e

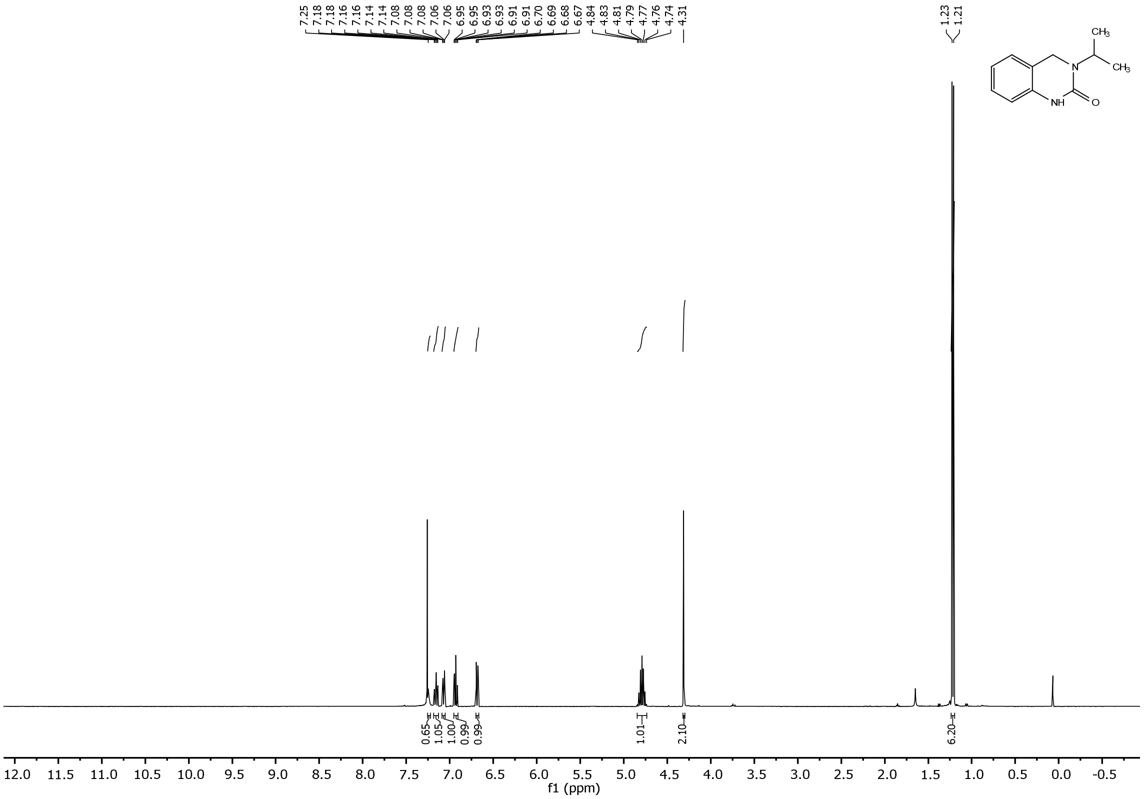

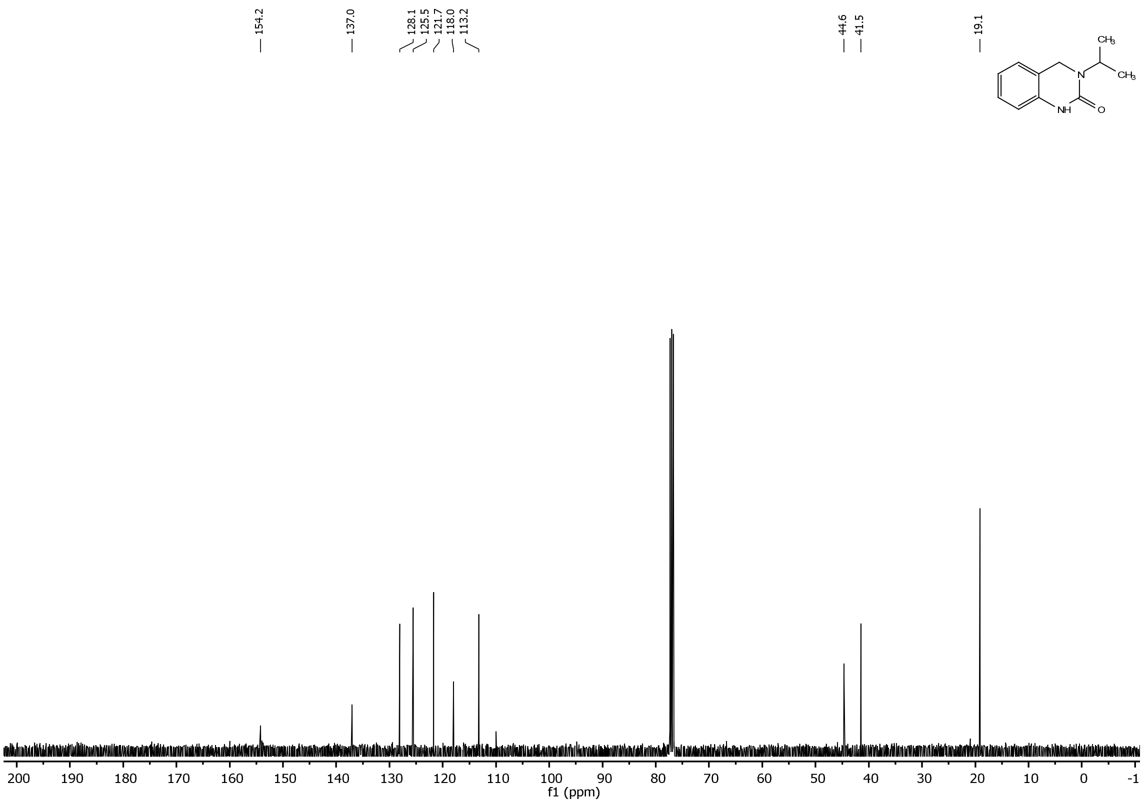

Compound 5f

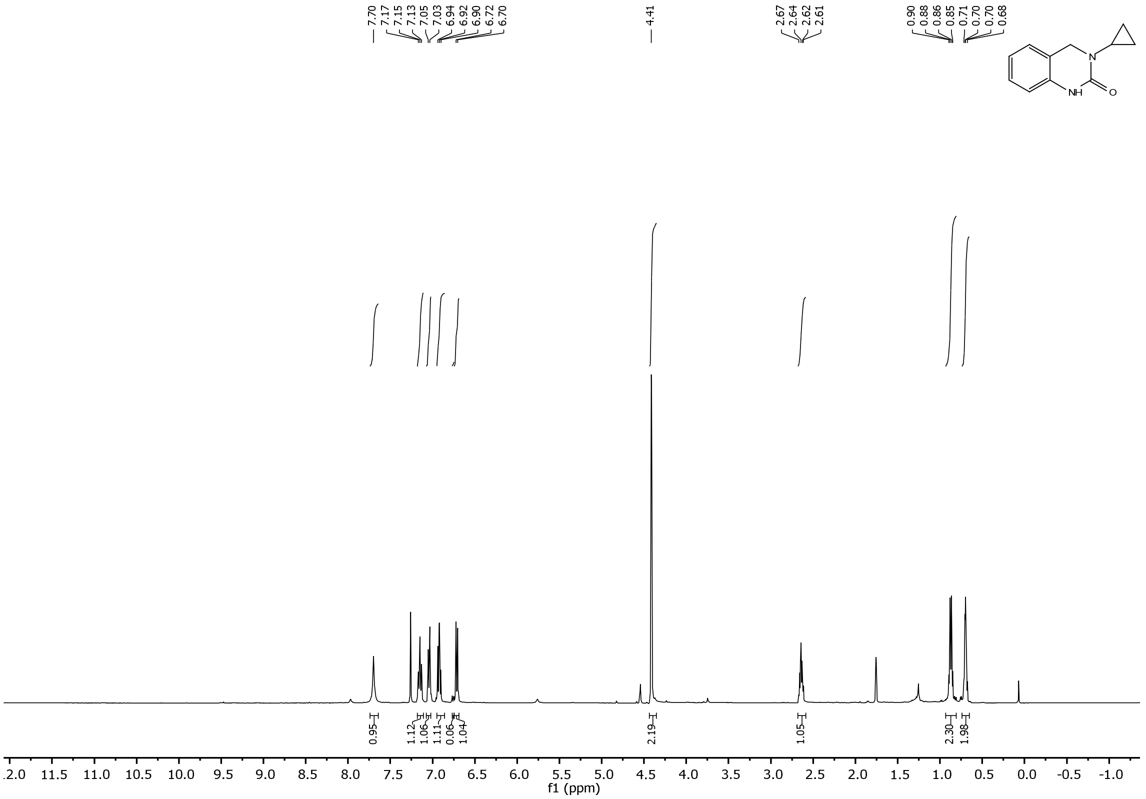
7.70
7.17
7.15
7.13
7.05
7.03
6.94
6.92
6.90
6.72
6.70
4.41
2.67
2.64
2.62
2.61
0.90
0.88
0.86
0.85
0.71
0.70
0.70
0.68
N
NH
O
0.95
1.12
1.06
1.11
0.06
1.04
2.19
1.05
2.30
1.98
f1 (ppm)

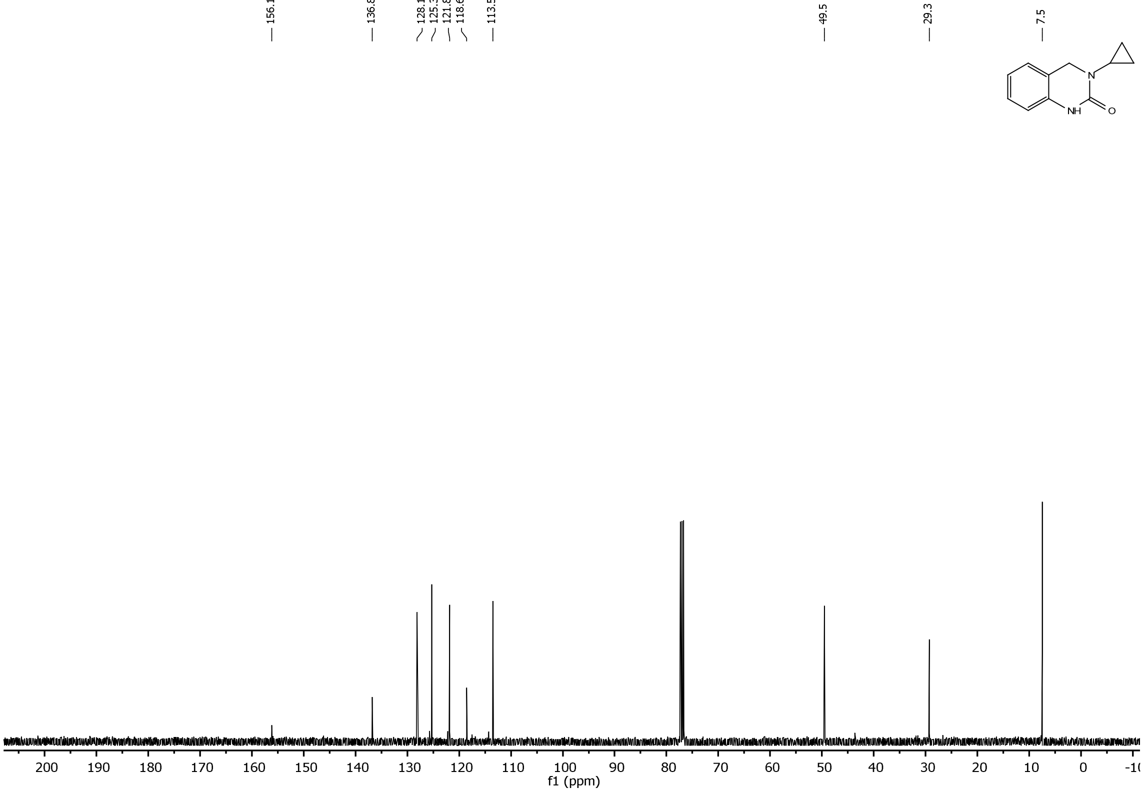
156.1
136.8
128.1
125.3
121.8
118.6
113.5
49.5
29.3
7.5
N
NH
O
f1 (ppm)

Compound 5g

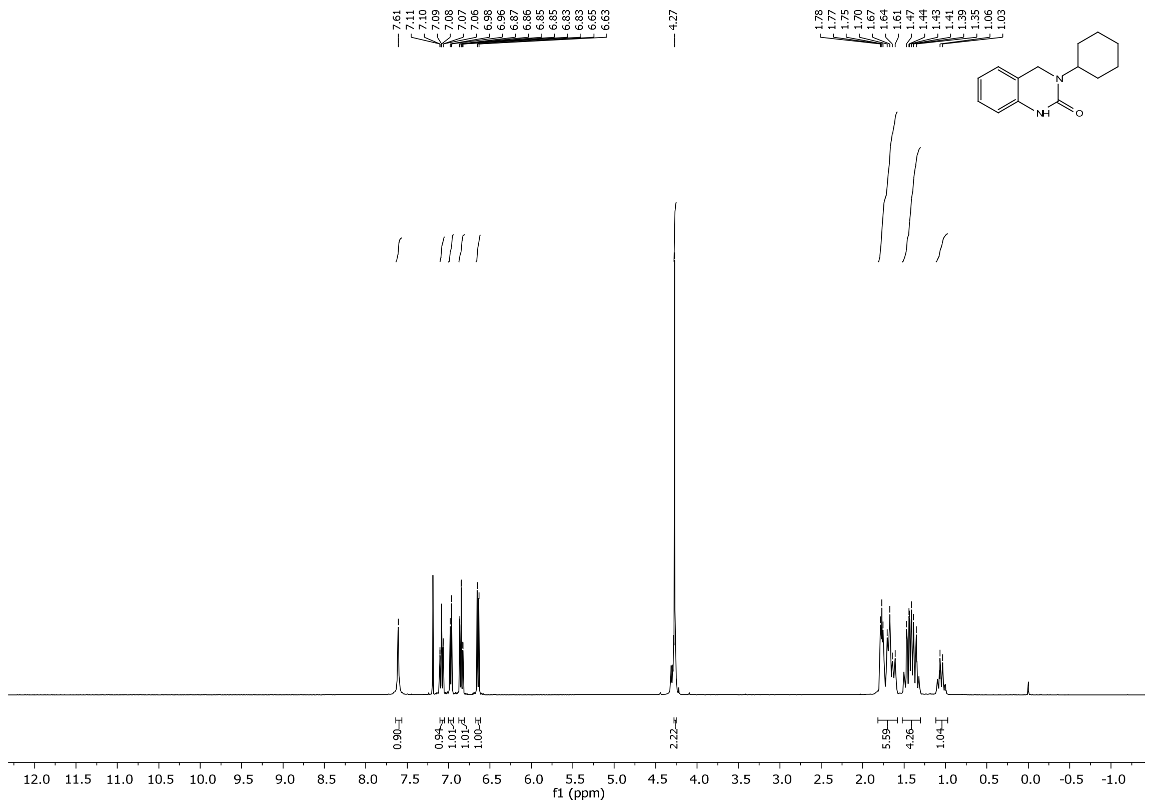
7.61
7.11
7.10
7.09
7.08
7.07
7.06
6.98
6.96
6.87
6.86
6.85
6.83
6.83
6.65
6.63
4.27
1.78
1.77
1.75
1.70
1.67
1.64
1.61
1.47
1.44
1.43
1.41
1.39
1.35
1.06
1.03
NH
N
O
0.90
0.94
1.01
1.01
1.00
2.22
5.59
4.26
1.04
f1 (ppm)

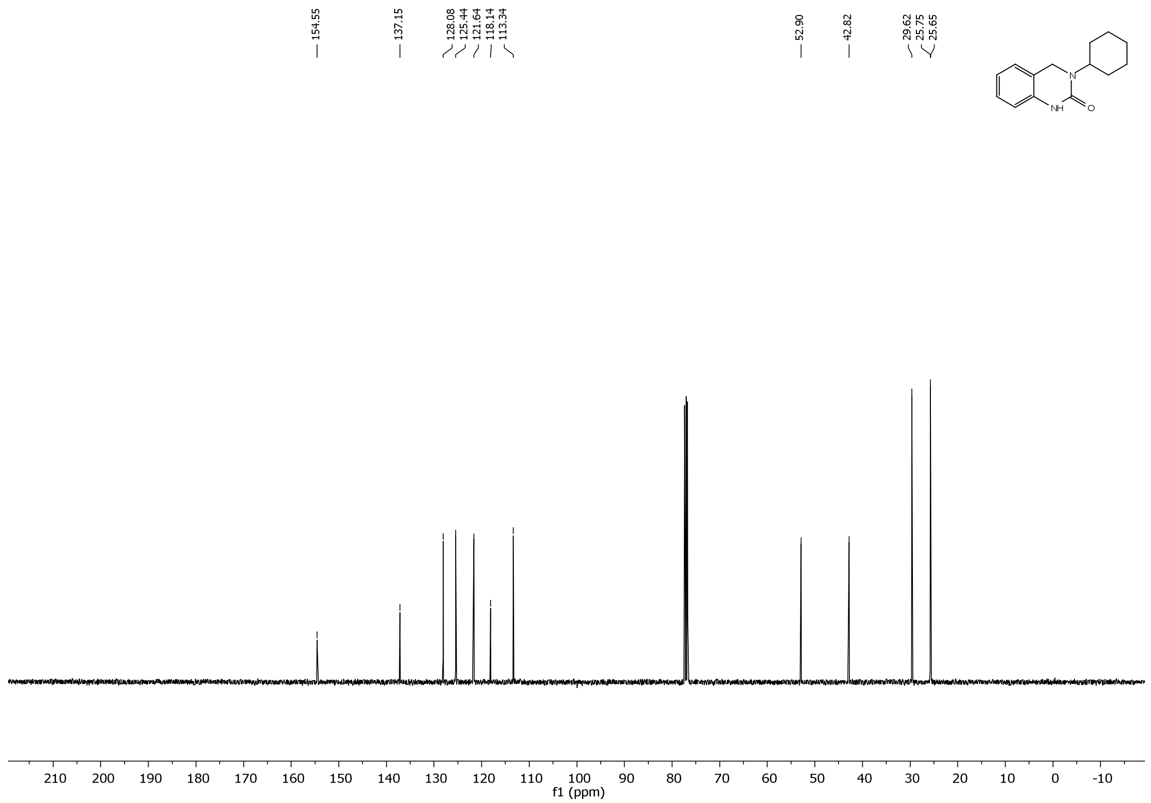
154.55
137.15
128.08
125.44
121.64
118.14
113.34
52.90
42.82
29.62
25.75
25.65
NH
N
O
f1 (ppm)

Compound 5h

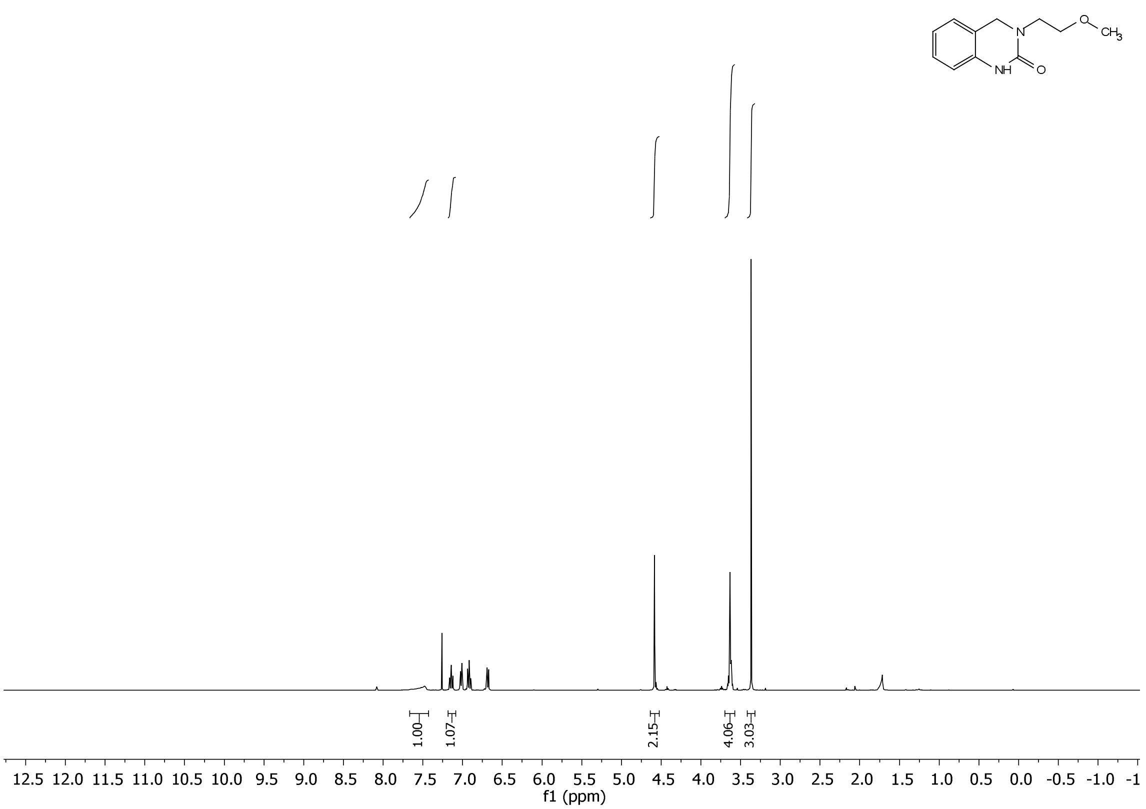

N
O
CH3
NH
O
1.00
1.07
2.15
4.06
3.03
12.5 12.0 11.5 11.0 10.5 10.0 9.5 9.0 8.5 8.0 7.5 7.0 6.5 6.0 5.5 5.0 4.5 4.0 3.5 3.0 2.5 2.0 1.5 1.0 0.5 0.0 -0.5 -1.0 -1
f1 (ppm)

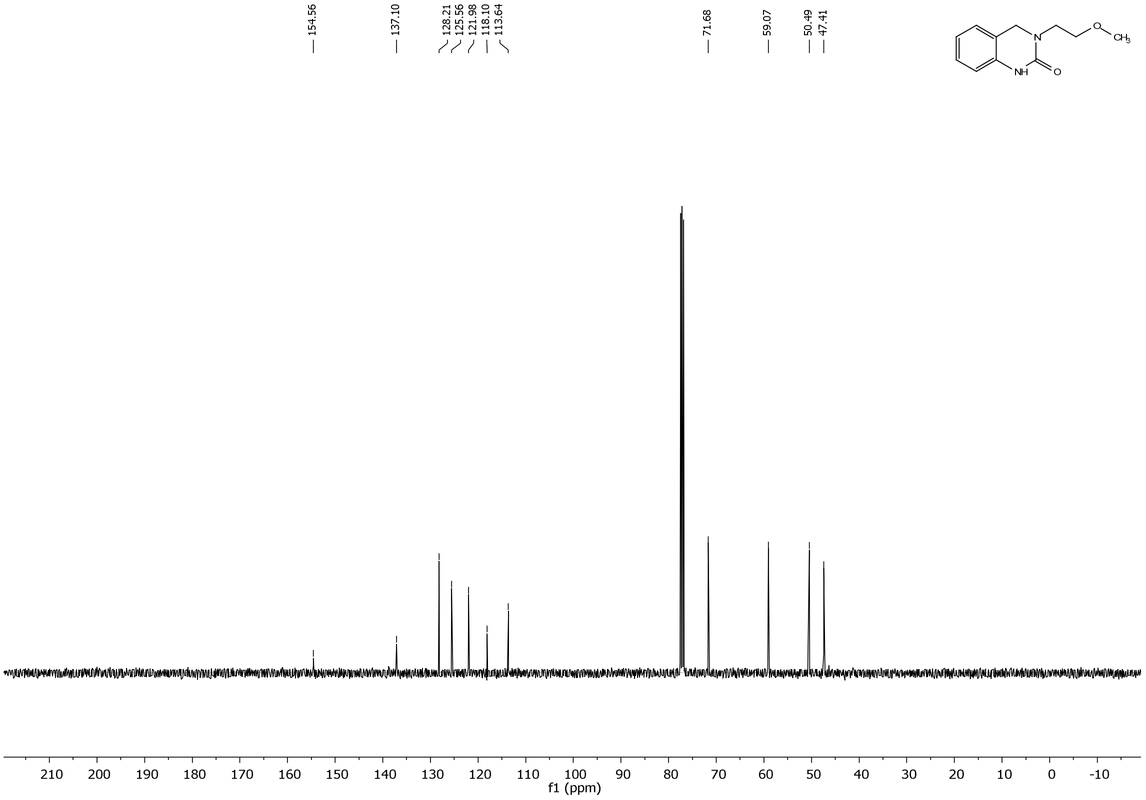

154.56
137.10
128.21
125.56
121.98
118.10
113.64
71.68
59.07
50.49
47.41
N
O
CH3
NH
O
210 200 190 180 170 160 150 140 130 120 110 100 90 80 70 60 50 40 30 20 10 0 -10
f1 (ppm)

Compound 5i

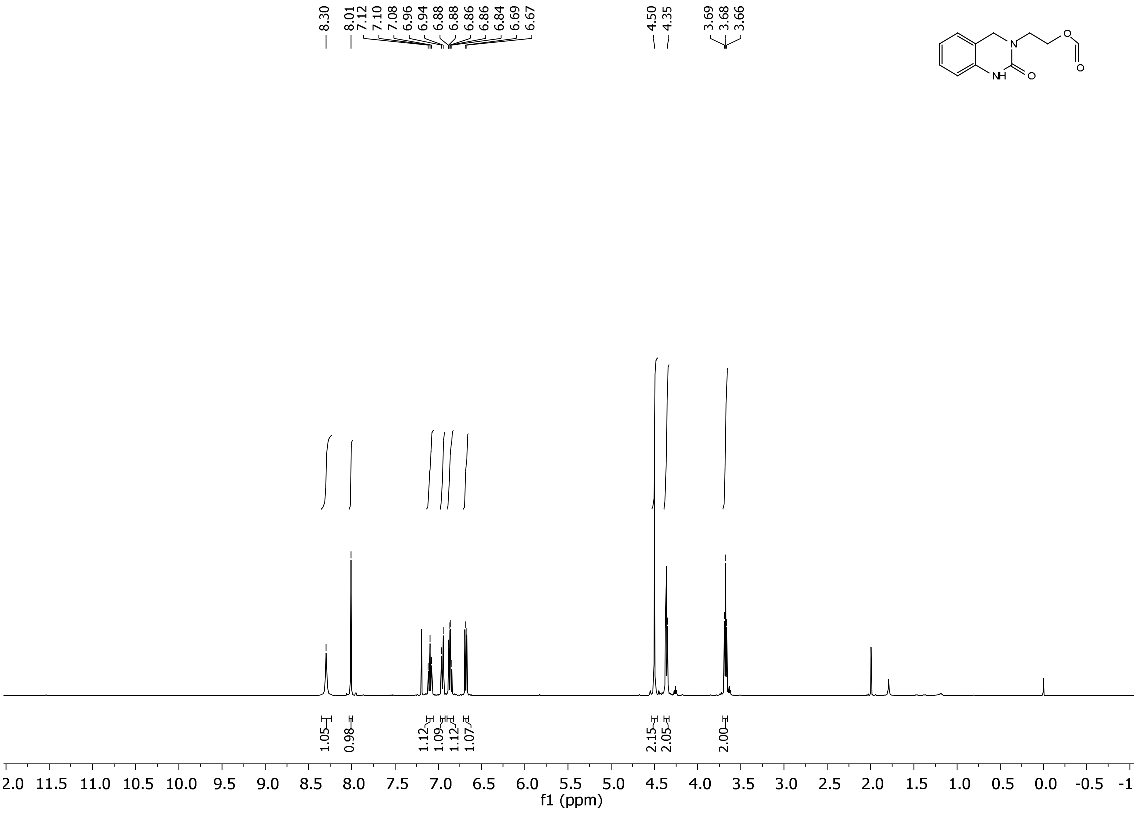
8.30
8.01
7.12
7.10
7.08
6.96
6.94
6.88
6.88
6.86
6.86
6.84
6.69
6.67
4.50
4.35
3.69
3.68
3.66
NH
N
O
O
O
1.05
0.98
1.12
1.09
1.12
1.07
2.15
2.05
2.00
f1 (ppm)

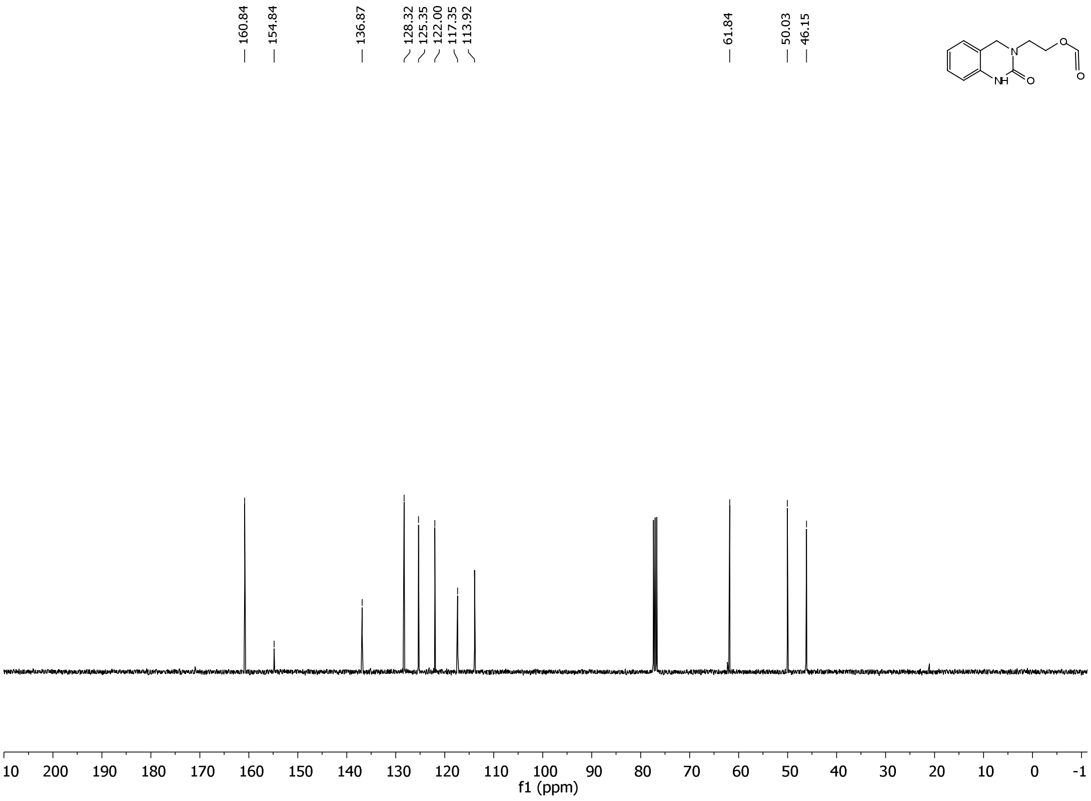
160.84
154.84
136.87
128.32
125.35
122.00
117.35
113.92
61.84
50.03
46.15
NH
N
O
O
O
f1 (ppm)

Compound 5j

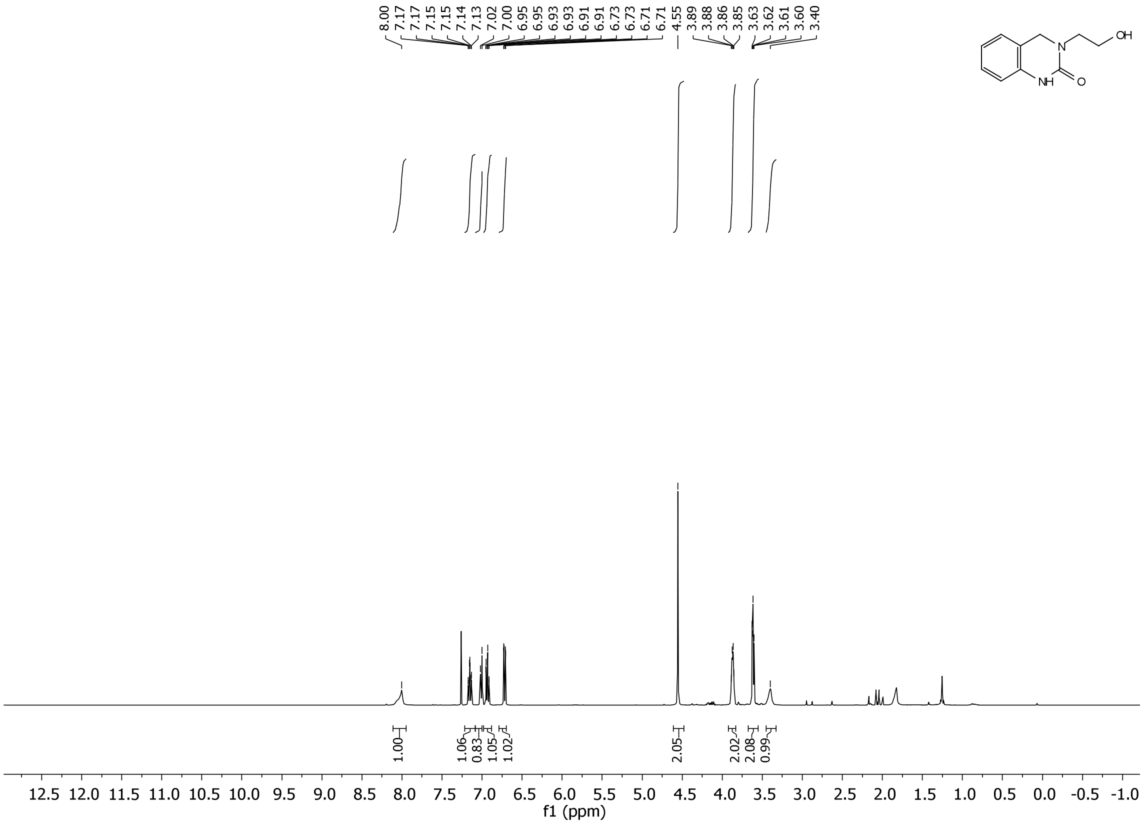

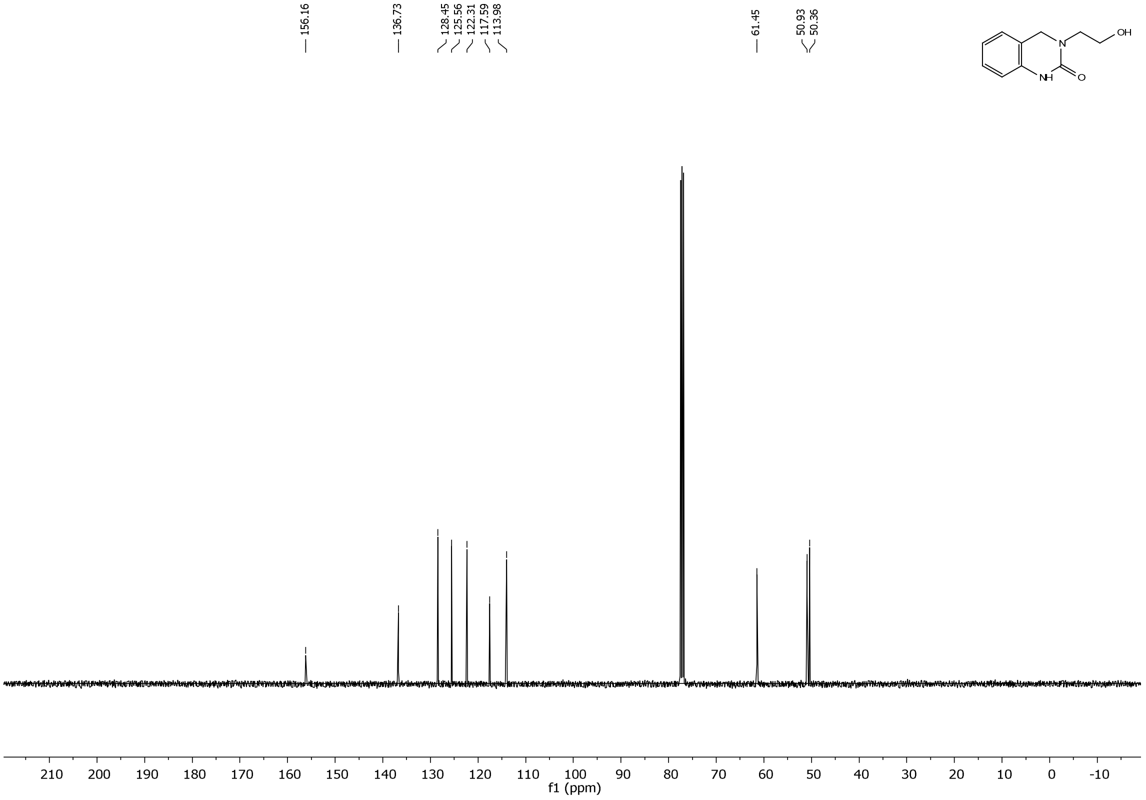

Compound 5k

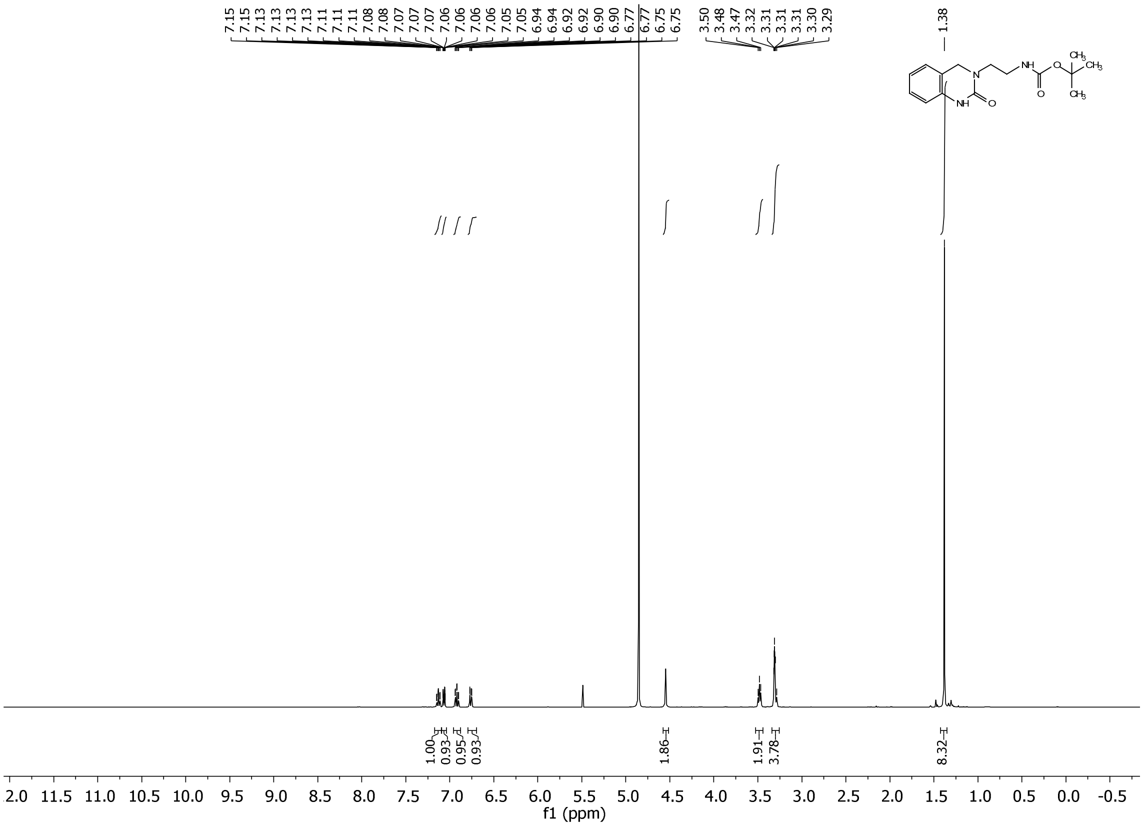

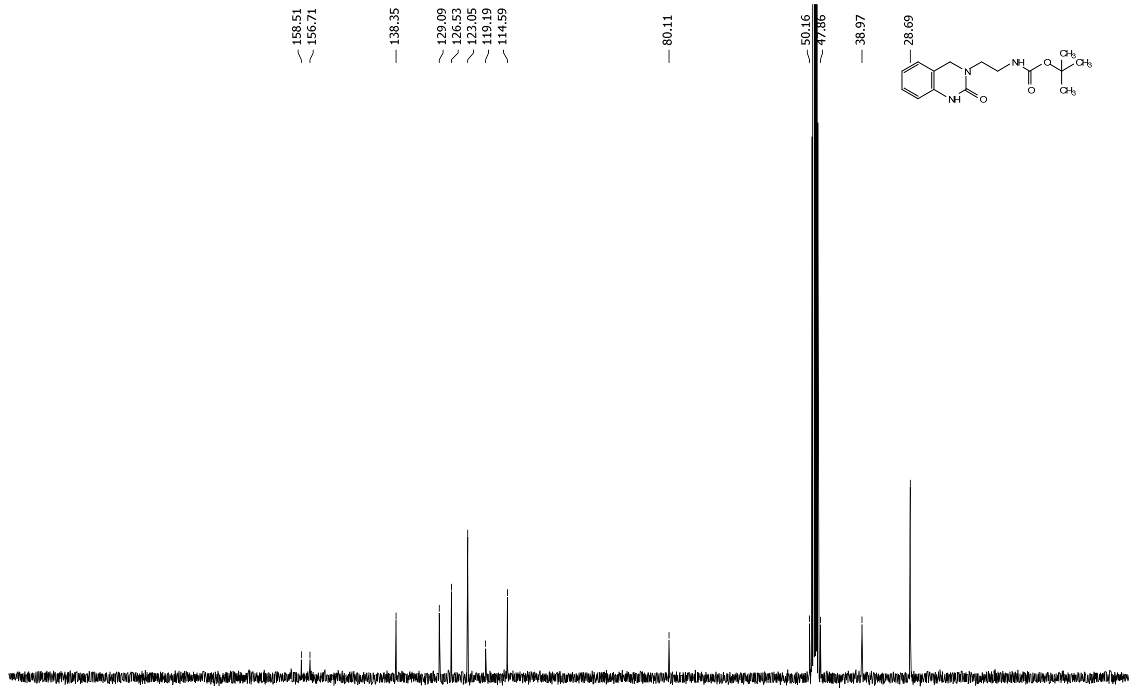

Compound 5l

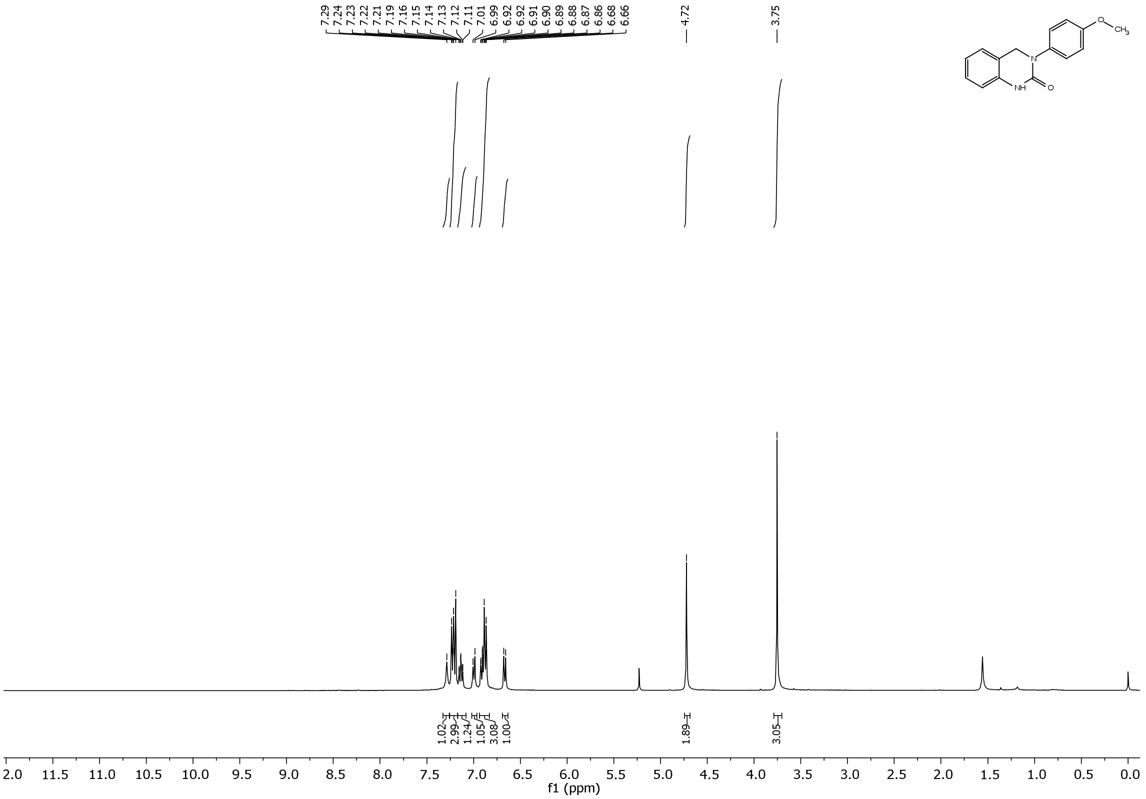
7.29 7.24 7.23 7.22 7.21 7.19 7.16 7.15 7.14 7.13 7.12 7.11 7.01 6.99 6.92 6.92 6.91 6.90 6.89 6.88 6.87 6.86 6.68 6.66
4.72
3.75
1.02 2.99 1.24 1.05 3.08 1.00
1.89
3.05
f1 (ppm)

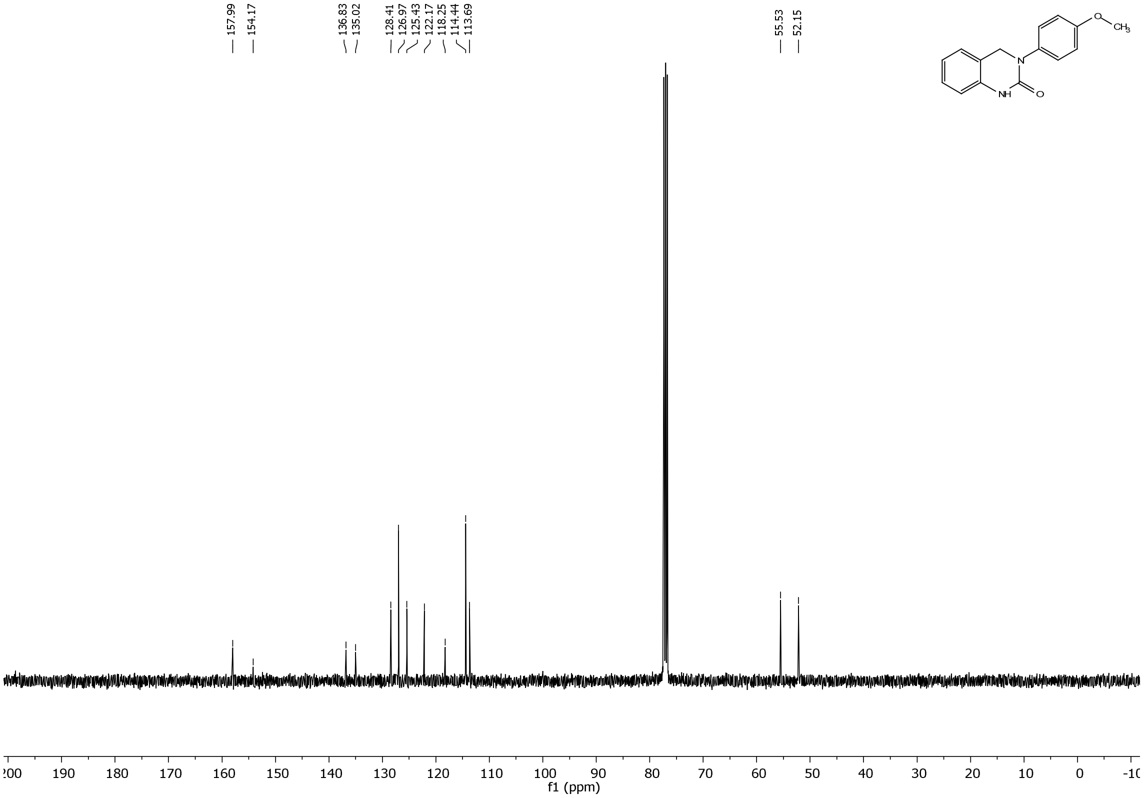
157.99
154.17
136.83
135.02
128.41
126.97
125.43
122.17
118.25
114.44
113.69
55.53
52.15
f1 (ppm)

Compound 5m

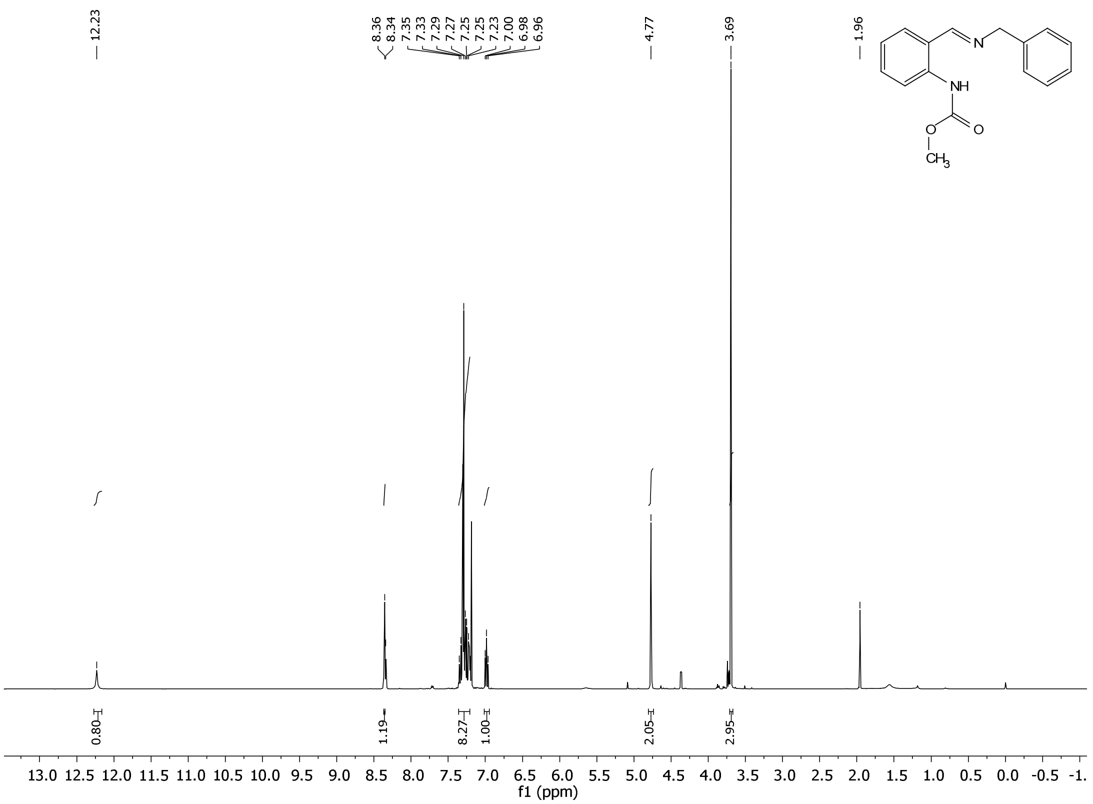
12.23
8.36
8.34
7.35
7.33
7.29
7.27
7.25
7.25
7.23
7.00
6.98
6.96
4.77
3.69
1.96
NH
N
O
O
CH3
0.80
1.19
8.27
1.00
2.05
2.95
13.0 12.5 12.0 11.5 11.0 10.5 10.0 9.5 9.0 8.5 8.0 7.5 7.0 6.5 6.0 5.5 5.0 4.5 4.0 3.5 3.0 2.5 2.0 1.5 1.0 0.5 0.0 -0.5 -1.
f1 (ppm)

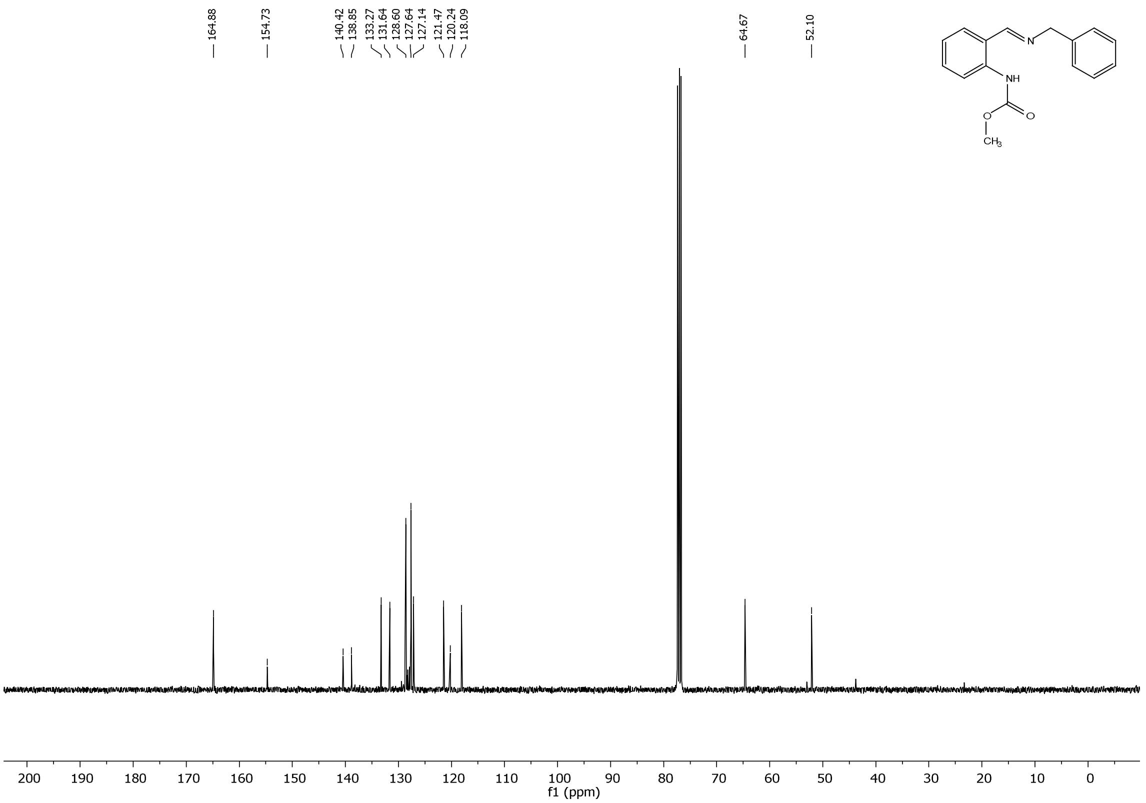
164.88
154.73
140.42
138.85
133.27
131.64
128.60
127.64
127.14
121.47
120.24
118.09
64.67
52.10
NH
N
O
O
CH3
200 190 180 170 160 150 140 130 120 110 100 90 80 70 60 50 40 30 20 10 0
f1 (ppm)

Compound 7

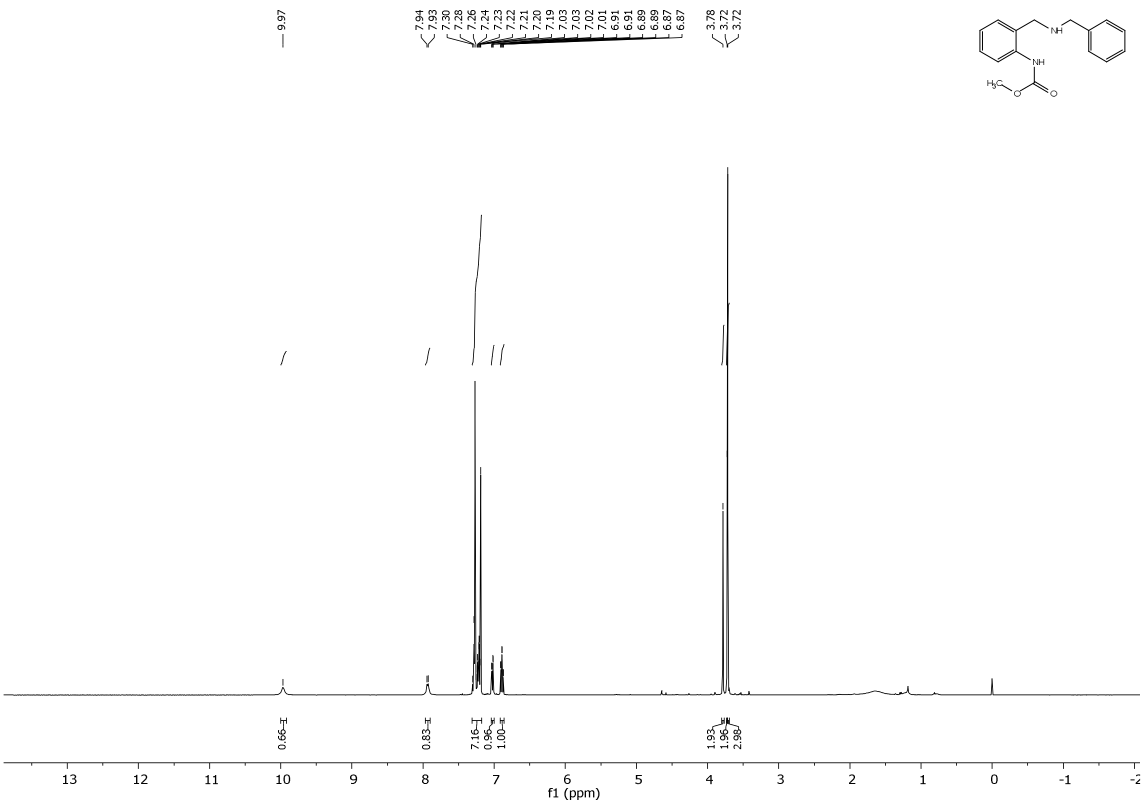

9.97
7.94
7.93
7.30
7.28
7.26
7.24
7.23
7.22
7.21
7.20
7.19
7.03
7.03
7.02
7.01
6.91
6.91
6.89
6.89
6.87
6.87
3.78
3.72
3.72
NH
H_3C
O
0.66
0.83
7.16
0.96
1.00
1.93
1.96
2.98
f1 (ppm)

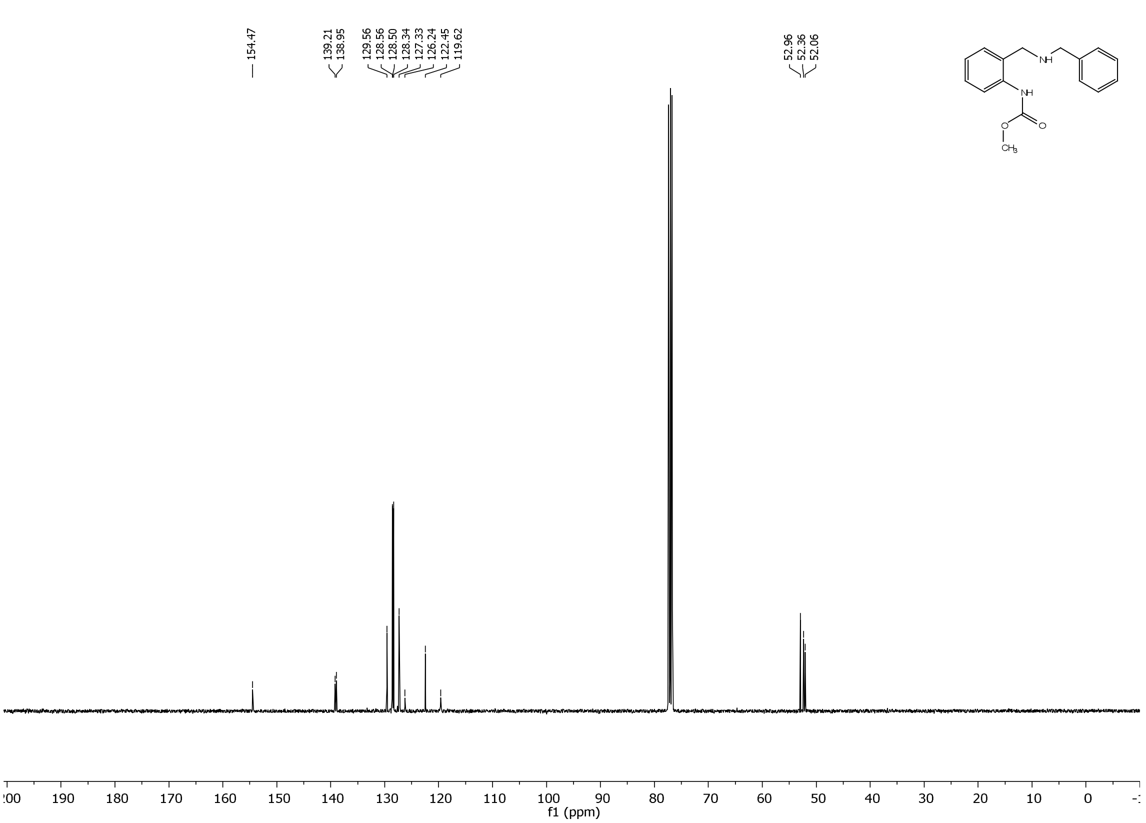

154.47
139.21
138.95
129.56
128.56
128.50
128.34
127.33
126.24
122.45
119.62
52.96
52.36
52.06
NH
O
CH_3
f1 (ppm)

Compound 8

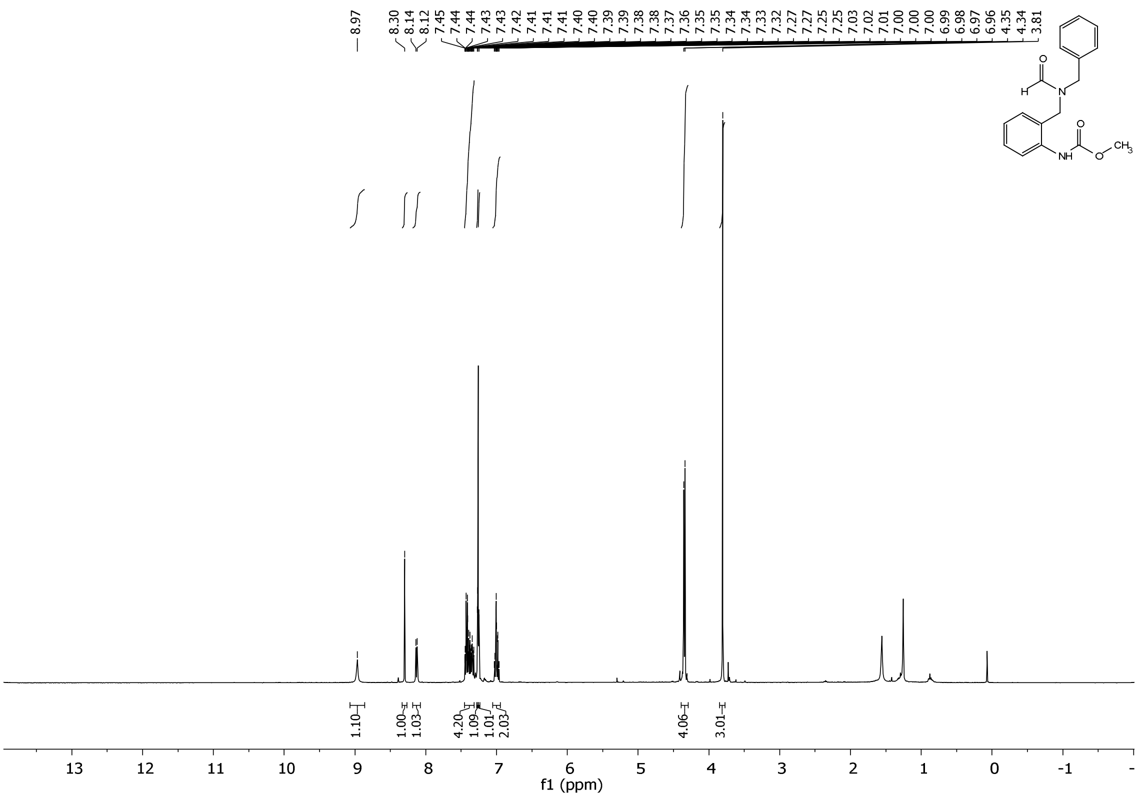
8.97
8.30
8.14
8.12
7.45
7.44
7.44
7.43
7.43
7.42
7.41
7.41
7.40
7.40
7.39
7.39
7.38
7.38
7.37
7.36
7.35
7.35
7.34
7.34
7.33
7.32
7.27
7.27
7.25
7.25
7.03
7.02
7.01
7.00
7.00
7.00
6.99
6.98
6.97
6.96
4.35
4.34
3.81
O
H
N
NH
O
O
CH3
1.10
1.00
1.03
4.20
1.09
1.01
2.03
4.06
3.01
f1 (ppm)

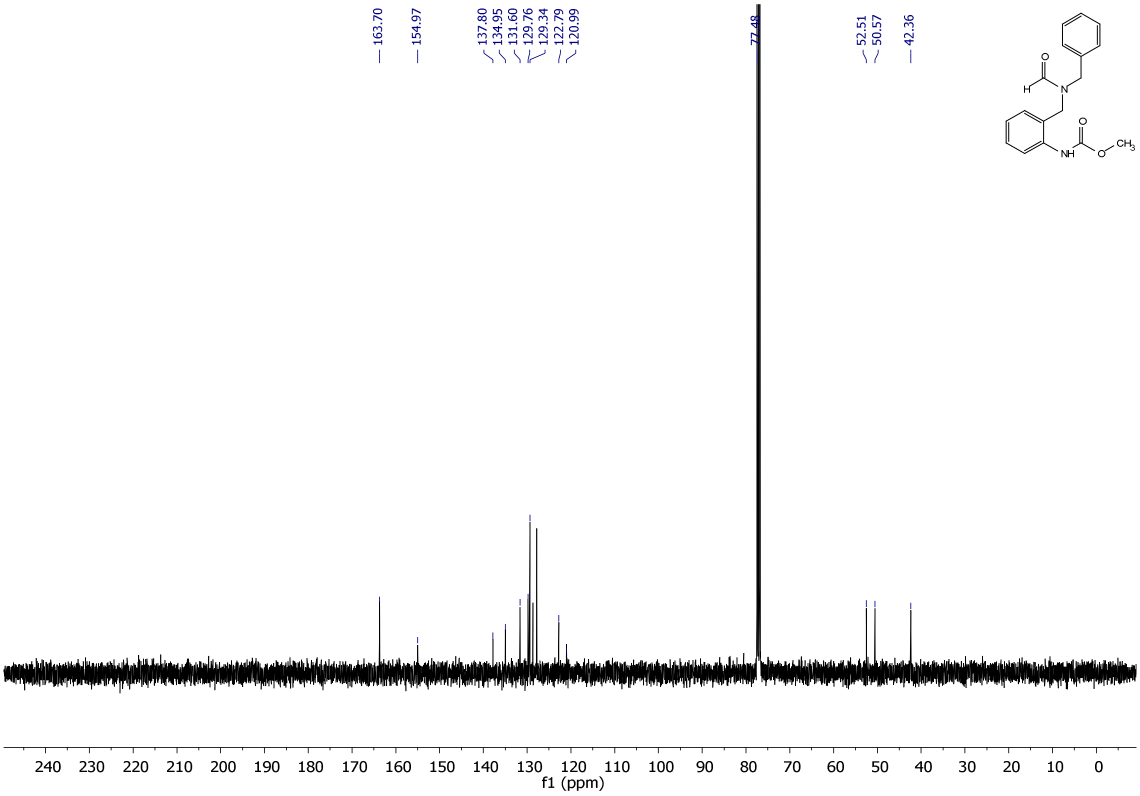
163.70
154.97
137.80
134.95
131.60
129.76
129.34
122.79
120.99
77.48
52.51
50.57
42.36
O
H
N
NH
O
O
CH3
f1 (ppm)

Compound 8a

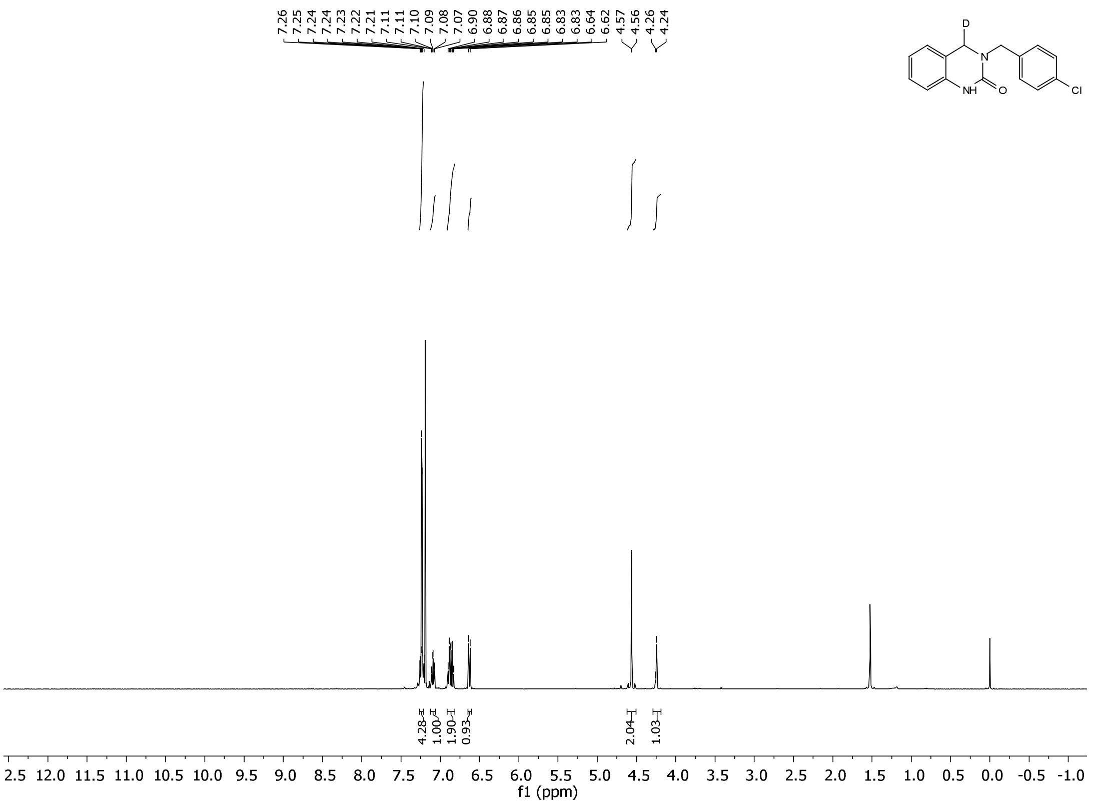
7.26
7.25
7.24
7.24
7.23
7.22
7.21
7.11
7.11
7.10
7.09
7.08
7.07
6.90
6.88
6.87
6.86
6.85
6.85
6.83
6.83
6.64
6.62
4.57
4.56
4.26
4.24
D
N
NH
O
Cl
4.28
1.00
1.90
0.93
2.04
1.03
2.5 12.0 11.5 11.0 10.5 10.0 9.5 9.0 8.5 8.0 7.5 7.0 6.5 6.0 5.5 5.0 4.5 4.0 3.5 3.0 2.5 2.0 1.5 1.0 0.5 0.0 -0.5 -1.0
f1 (ppm)

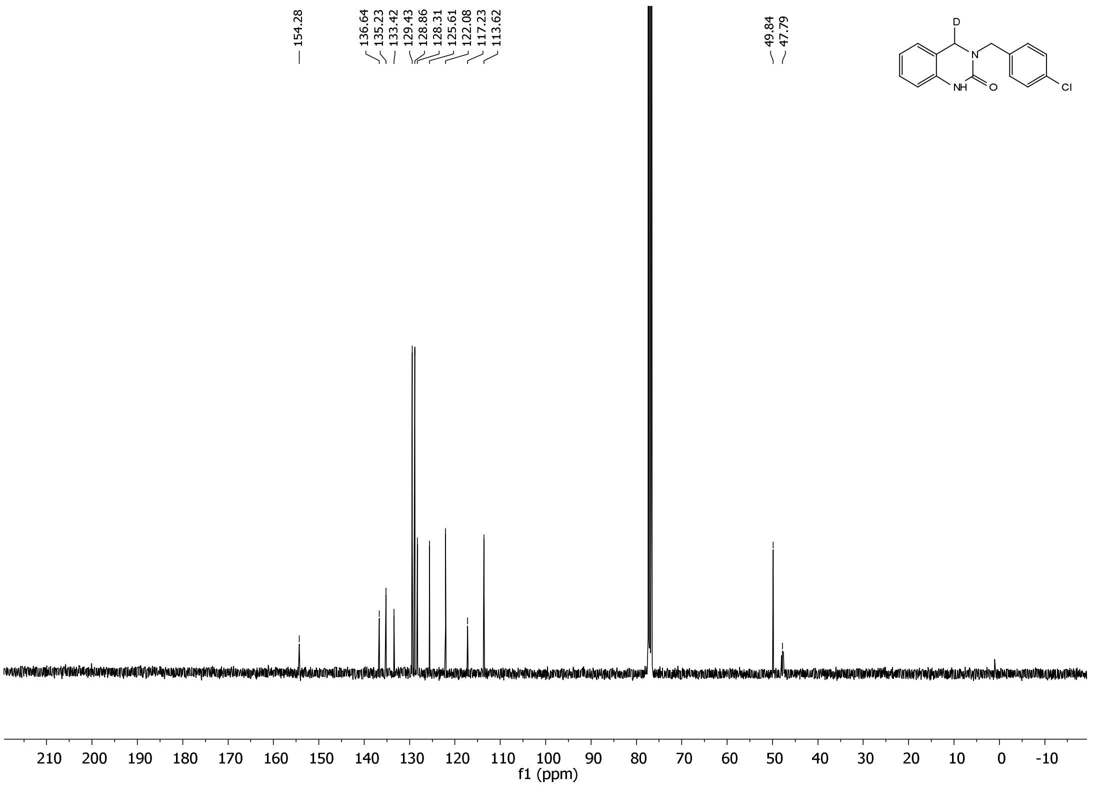
154.28
136.64
135.23
133.42
129.43
128.86
128.31
125.61
122.08
117.23
113.62
49.84
47.79
D
N
NH
O
Cl
210 200 190 180 170 160 150 140 130 120 110 100 90 80 70 60 50 40 30 20 10 0 -10
f1 (ppm)

Compound 9

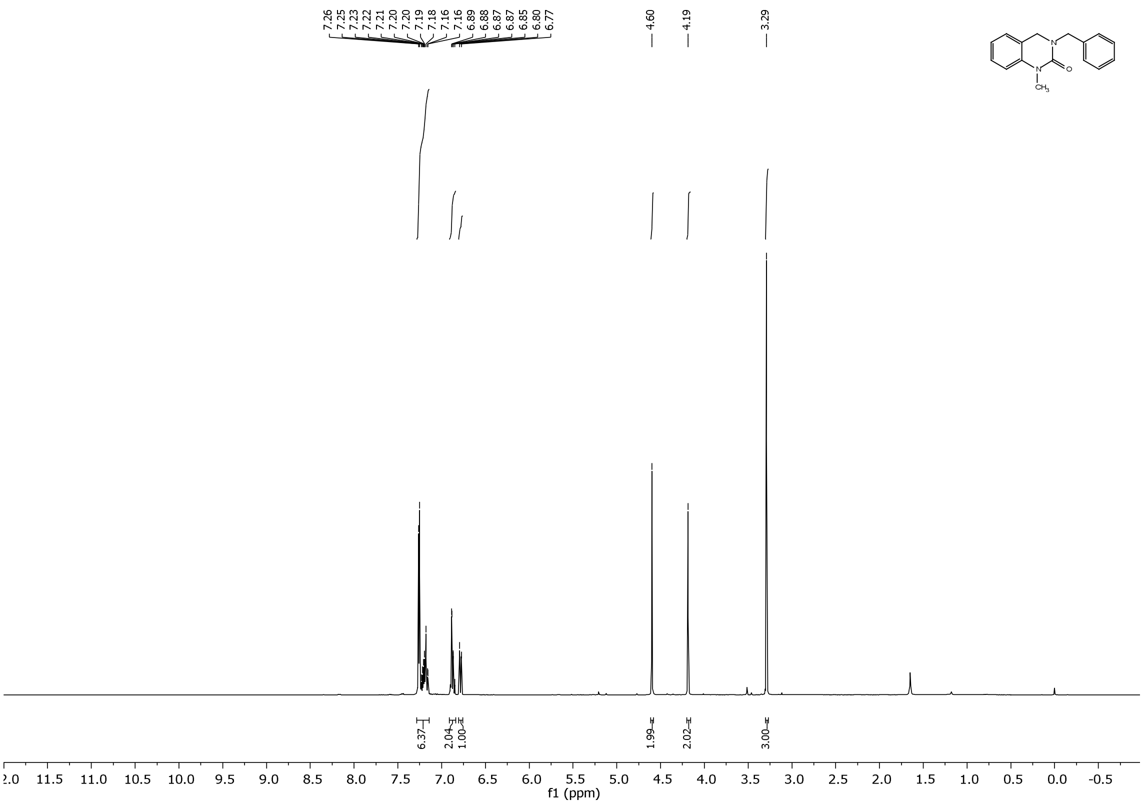

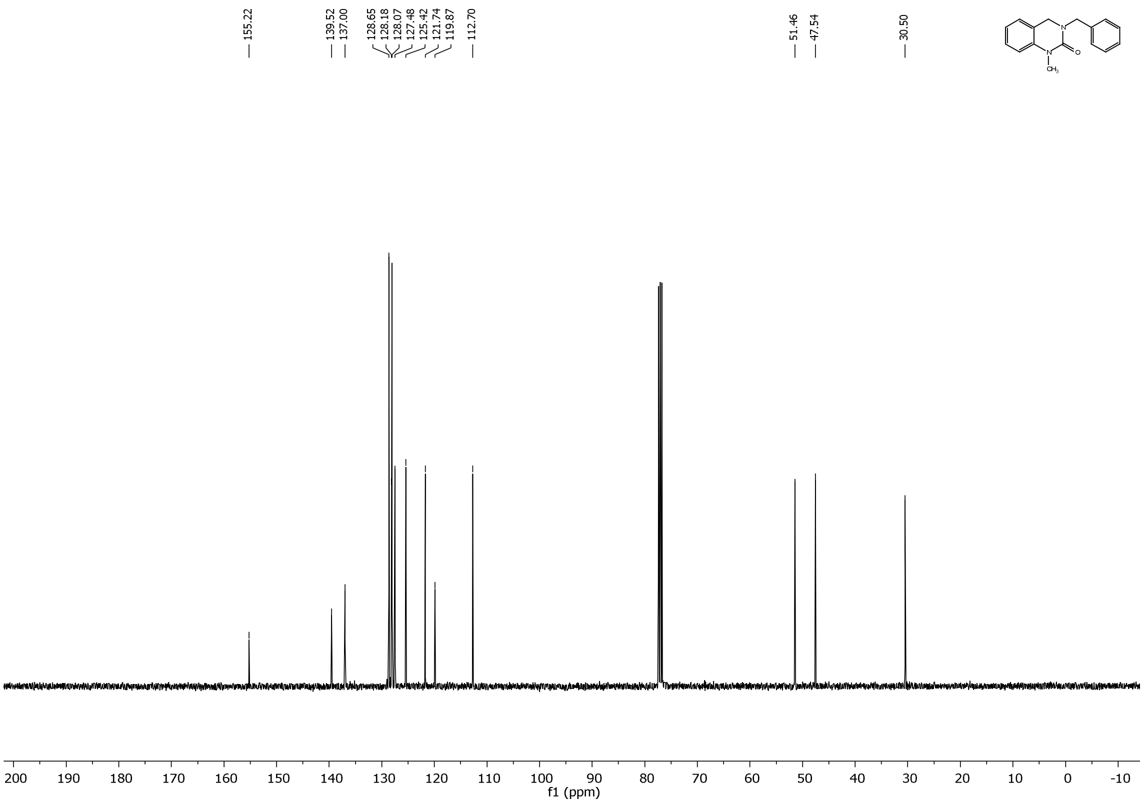

Compound 10a

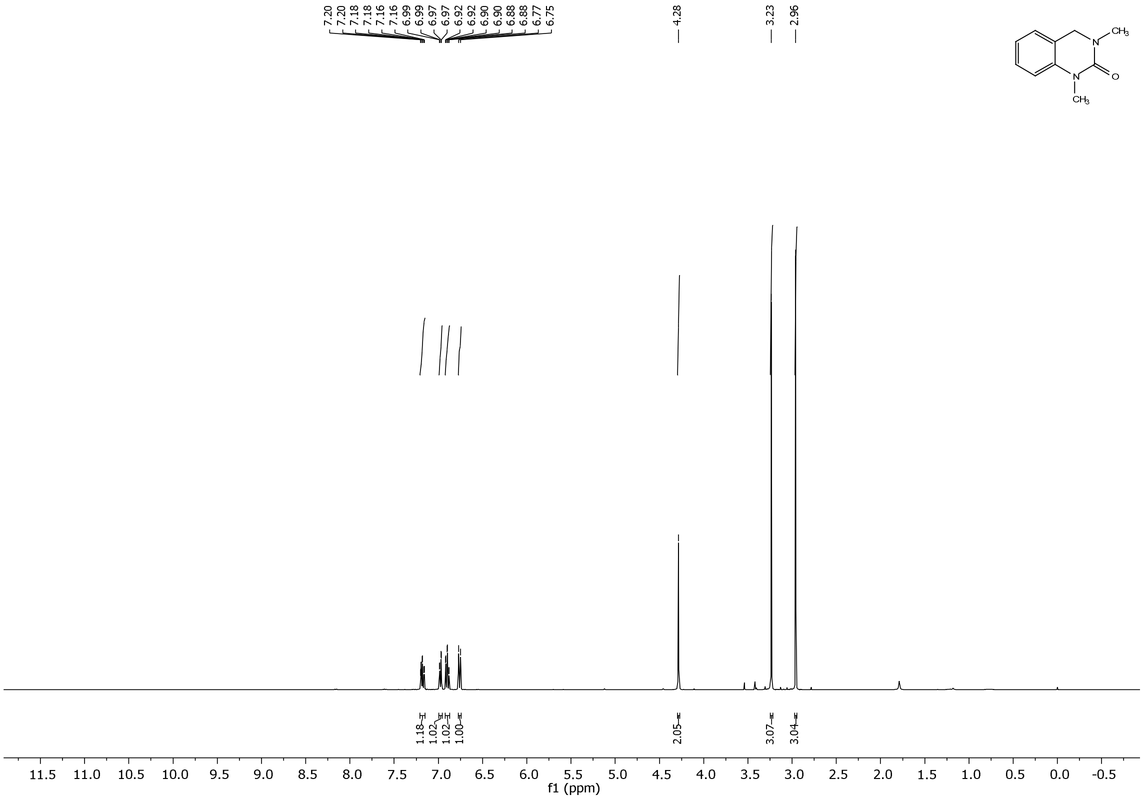

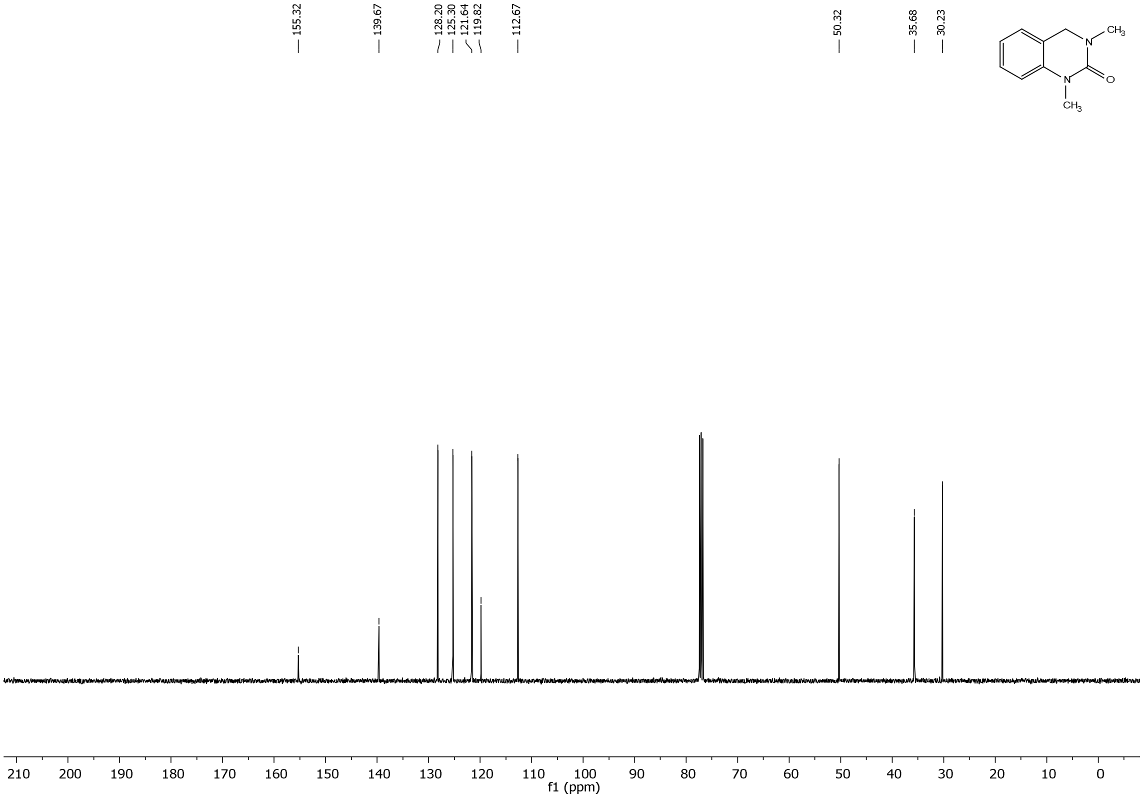

Compound 10b

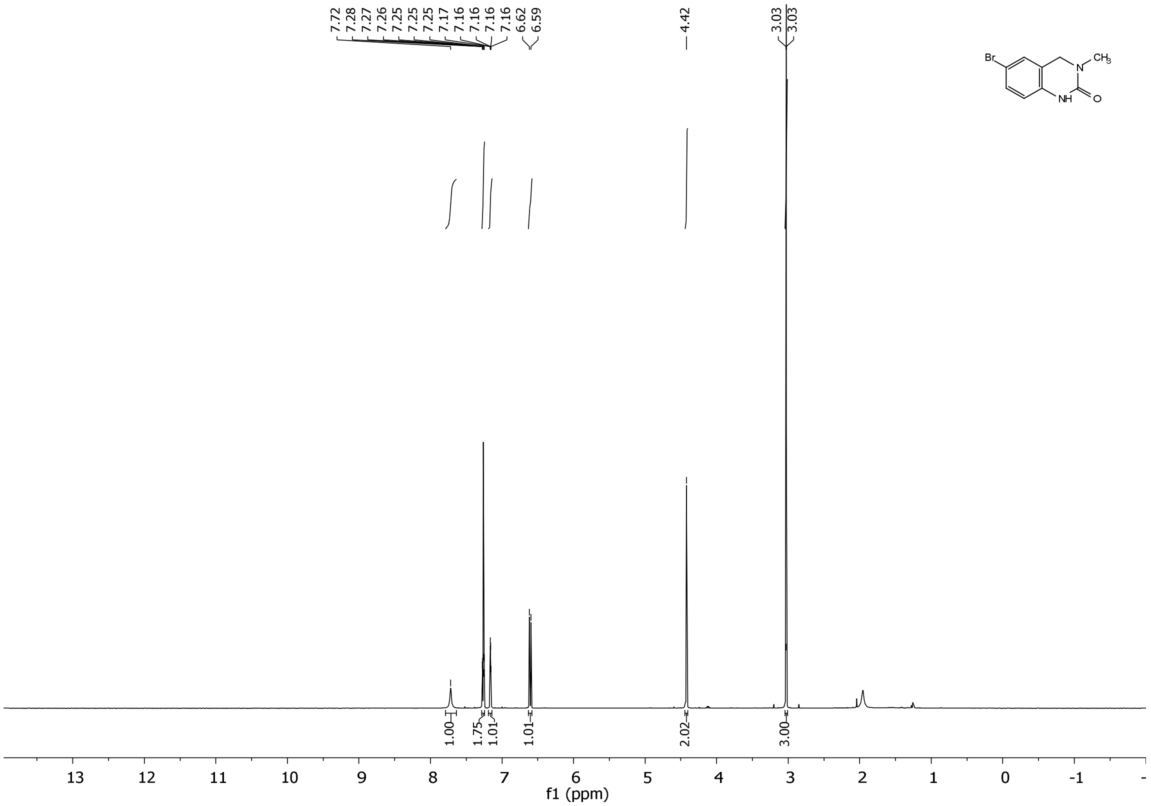
7.72
7.28
7.27
7.26
7.25
7.25
7.25
7.17
7.16
7.16
7.16
7.16
6.62
6.59
4.42
3.03
3.03
1.00
1.75
1.01
1.01
2.02
3.00
f1 (ppm)

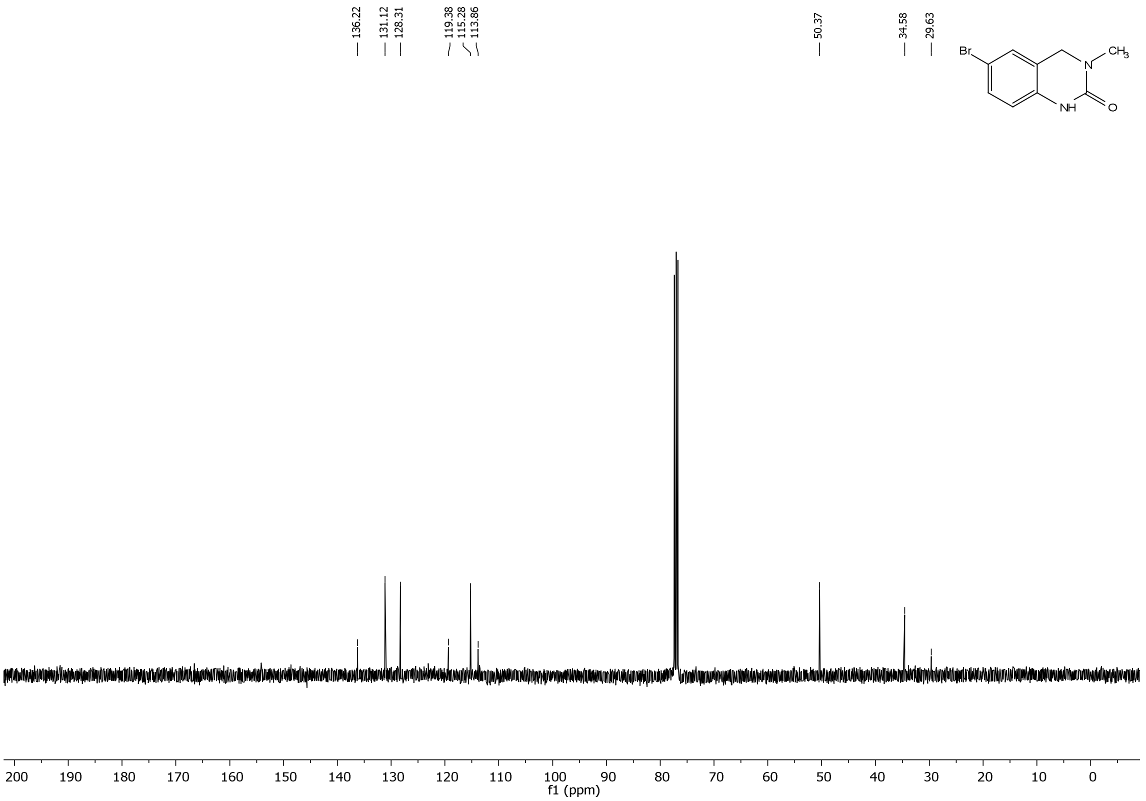
136.22
131.12
128.31
119.38
115.28
113.86
50.37
34.58
29.63
f1 (ppm)

Compound 11

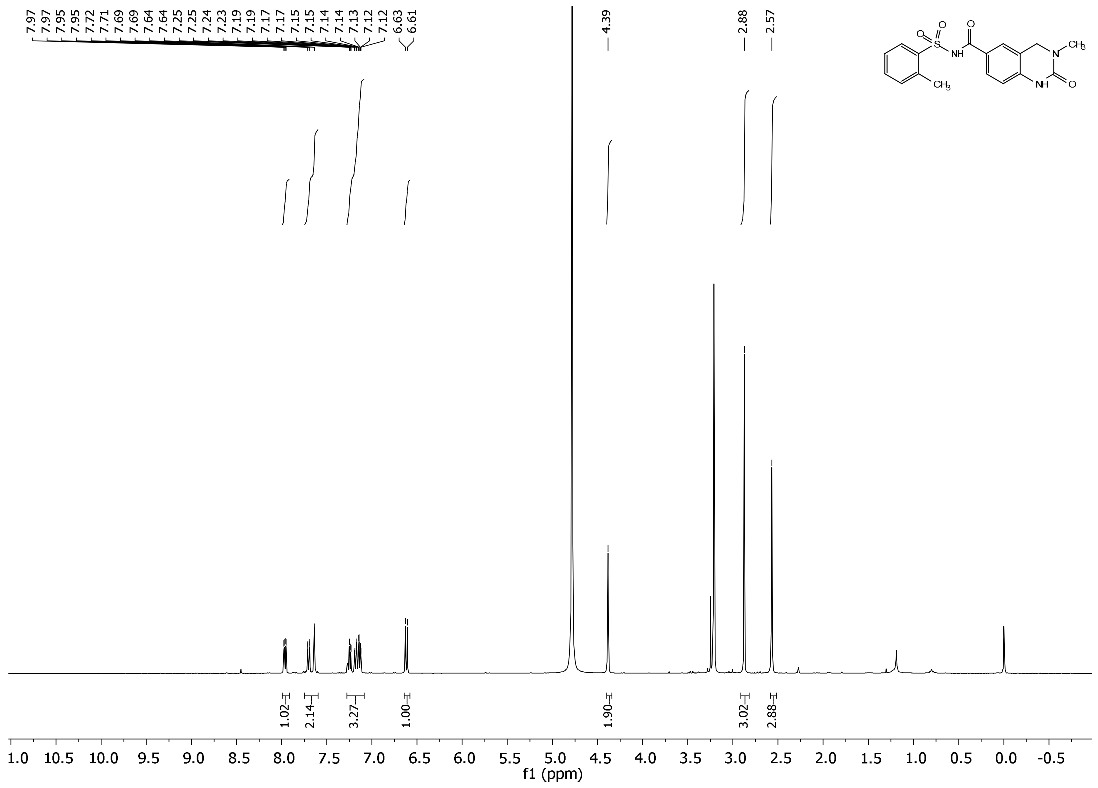

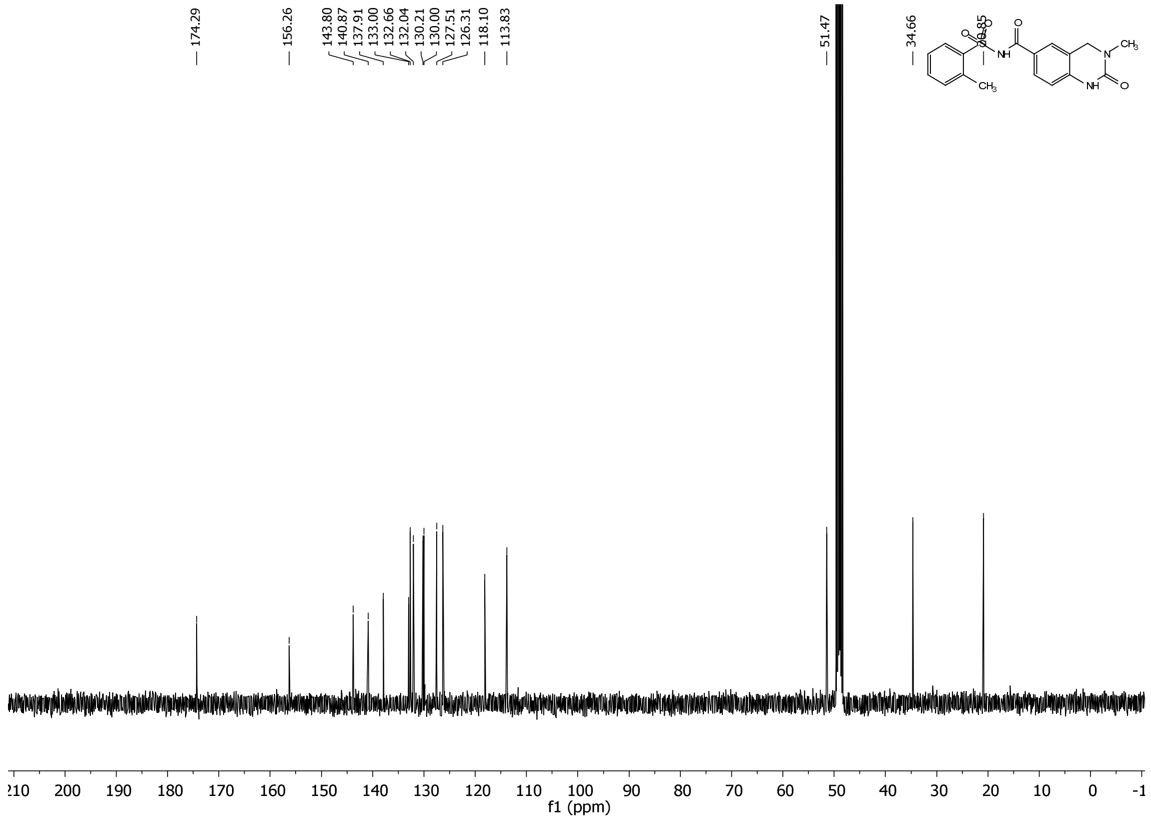

Compound 13

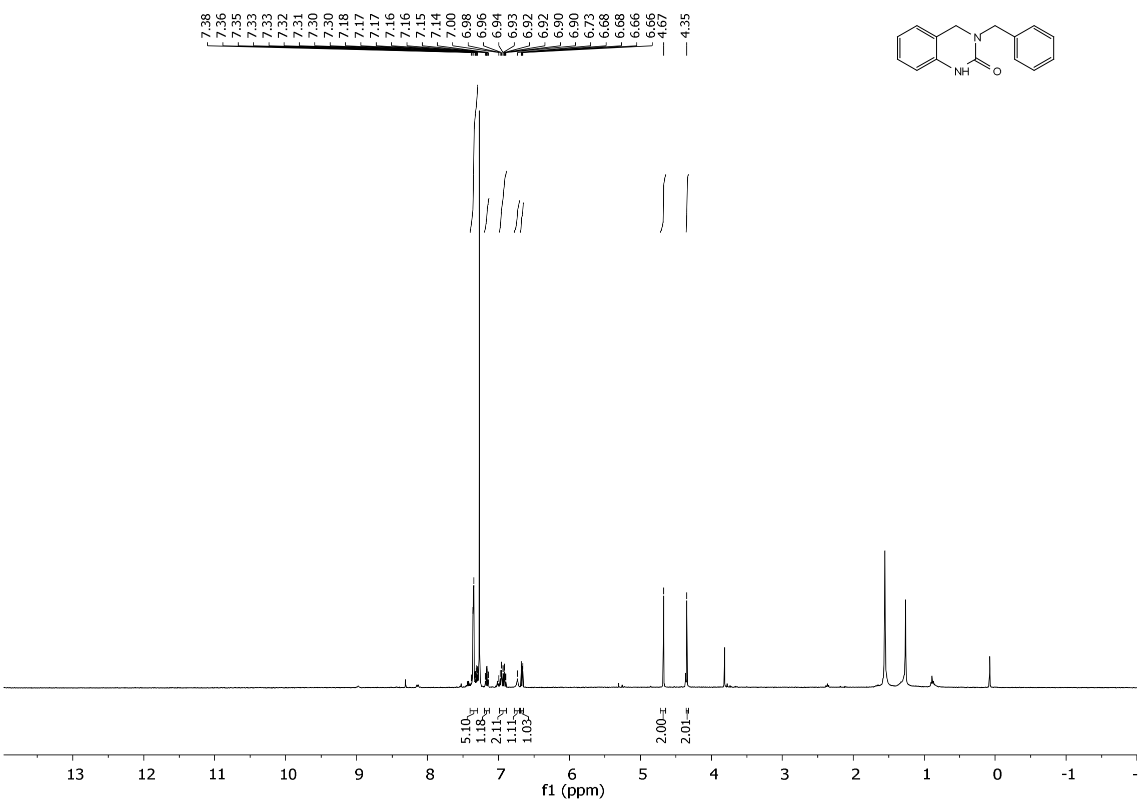

Compound 4a. Isolated from the attempted cyclization of **8**

References

1 M. Y. Stevens, K. Wieckowski, P. Wu, R. T. Sawant and L. R. Odell, *Org. Biomol. Chem.*, 2015, **13**, 2044–54.

2 R. T. Sawant, M. Y. Stevens and L. R. Odell, *Eur J. Org. Chem.*, 2015, 7743–7755.

3 M. E. Camacho, M. Chayah, M. E. García, N. Fernández-Sáez, F. Arias, M. A. Gallo and M. D. Carrión, *Arch. Pharm.*, 2016, **349**, 638–650.

4 C. M. Grombein, Q. Hu, S. Rau, C. Zimmer and R. W. Hartmann, *Eur. J. Med. Chem.*, 2015, **90**, 788–796.

5 M. J. Kornet, *J. Heterocycl. Chem.*, 1992, **29**, 103–105.

6 R. Zhou, X. Qi and X.-F. Wu, *ACS Comb. Sci.*, 2019, **21**, 573–577.

7 D. Shi, G. Dou and Z.-Y. Li, *J. Chem. Res.*, 2007, **2007**, 545–547.

8 P. V. Fish, P. Filippakopoulos, G. Bish, P. E. Brennan, M. E. Bunnage, A. S. Cook, O. Federov, B. S. Gerstenberger, H. Jones, S. Knapp, B. Marsden, K. Nocka, D. R. Owen, M. Philpott, S. Picaud, M. J. Primiano, M. J. Ralph, N. Sciammetta and J. D. Trzupek, *J. Med. Chem.*, 2012, **55**, 9831–9837.

9 P. Nordeman, L. R. Odell and M. Larhed, *J. Org. Chem.*, 2012, **77**, 11393–11398.
